# Supplementary material for: Effects of shokyo (Zingiberis Rhizoma) and kankyo (Zingiberis Processum Rhizoma) on prostaglandin E2 production in lipopolysaccharide-treated mouse macrophage RAW264.7 cells
Source: PeerJ. 2019 Sep 17;7:e7725. doi: 10.7717/peerj.7725 (PMC6753926; doi:10.7717/peerj.7725)
Supplement: Supplemental Information 1 [file peerj-07-7725-s001.pdf]

A

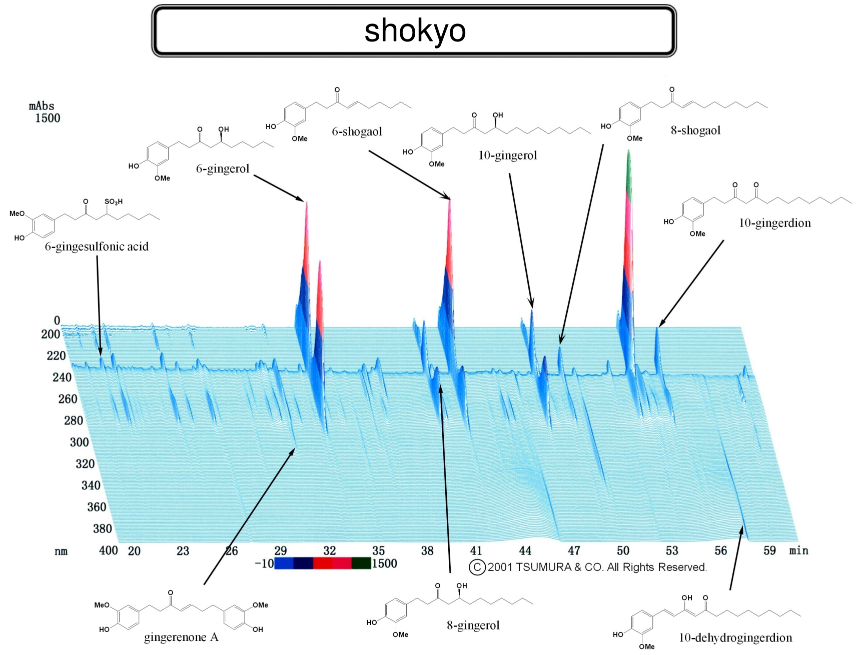

B

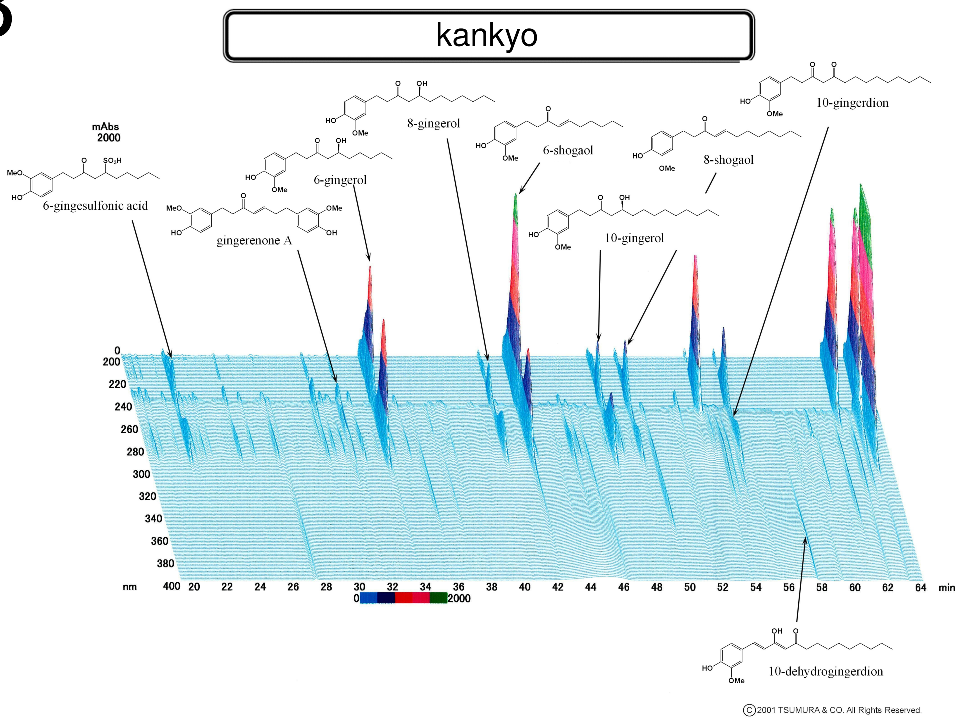

Supplemental Figure 1: 3D-HPLC profiles of shokyo (A) and kankyo (B).

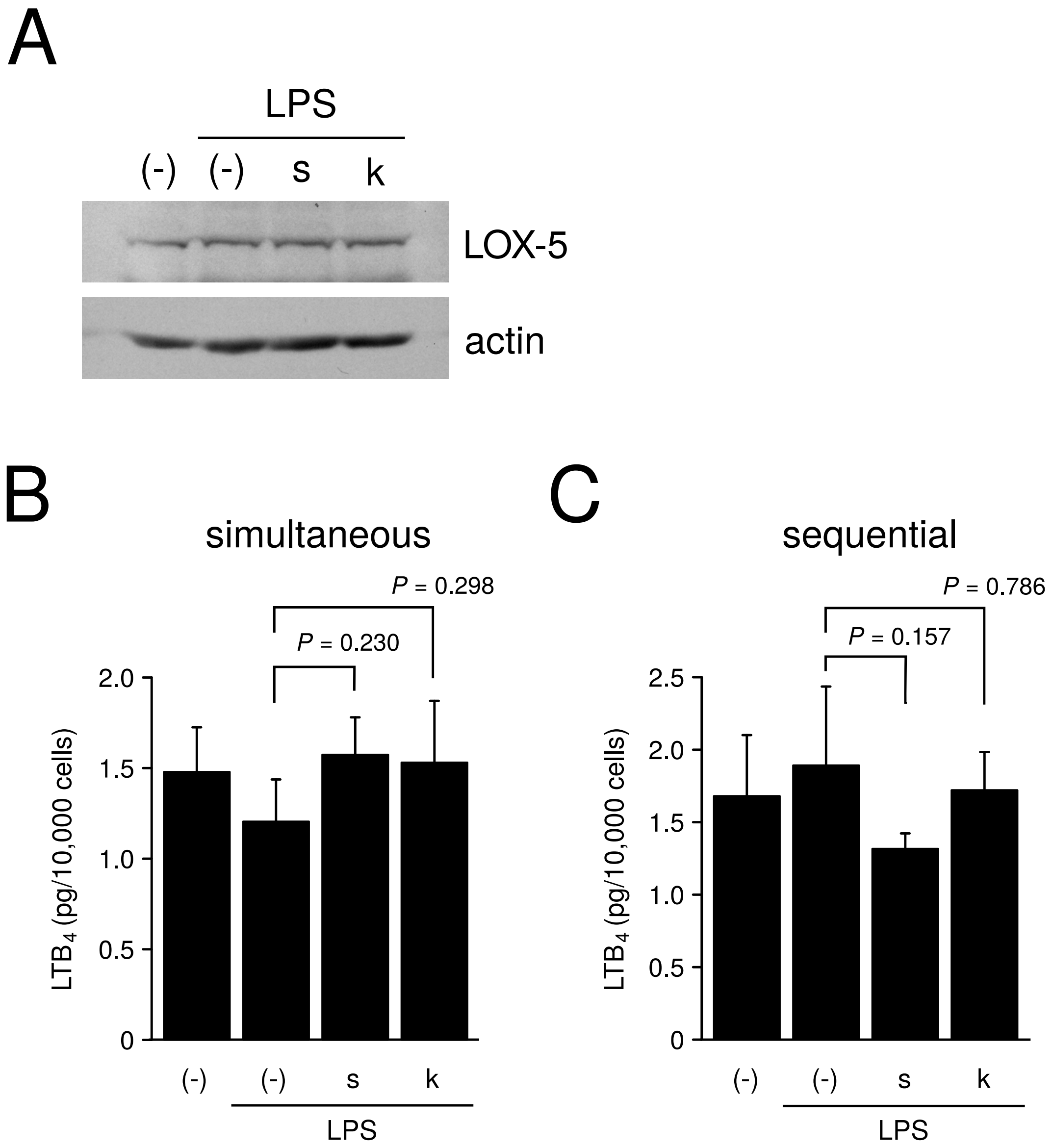

Supplemental Figure 2: Effect of shokyo and kankyo on lipoxygenase (LOX) pathway in RAW264.7 cells. (A) LOX-5 expression, and (B) LOX activity. The same samples in Figure 3 were used. s: shokyo, k: kankyo.

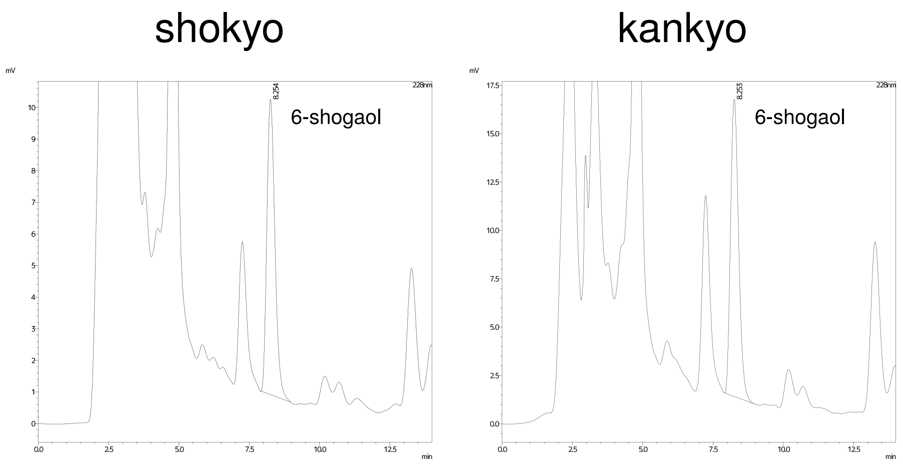

Analysis of [6]-Shogaol standard

conc. (uM)	Peak Area
0.1	5732
1	62025
10	629091
50	3184242

slope	63729
intercept	-3193.7

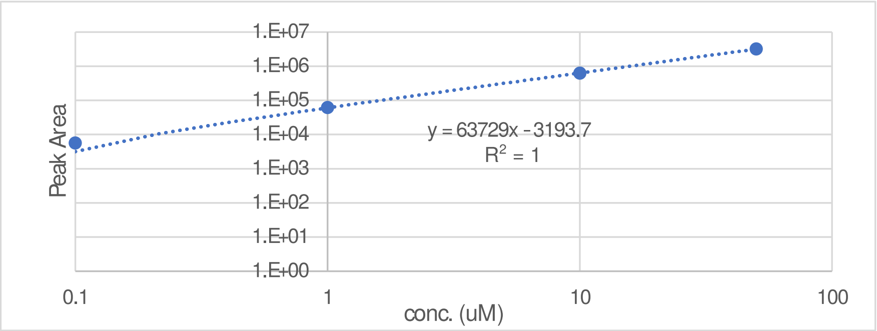

Results

	ID	sample (mL)	Constant volume (mL)	Peak Area	[6]-Shogaol conc. (uM)
shokyo	4412	1	1	186030	2.97
kankyo	4413	1	1	307051	4.87

Supplemental Figure 3: Quantification of 6-shogaol in herbs by HPLC. Samples (5 mg/ml of shokyo and kankyo) in culture medium were subjected to HPLC as described in Materials and Methods.
