# Supplementary material for: Effects of shokyo (Zingiberis Rhizoma) and kankyo (Zingiberis Processum Rhizoma) on prostaglandin E2 production in lipopolysaccharide-treated mouse macrophage RAW264.7 cells
Source: PeerJ. 2019 Sep 17;7:e7725. doi: 10.7717/peerj.7725 (PMC6753926; doi:10.7717/peerj.7725)
Supplement: Data S1 [file peerj-07-7725-s002.zip › Fig1/Analysis_cytotoxicity.pdf]

Statistical analysis

Toshiaki Ara

1 cytotoxicity

1.1 shokyo

```
 Simultaneous Tests for General Linear Hypotheses

Multiple Comparisons of Means: Dunnett Contrasts


Fit: lm(formula = adjusted ~ drug1, data = dat)

Linear Hypotheses:
              Estimate Std. Error t value Pr(>|t|)
100 - 0 == 0    -5.543      2.456  -2.257  0.10512
300 - 0 == 0    -8.937      2.456  -3.639  0.00912 **
1000 - 0 == 0  -17.064      2.456  -6.948  < 0.001 ***
---
Signif. codes:  0 ‘***’ 0.001 ‘**’ 0.01 ‘*’ 0.05 ‘.’ 0.1 ‘ ’ 1
(Adjusted p values reported -- single-step method)
```

1.2 kankyo

```
 Simultaneous Tests for General Linear Hypotheses

Multiple Comparisons of Means: Dunnett Contrasts
```

```
Fit: lm(formula = adjusted ~ drug1, data = dat)

Linear Hypotheses:
              Estimate Std. Error t value Pr(>|t|)
100 - 0 == 0    1.8685     0.6023   3.102   0.0233 *
300 - 0 == 0    0.1038     0.6023   0.172   0.9962
1000 - 0 == 0 -12.2145     0.6023 -20.279   <0.001 ***
---
Signif. codes:  0 '***' 0.001 '**' 0.01 '*' 0.05 '.' 0.1 ' ' 1
(Adjusted p values reported -- single-step method)
```
