# Supplementary material for: Effects of shokyo (Zingiberis Rhizoma) and kankyo (Zingiberis Processum Rhizoma) on prostaglandin E2 production in lipopolysaccharide-treated mouse macrophage RAW264.7 cells
Source: PeerJ. 2019 Sep 17;7:e7725. doi: 10.7717/peerj.7725 (PMC6753926; doi:10.7717/peerj.7725)
Supplement: Data S1 [file peerj-07-7725-s002.zip › Fig1/030_shokyo_WST-4-cytotoxicity.pdf]

- Exp. 30
- Condition
  - drug1: shokyo (ug/ml)
  - experimental No. 4
  - treatment: 24h
- Measurement
  - WST-8
  - Date: 2017.11.8
- Cells
  - cells: RAW264.7, passages: NA
  - cell numbers: 5×10^4 cells/well

	drug1	mean	SD
1	0	100.0	6.6
2	100	94.5	1.5
3	300	91.1	1.3
4	1000	82.9	1.1

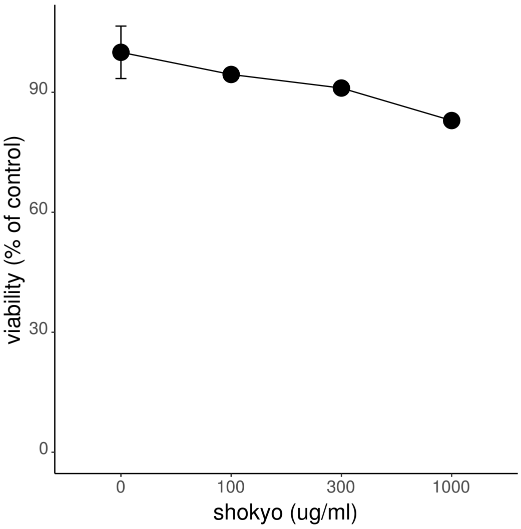

	OD	mean
1	0.050	0.049
2	0.050	
3	0.048	
4	0.048	
5	0.047	
6	0.050	
7	0.051	
8	0.047	

	drug1	OD	OD-blank	viability
1	0	0.926	0.877	109.2
2	0	0.846	0.797	99.3
3	0	0.832	0.783	97.5
4	0	0.803	0.754	93.9
5	100	0.823	0.774	96.4
6	100	0.811	0.762	94.9
7	100	0.798	0.749	93.3
8	100	0.797	0.748	93.2
9	300	0.794	0.745	92.8
10	300	0.781	0.732	91.2
11	300	0.776	0.727	90.6
12	300	0.769	0.720	89.7
13	1000	0.705	0.656	81.7
14	1000	0.726	0.677	84.3
15	1000	0.716	0.667	83.1
16	1000	0.712	0.663	82.6
