# Supplementary material for: Effects of shokyo (Zingiberis Rhizoma) and kankyo (Zingiberis Processum Rhizoma) on prostaglandin E2 production in lipopolysaccharide-treated mouse macrophage RAW264.7 cells
Source: PeerJ. 2019 Sep 17;7:e7725. doi: 10.7717/peerj.7725 (PMC6753926; doi:10.7717/peerj.7725)
Supplement: Data S1 [file peerj-07-7725-s002.zip › Fig1/030_kankyo_WST-4-cytotoxicity.pdf]

- Exp. 30
- Condition
  - drug1: kankyo (ug/ml)
  - experimental No. 4
  - treatment: 24h
- Measurement
  - WST-8
  - Date: 2017.11.8
- Cells
  - cells: RAW264.7, passages: NA
  - cell numbers: 5×10^4 cells/well

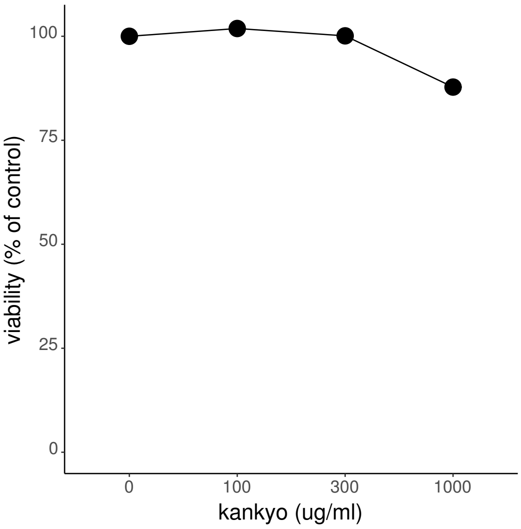

	drug1	mean	SD
1	0	100.0	0.6
2	100	101.9	0.6
3	300	100.1	0.5
4	1000	87.8	1.4

	OD	mean
1	0.048	0.050
2	0.049	
3	0.053	
4	0.049	
5	0.049	
6	0.050	
7	0.050	
8	0.048	

	drug1	OD	OD-blank	viability
1	0	0.769	0.720	99.6
2	0	0.767	0.718	99.3
3	0	0.776	0.727	100.6
4	0	0.776	0.727	100.6
5	100	0.784	0.735	101.7
6	100	0.781	0.732	101.2
7	100	0.786	0.737	101.9
8	100	0.791	0.742	102.6
9	300	0.775	0.726	100.4
10	300	0.775	0.726	100.4
11	300	0.768	0.719	99.4
12	300	0.773	0.724	100.1
13	1000	0.681	0.632	87.4
14	1000	0.694	0.644	89.2
15	1000	0.689	0.639	88.5
16	1000	0.671	0.622	86.0
