# Supplementary material for: Effects of shokyo (Zingiberis Rhizoma) and kankyo (Zingiberis Processum Rhizoma) on prostaglandin E2 production in lipopolysaccharide-treated mouse macrophage RAW264.7 cells
Source: PeerJ. 2019 Sep 17;7:e7725. doi: 10.7717/peerj.7725 (PMC6753926; doi:10.7717/peerj.7725)
Supplement: Data S2 [file peerj-07-7725-s003.zip › Fig2/030_kankyo_PGE2-3-ELISA.pdf]

- Exp. 30
- Condition
  - drug1: kankyo (ug/ml)
  - experimental No. 3
  - treatment: 24h
- Measurement
  - PGE2
  - Date: 2017.11.16
- Cells
  - cells: RAW264.7, passages: NA
  - cell numbers: 5×10^4 cells/well = 25×10^4 cells/ml

	conc.	OD
1	7.8	1.041
2	15.6	0.871
3	31.2	0.695
4	62.5	0.545
5	125.0	0.403
6	250.0	0.294
7	500.0	0.222
8	1000.0	0.197

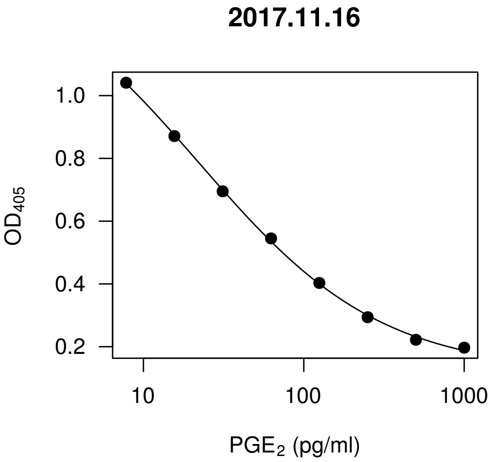

	drug1	mean	SD
1	0	0.144	0.019
2	1	0.148	0.019
3	10	0.114	0.012
4	100	0.015	0.004

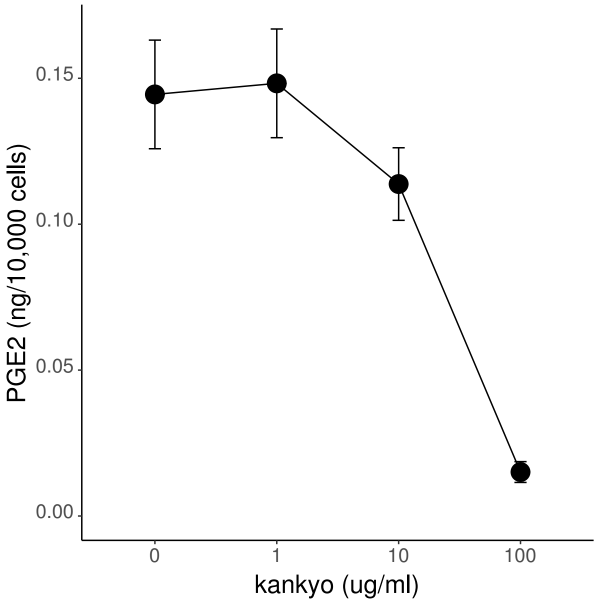

	drug1	viability	dilution	OD	conc. (pg/ml)	net (ng/ml)	(ng/10,000 cells)
1	0	101.06	10	0.256	378.17	3.782	0.150
2	0	99.84	10	0.251	399.15	3.992	0.160
3	0	99.10	10	0.277	306.73	3.067	0.124
4	1	101.19	10	0.269	331.19	3.312	0.131
5	1	101.68	10	0.245	426.92	4.269	0.168
6	1	95.41	10	0.264	348.10	3.481	0.146
7	10	100.45	10	0.277	306.73	3.067	0.122
8	10	99.71	10	0.280	298.29	2.983	0.120
9	10	98.98	10	0.302	246.15	2.462	0.099
10	100	95.29	10	0.672	34.87	0.349	0.015
11	100	98.49	10	0.603	46.39	0.464	0.019
12	100	93.82	10	0.731	27.55	0.275	0.012
