# Supplementary material for: Effects of shokyo (Zingiberis Rhizoma) and kankyo (Zingiberis Processum Rhizoma) on prostaglandin E2 production in lipopolysaccharide-treated mouse macrophage RAW264.7 cells
Source: PeerJ. 2019 Sep 17;7:e7725. doi: 10.7717/peerj.7725 (PMC6753926; doi:10.7717/peerj.7725)
Supplement: Data S2 [file peerj-07-7725-s003.zip › Fig2/Analysis_PGE2.pdf]

Statistical analysis

Toshiaki Ara

1 shokyo/kankyo -> PGE_2

1.1 simultanous treatment

```
 Simultaneous Tests for General Linear Hypotheses


Multiple Comparisons of Means: Dunnett Contrasts



Fit: lm(formula = adjusted ~ drug1, data = dat_sim)


Linear Hypotheses:
            Estimate Std. Error t value Pr(>|t|)
3 - 2 == 0 -0.089918   0.004599  -19.55 2.12e-06 ***
4 - 2 == 0 -0.098440   0.004599  -21.40 1.24e-06 ***
---
Signif. codes:  0 ‘***’ 0.001 ‘**’ 0.01 ‘*’ 0.05 ‘.’ 0.1 ‘ ’ 1
(Adjusted p values reported -- single-step method)
```

1.2 sequential treatment

```
 Simultaneous Tests for General Linear Hypotheses


Multiple Comparisons of Means: Dunnett Contrasts
```

```
Fit: lm(formula = adjusted ~ drug1, data = dat_seq)

Linear Hypotheses:
            Estimate Std. Error t value Pr(>|t|)
3 - 2 == 0 -0.089918   0.004599  -19.55 2.12e-06 ***
4 - 2 == 0 -0.098440   0.004599  -21.40 1.24e-06 ***
---
Signif. codes:  0 ‘***’ 0.001 ‘**’ 0.01 ‘*’ 0.05 ‘.’ 0.1 ‘ ’ 1
(Adjusted p values reported -- single-step method)
```

2 shokyo/kankyo -> PGE_2 (conc-dependent)

2.1 shokyo

```
 Simultaneous Tests for General Linear Hypotheses

Multiple Comparisons of Means: Dunnett Contrasts


Fit: lm(formula = adjusted ~ drug1, data = dat)

Linear Hypotheses:
              Estimate Std. Error t value Pr(>|t|)
1 - 0 == 0    0.007369   0.011620   0.634   0.8615
10 - 0 == 0  -0.038685   0.011620  -3.329   0.0258 *
100 - 0 == 0 -0.111654   0.011620  -9.609   <0.001 ***
---
Signif. codes:  0 ‘***’ 0.001 ‘**’ 0.01 ‘*’ 0.05 ‘.’ 0.1 ‘ ’ 1
(Adjusted p values reported -- single-step method)
```

2.2 kankyo

```
 Simultaneous Tests for General Linear Hypotheses

Multiple Comparisons of Means: Dunnett Contrasts


Fit: lm(formula = adjusted ~ drug1, data = dat)

Linear Hypotheses:
              Estimate Std. Error t value Pr(>|t|)
1 - 0 == 0    0.003798   0.011976   0.317    0.977
10 - 0 == 0  -0.030709   0.011976  -2.564    0.080 .
100 - 0 == 0 -0.129395   0.011976 -10.805   <0.001 ***
---
Signif. codes:  0 ‘***’ 0.001 ‘**’ 0.01 ‘*’ 0.05 ‘.’ 0.1 ‘ ’ 1
(Adjusted p values reported -- single-step method)
```
