# Supplementary material for: Effects of shokyo (Zingiberis Rhizoma) and kankyo (Zingiberis Processum Rhizoma) on prostaglandin E2 production in lipopolysaccharide-treated mouse macrophage RAW264.7 cells
Source: PeerJ. 2019 Sep 17;7:e7725. doi: 10.7717/peerj.7725 (PMC6753926; doi:10.7717/peerj.7725)
Supplement: Data S2 [file peerj-07-7725-s003.zip › Fig2/030_shokyo_PGE2-3-ELISA.pdf]

- Exp. 30
- Condition
  - drug1: shokyo (ug/ml)
  - experimental No. 3
  - treatment: 24h
- Measurement
  - PGE2
  - Date: 2017.11.16
- Cells
  - cells: RAW264.7, passages: NA
  - cell numbers: 5×10^4 cells/well = 25×10^4 cells/ml

	conc.	OD
1	7.8	1.041
2	15.6	0.871
3	31.2	0.695
4	62.5	0.545
5	125.0	0.403
6	250.0	0.294
7	500.0	0.222
8	1000.0	0.197

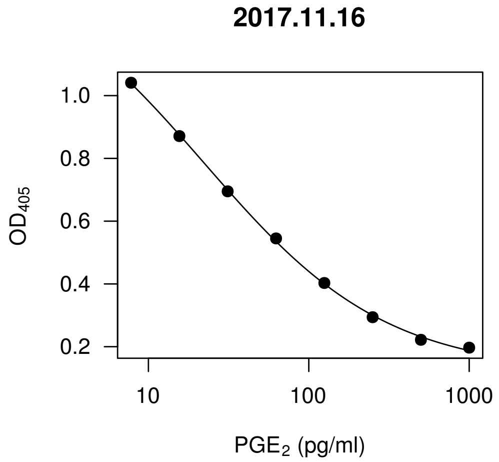

	drug1	mean	SD
1	0	0.139	0.021
2	1	0.146	0.017
3	10	0.100	0.008
4	100	0.027	0.005

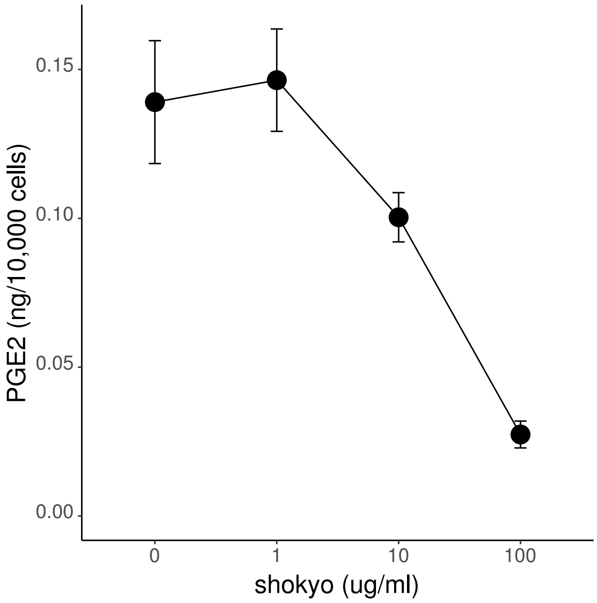

	drug1	viability	dilution	OD	conc. (pg/ml)	net (ng/ml)	(ng/10,000 cells)
1	0	99.18	10	0.255	382.23	3.822	0.154
2	0	99.42	10	0.259	366.42	3.664	0.147
3	0	101.40	10	0.282	292.86	2.929	0.116
4	1	99.92	10	0.260	362.64	3.626	0.145
5	1	100.54	10	0.248	412.66	4.127	0.164
6	1	100.04	10	0.271	324.79	3.248	0.130
7	10	98.43	10	0.291	270.26	2.703	0.110
8	10	100.78	10	0.306	238.21	2.382	0.095
9	10	98.56	10	0.306	238.21	2.382	0.097
10	100	99.30	10	0.541	60.86	0.609	0.025
11	100	101.77	10	0.531	63.71	0.637	0.025
12	100	101.77	10	0.476	82.90	0.829	0.033
