# Supplementary material for: Effects of shokyo (Zingiberis Rhizoma) and kankyo (Zingiberis Processum Rhizoma) on prostaglandin E2 production in lipopolysaccharide-treated mouse macrophage RAW264.7 cells
Source: PeerJ. 2019 Sep 17;7:e7725. doi: 10.7717/peerj.7725 (PMC6753926; doi:10.7717/peerj.7725)
Supplement: Data S2 [file peerj-07-7725-s003.zip › Fig2/030_herb_PGE2-4.pdf]

- Exp. 30
- Condition
  - drug1: herb ()
  - experimental No. 4
  - treatment: 24h
- Measurement
  - PGE2
  - Date: 2017.11.16
- Cells
  - cells: RAW264.7, passages: NA
  - cell numbers: 5×10^4 cells/well = 25×10^4 cells/ml

	conc.	OD
1	7.8	1.101
2	15.6	0.962
3	31.2	0.835
4	62.5	0.690
5	125.0	0.433
6	250.0	0.297
7	500.0	0.242
8	1000.0	0.205

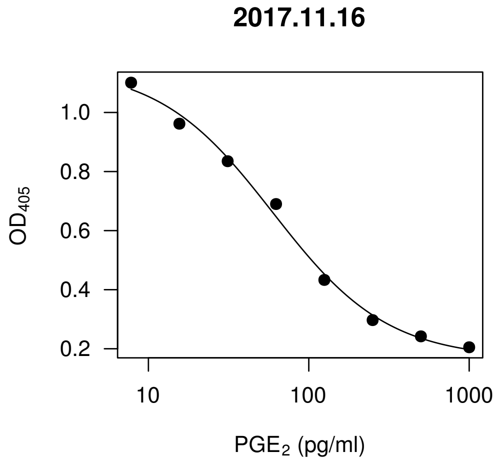

	drug1	mean	SD
1	1	0.002	0.001
2	2	0.113	0.009
3	3	0.023	0.003
4	4	0.015	0.003
5	5	0.004	0.001
6	6	0.038	0.004
7	7	0.011	0.001
8	8	0.006	0.002

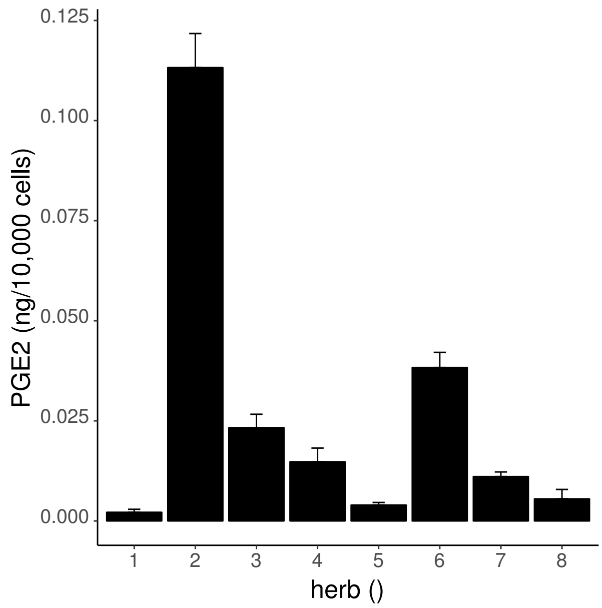

	drug1	viability	dilution	OD	conc. (pg/ml)	net (ng/ml)	(ng/10,000 cells)
1	1	99.74	10	1.085	7.20	0.072	0.003
2	1	99.74	10	1.125	3.48	0.035	0.001
3	1	100.51	10	1.103	5.56	0.056	0.002
4	2	105.38	10	0.281	317.53	3.175	0.121
5	2	111.40	10	0.293	289.31	2.893	0.104
6	2	100.38	10	0.293	289.31	2.893	0.115
7	3	103.33	10	0.662	59.50	0.595	0.023
8	3	110.25	10	0.683	55.48	0.555	0.020
9	3	94.36	10	0.644	63.17	0.632	0.027
10	4	104.48	10	0.750	44.24	0.442	0.017
11	4	110.25	10	0.857	29.93	0.299	0.011
12	4	94.11	10	0.786	39.01	0.390	0.017
13	5	94.49	5	0.927	22.18	0.111	0.005
14	5	103.97	5	0.968	18.05	0.090	0.003
15	5	95.64	5	0.969	17.95	0.090	0.004
16	6	99.10	5	0.385	168.35	0.842	0.034
17	6	105.12	5	0.339	214.42	1.072	0.041
18	6	89.75	5	0.371	180.42	0.902	0.040
19	7	96.93	5	0.719	49.17	0.246	0.010
20	7	104.48	5	0.680	56.03	0.280	0.011
21	7	83.86	5	0.703	51.89	0.259	0.012
22	8	92.95	5	0.835	32.59	0.163	0.007
23	8	90.26	5	0.853	30.40	0.152	0.007
24	8	79.63	5	1.041	11.19	0.056	0.003
