# Supplementary figures and images for: Effects of shokyo (Zingiberis Rhizoma) and kankyo (Zingiberis Processum Rhizoma) on prostaglandin E2 production in lipopolysaccharide-treated mouse macrophage RAW264.7 cells
Source: PeerJ. 2019 Sep 17;7:e7725. doi: 10.7717/peerj.7725 (PMC6753926; doi:10.7717/peerj.7725)

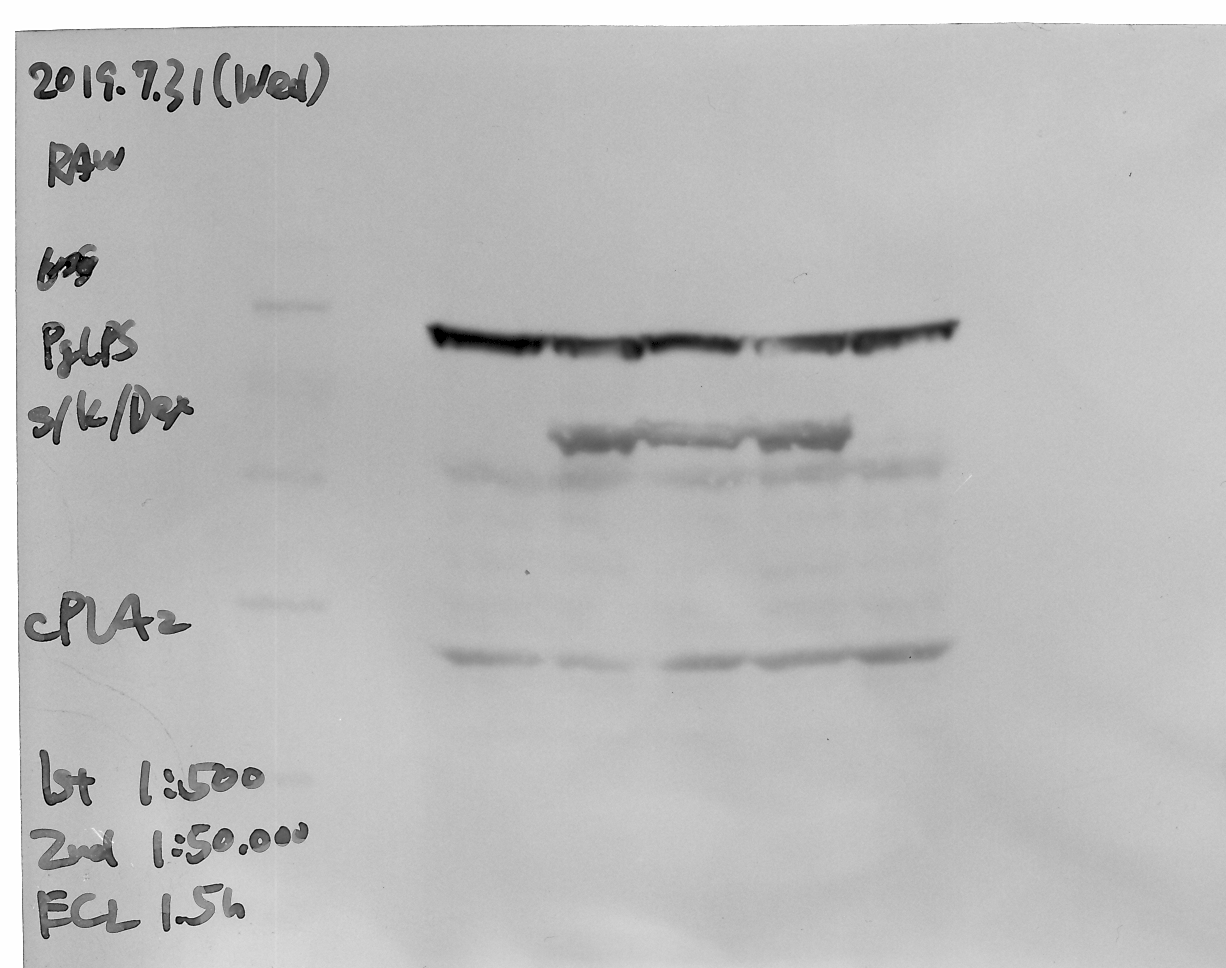

Supplement: Data S3 [file peerj-07-7725-s004.zip › Fig3/PLA2-20190731-1.png]

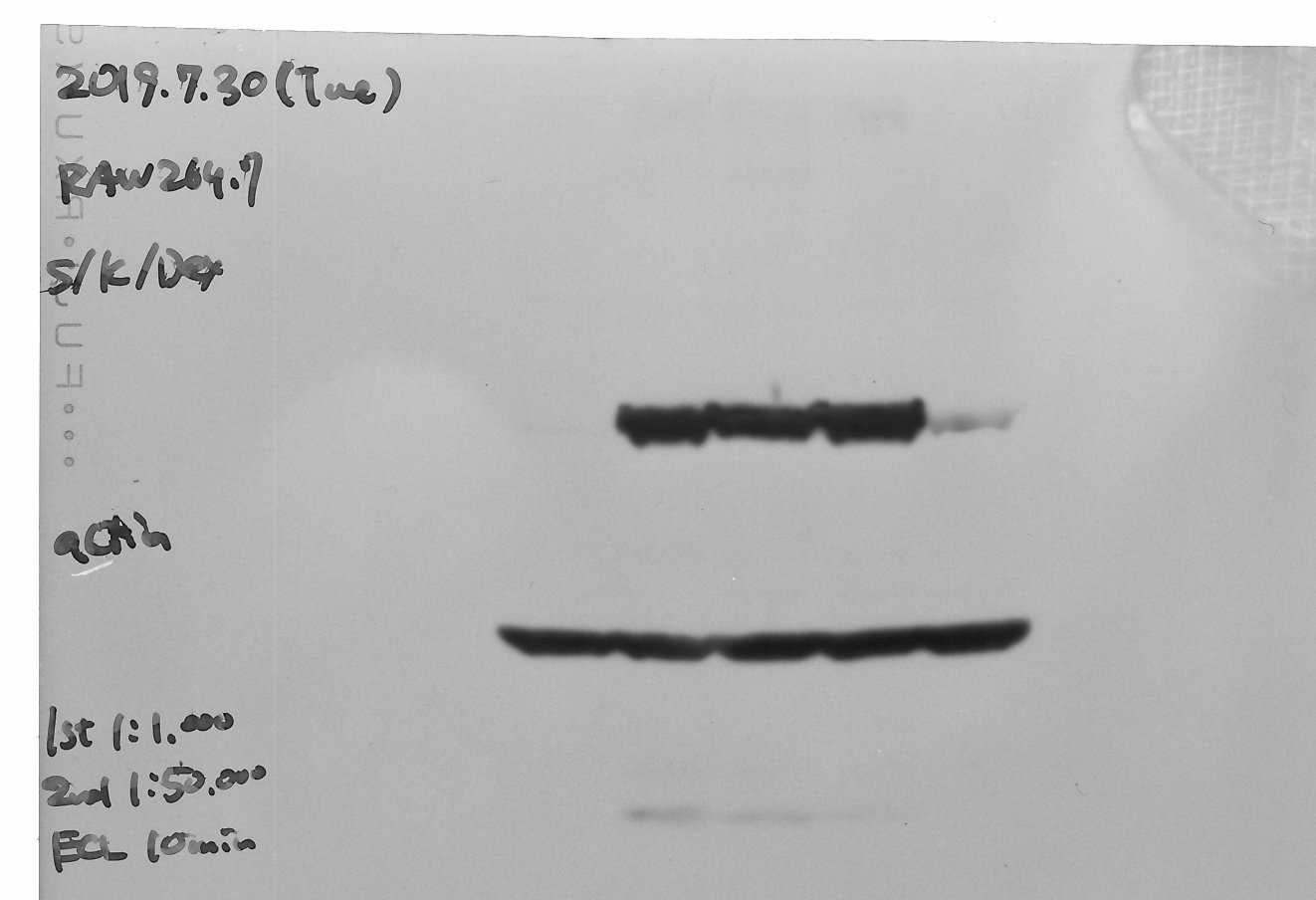

Supplement: Data S3 [file peerj-07-7725-s004.zip › Fig3/actin-20190730-1.png]

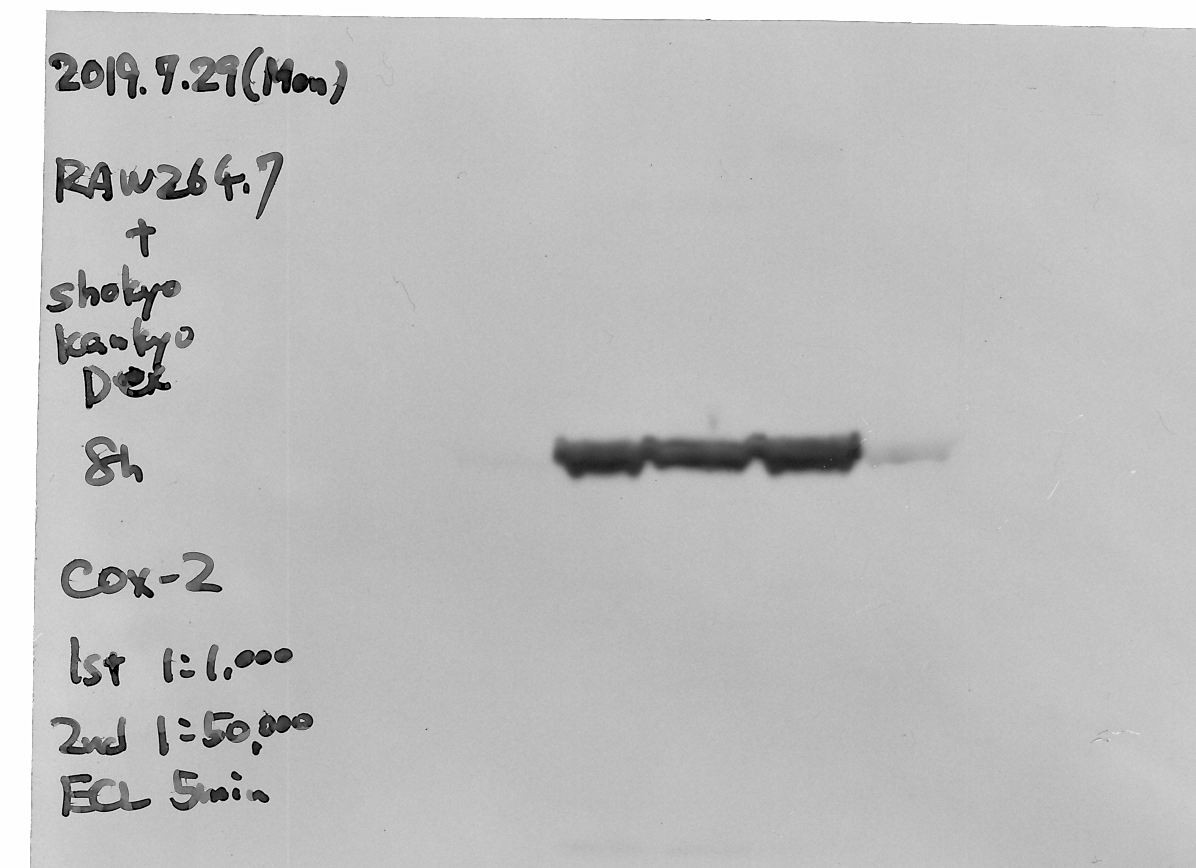

Supplement: Data S3 [file peerj-07-7725-s004.zip › Fig3/COX2-20190729-1.png]

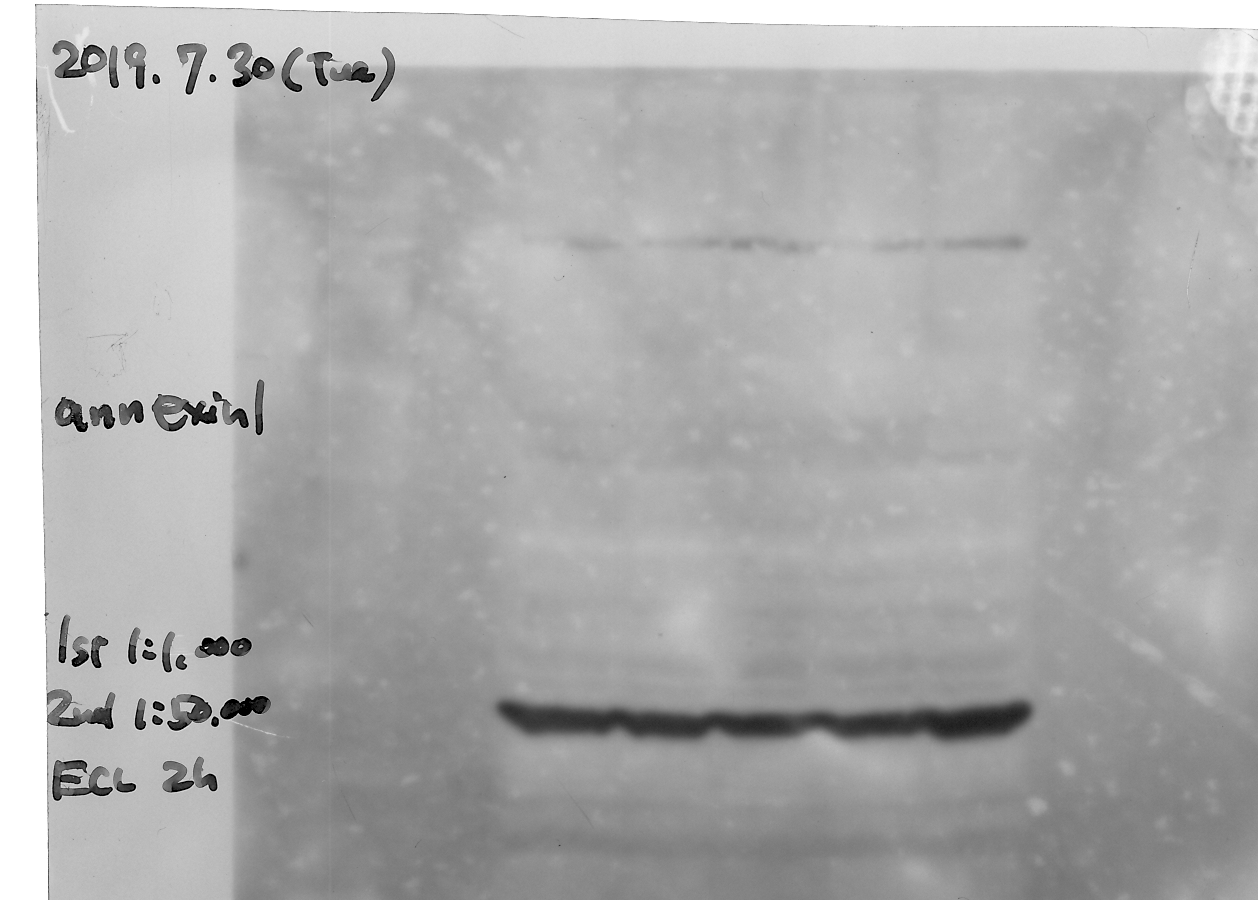

Supplement: Data S3 [file peerj-07-7725-s004.zip › Fig3/annexin1-20190730-1.png]

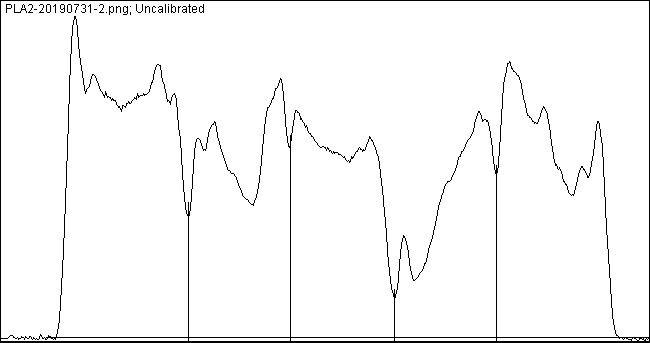

Supplement: Data S3 [file peerj-07-7725-s004.zip › analysis_ImageJ/Fig3D/PLA2.tif]

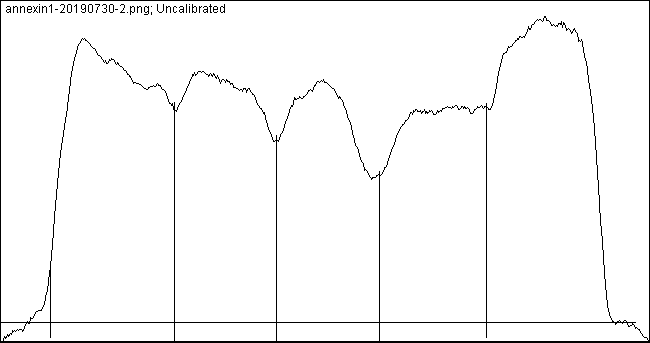

Supplement: Data S3 [file peerj-07-7725-s004.zip › analysis_ImageJ/Fig3D/annexin1.tif]

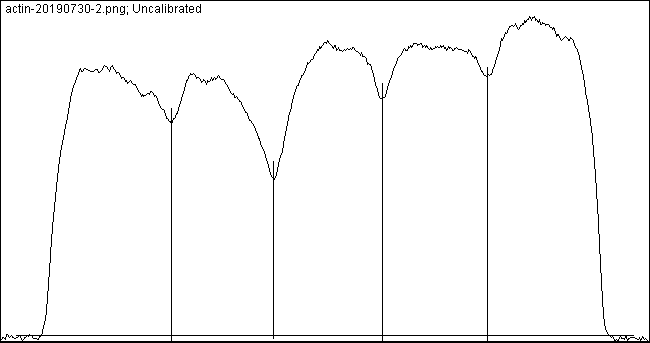

Supplement: Data S3 [file peerj-07-7725-s004.zip › analysis_ImageJ/Fig3D/actin.tif]

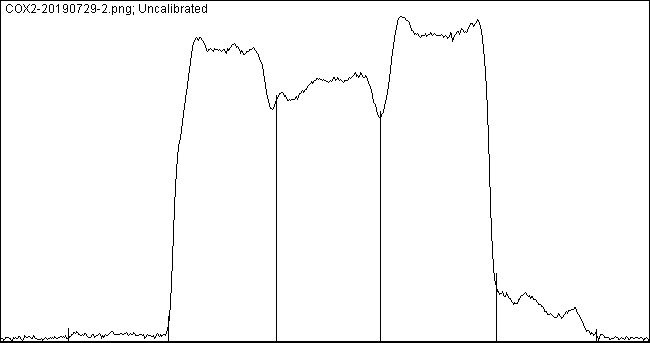

Supplement: Data S3 [file peerj-07-7725-s004.zip › analysis_ImageJ/Fig3D/COX2.tif]

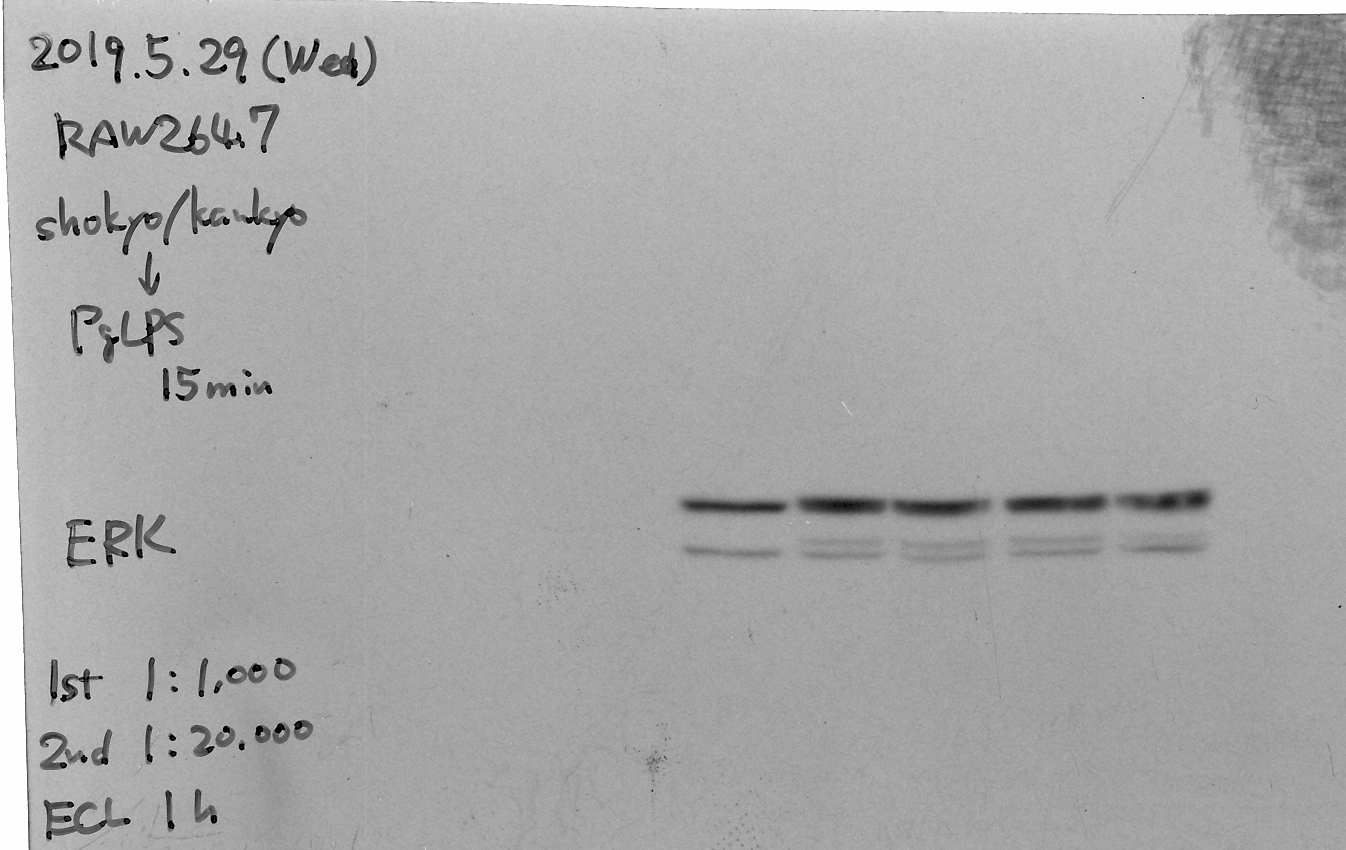

Supplement: Data S4 [file peerj-07-7725-s005.zip › Fig4/ERK-20190529-1.png]

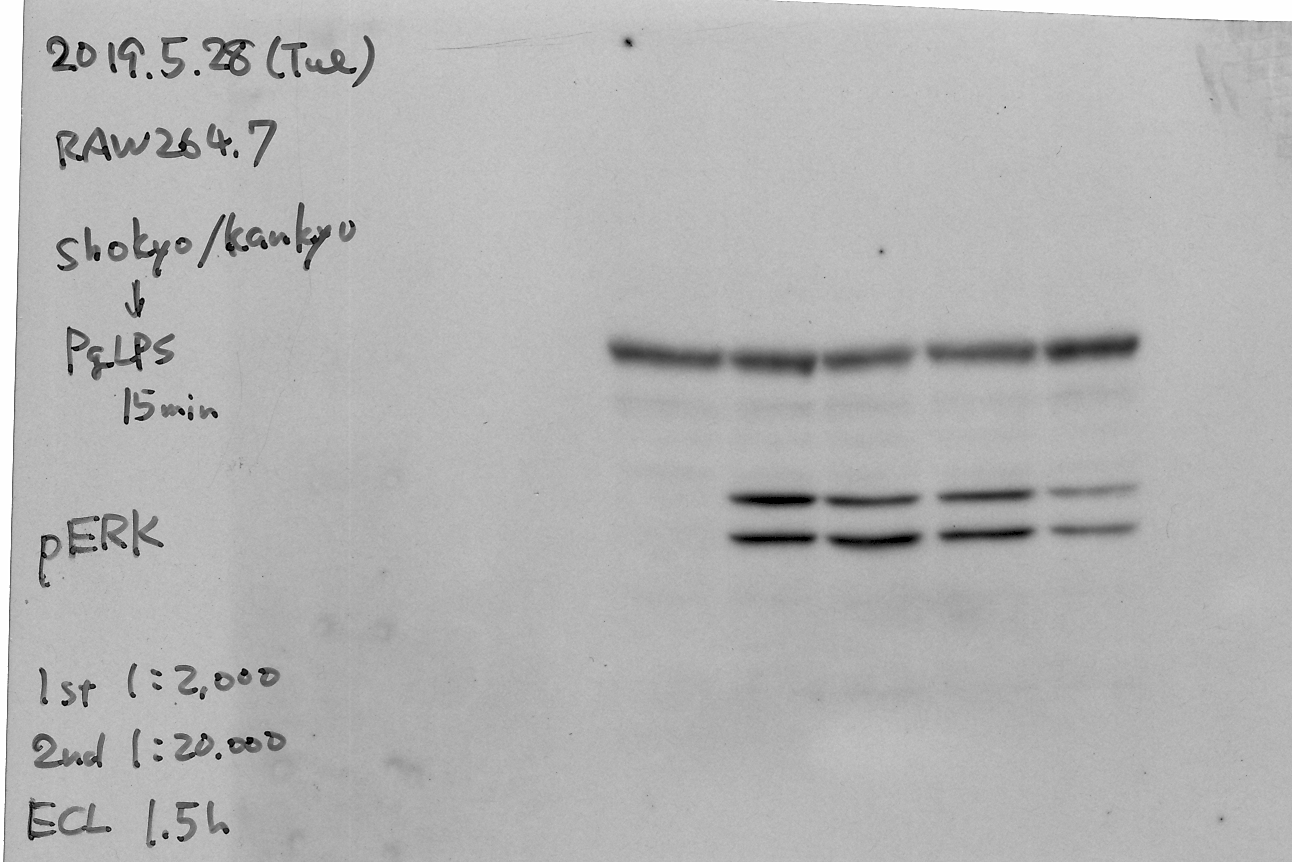

Supplement: Data S4 [file peerj-07-7725-s005.zip › Fig4/pERK-20190528-1.png]

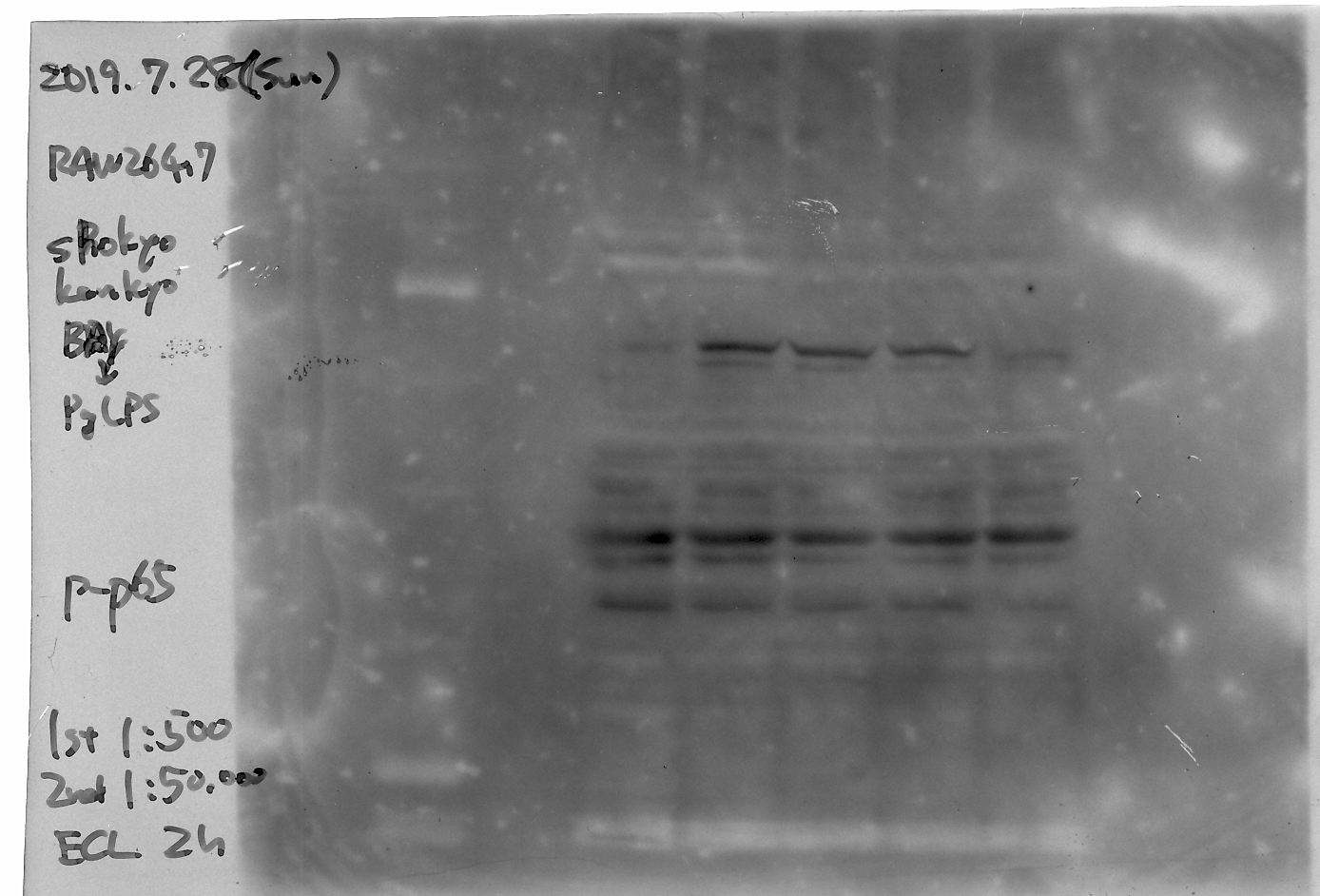

Supplement: Data S4 [file peerj-07-7725-s005.zip › Fig4/p-p65-20190728-1.png]

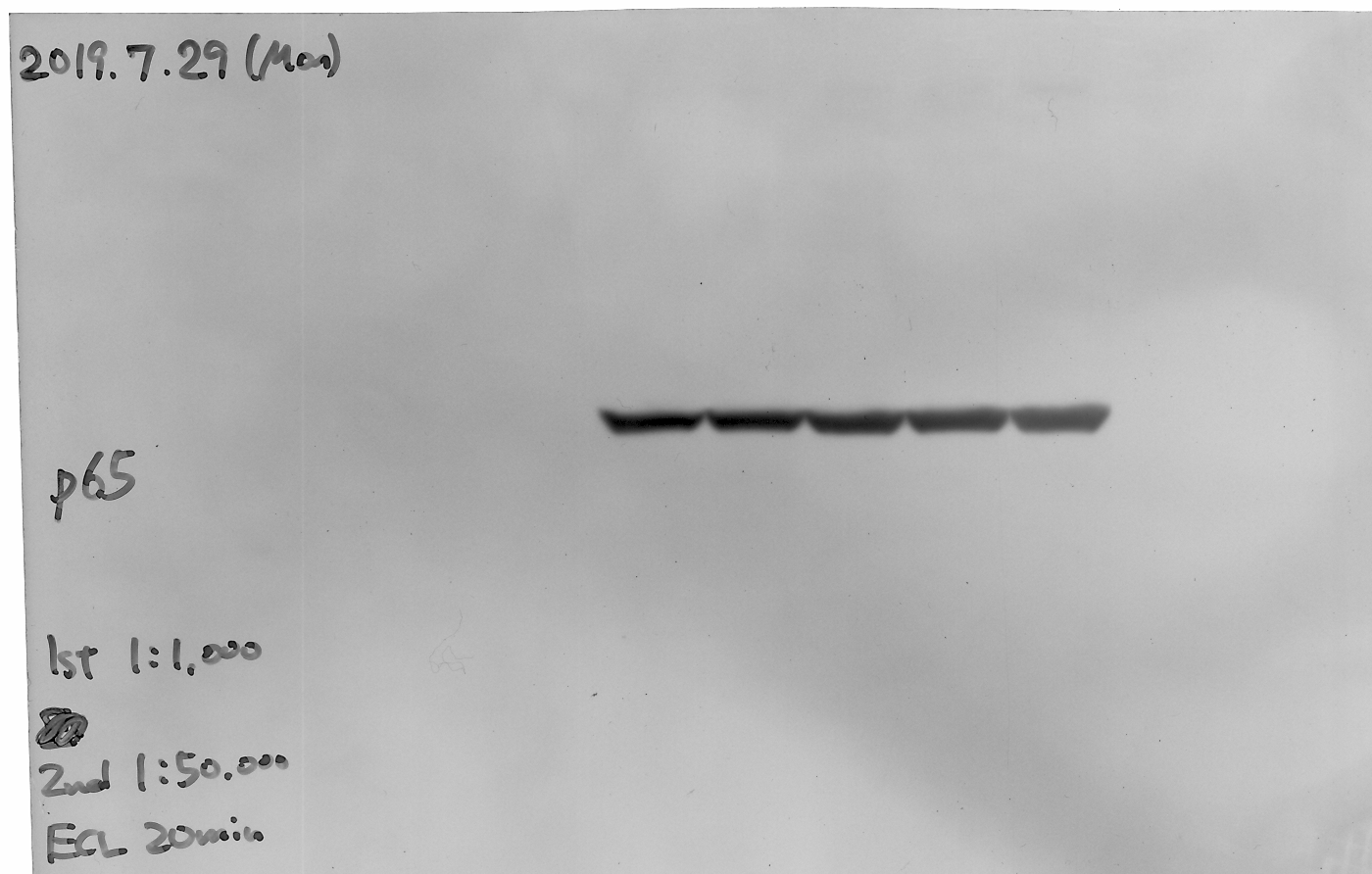

Supplement: Data S4 [file peerj-07-7725-s005.zip › Fig4/p65-20190729-1.png]

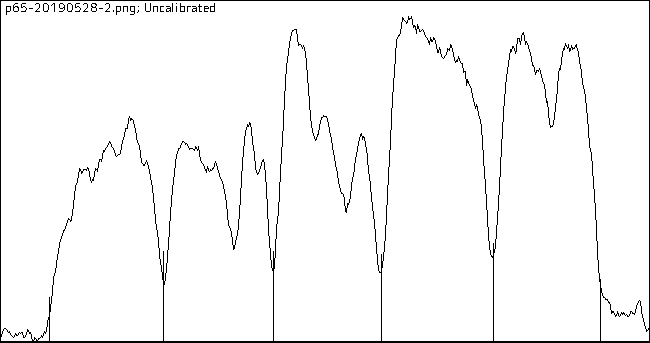

Supplement: Data S4 [file peerj-07-7725-s005.zip › analysis_ImageJ/Fig4B/p65.tif]

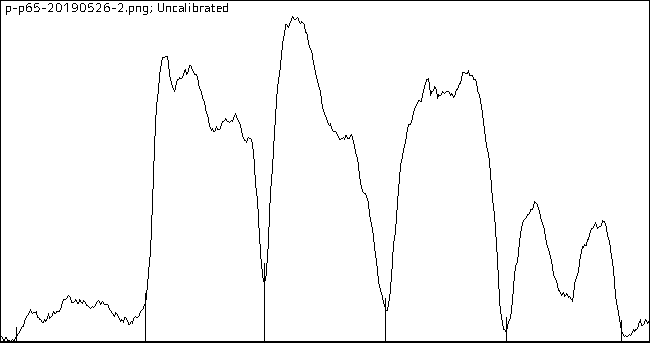

Supplement: Data S4 [file peerj-07-7725-s005.zip › analysis_ImageJ/Fig4B/p-p65.tif]

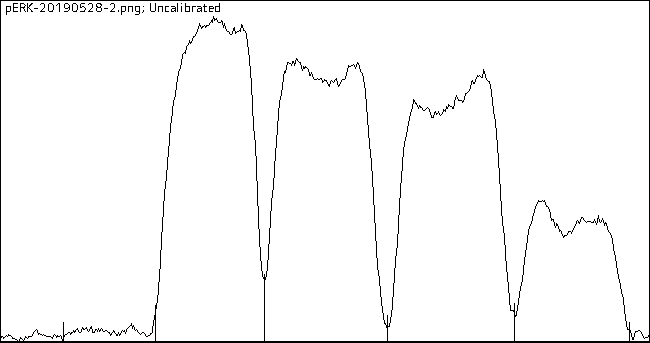

Supplement: Data S4 [file peerj-07-7725-s005.zip › analysis_ImageJ/Fig4C/pERK.tif]

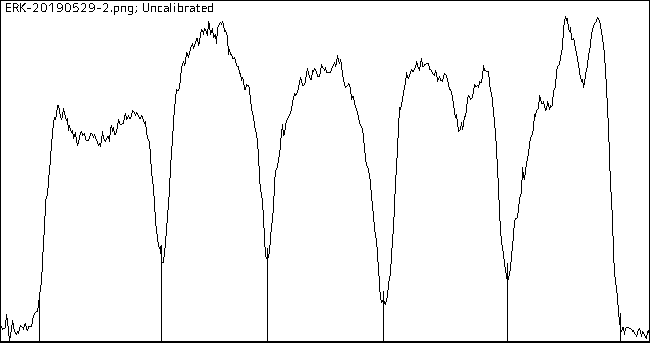

Supplement: Data S4 [file peerj-07-7725-s005.zip › analysis_ImageJ/Fig4C/ERK.tif]

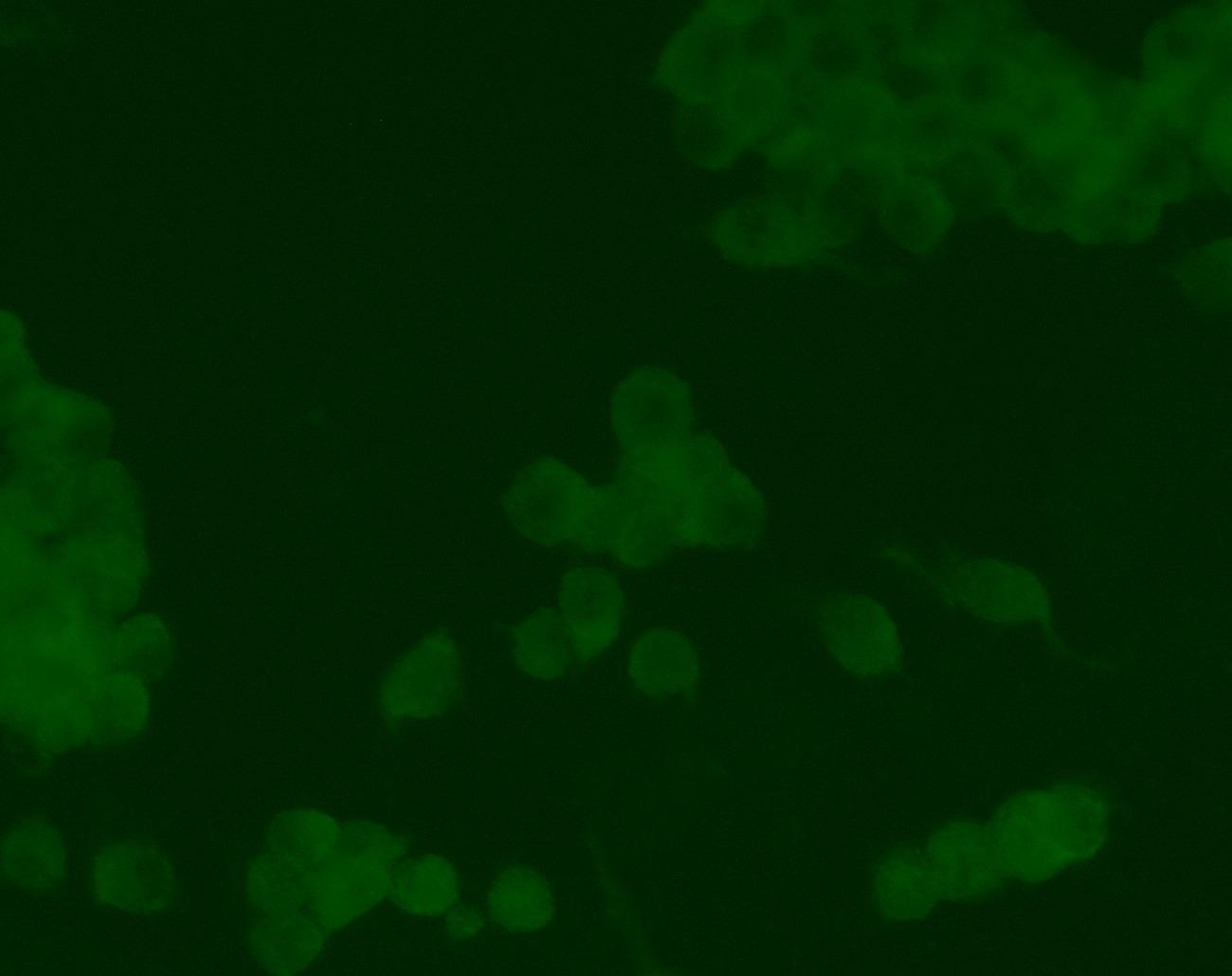

Supplement: Data S5 [file peerj-07-7725-s006.zip › Fig4_IF/05-1_FITC.jpg]

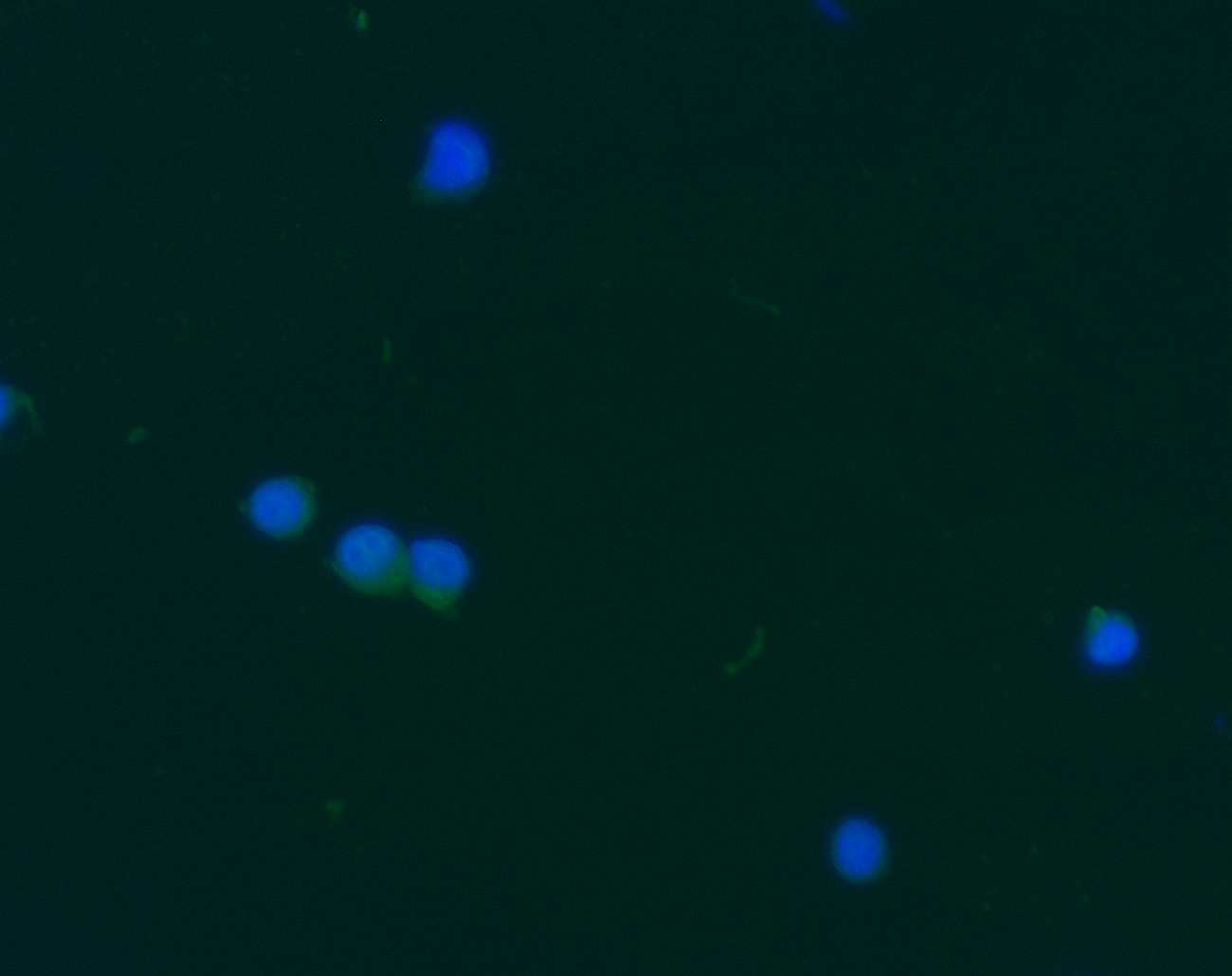

Supplement: Data S5 [file peerj-07-7725-s006.zip › Fig4_IF/03-1_(DAPI+FITC).jpg]

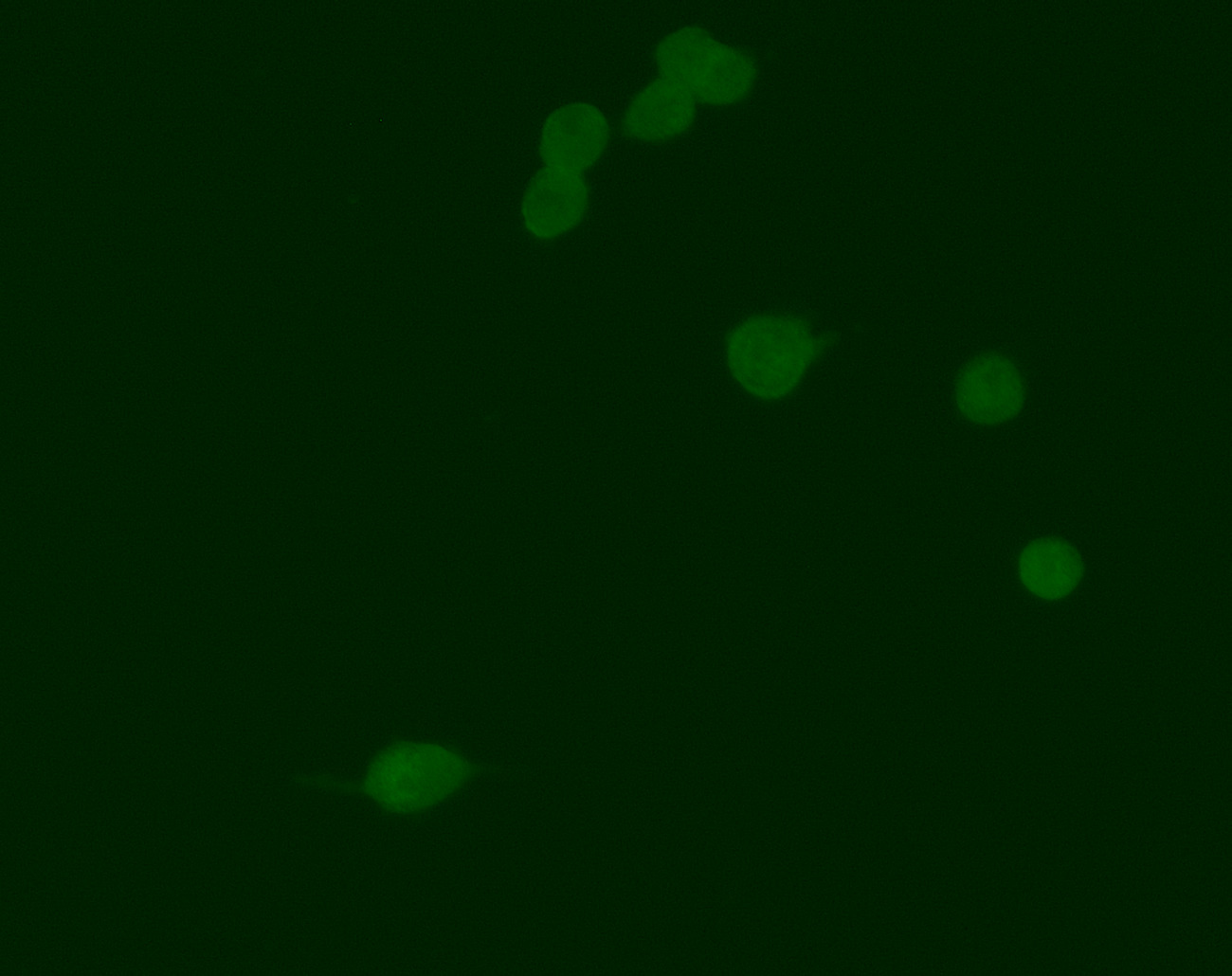

Supplement: Data S5 [file peerj-07-7725-s006.zip › Fig4_IF/02-1_FITC.jpg]

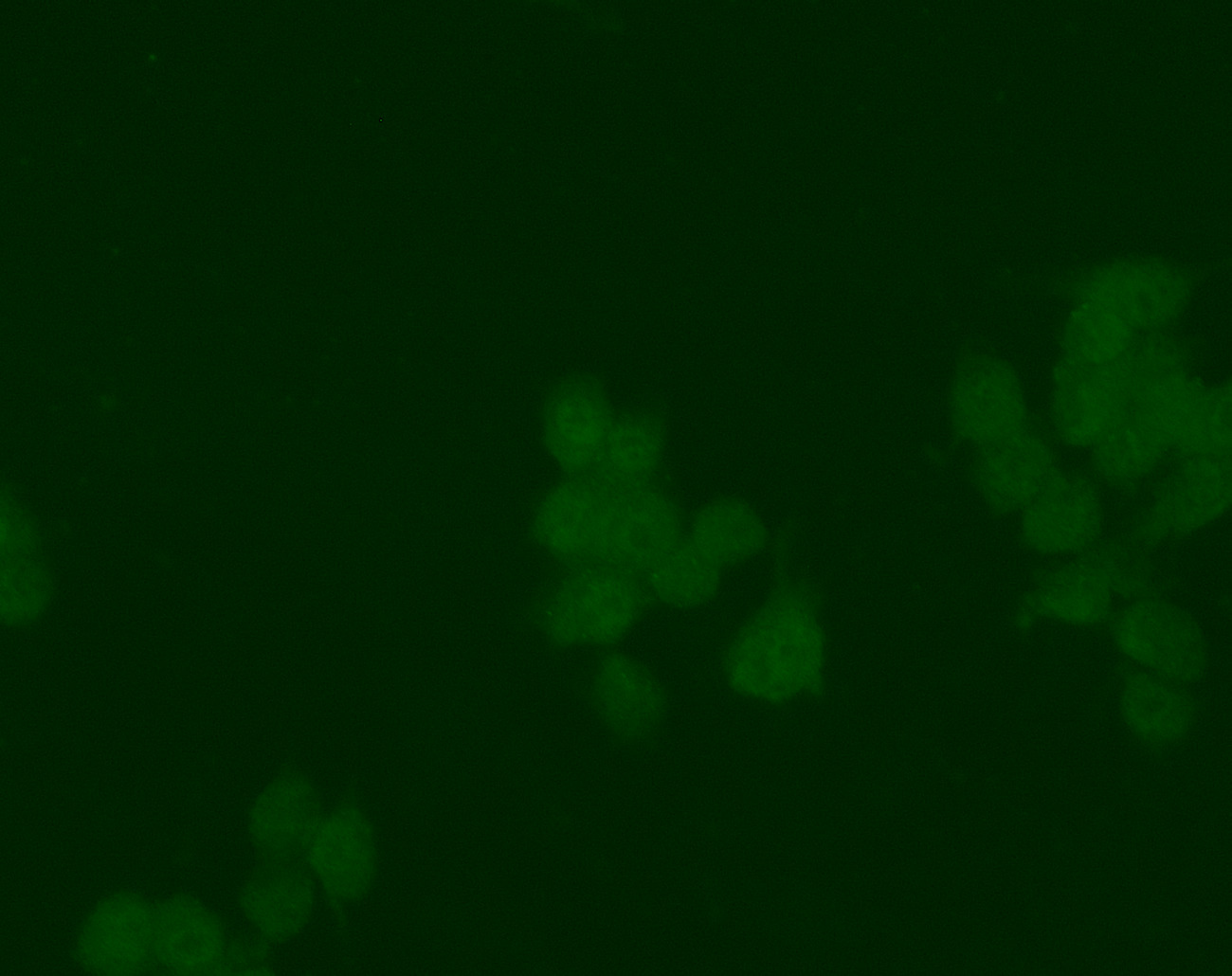

Supplement: Data S5 [file peerj-07-7725-s006.zip › Fig4_IF/04-2_FITC.jpg]

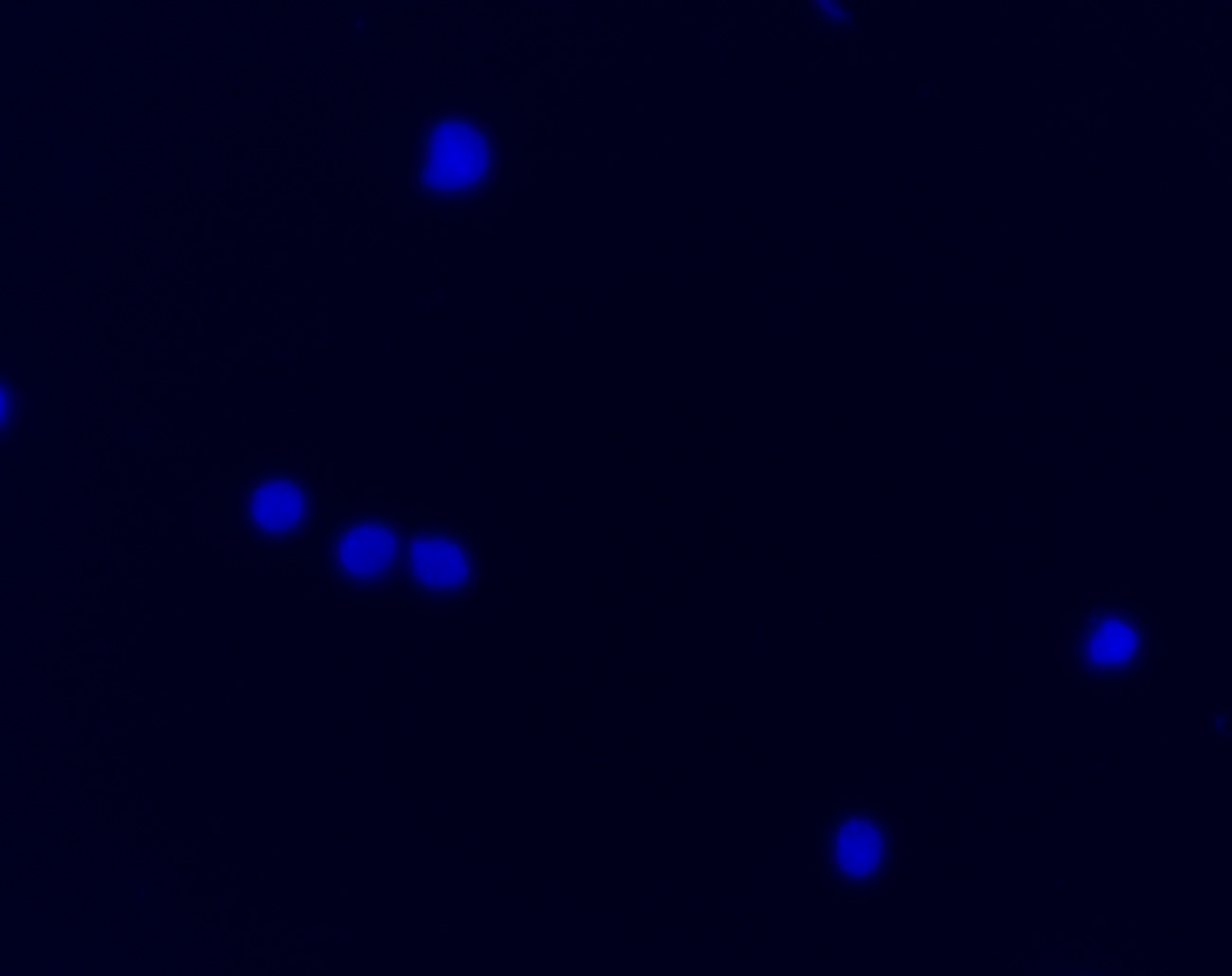

Supplement: Data S5 [file peerj-07-7725-s006.zip › Fig4_IF/03-1_DAPI.jpg]

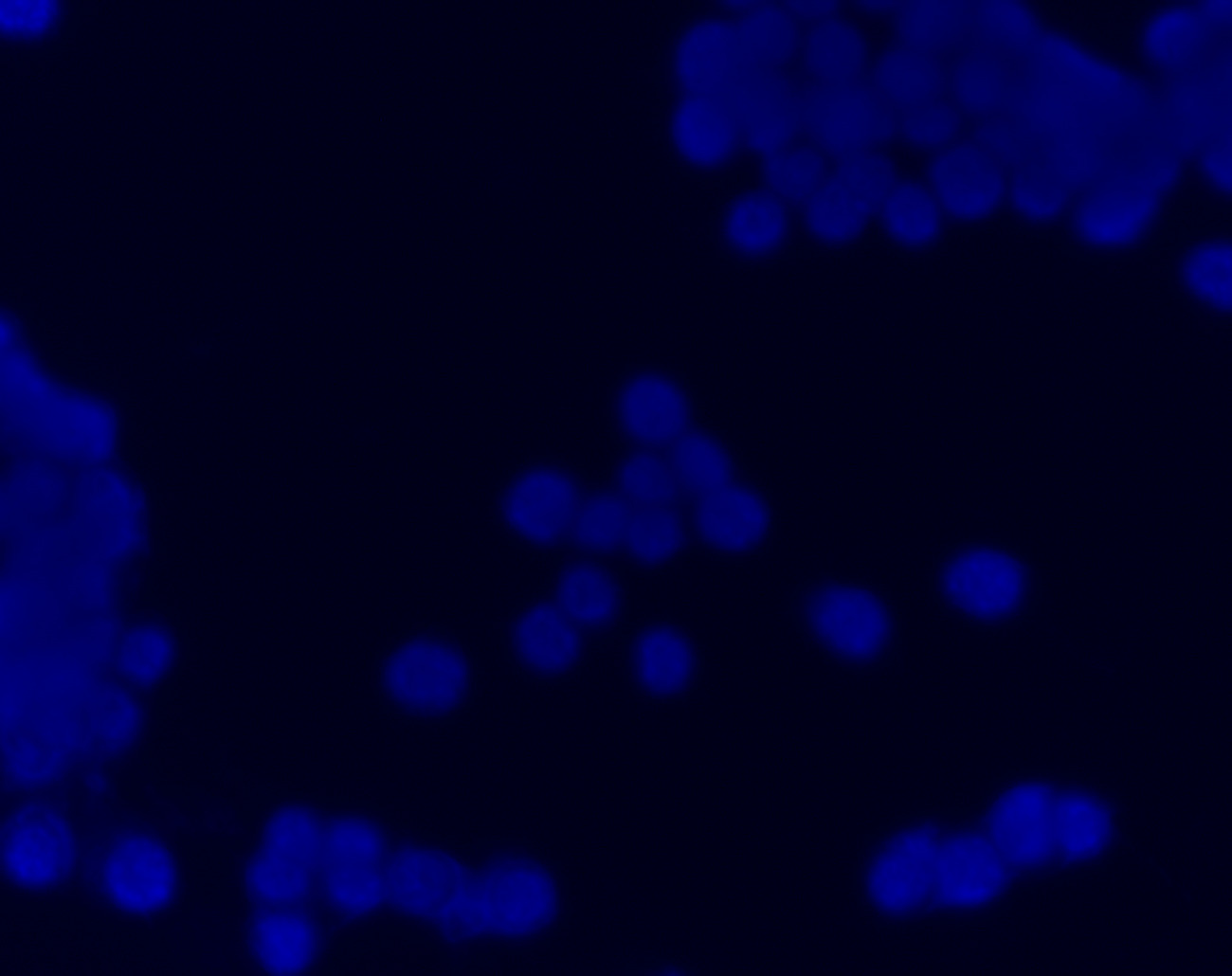

Supplement: Data S5 [file peerj-07-7725-s006.zip › Fig4_IF/05-1_DAPI.jpg]

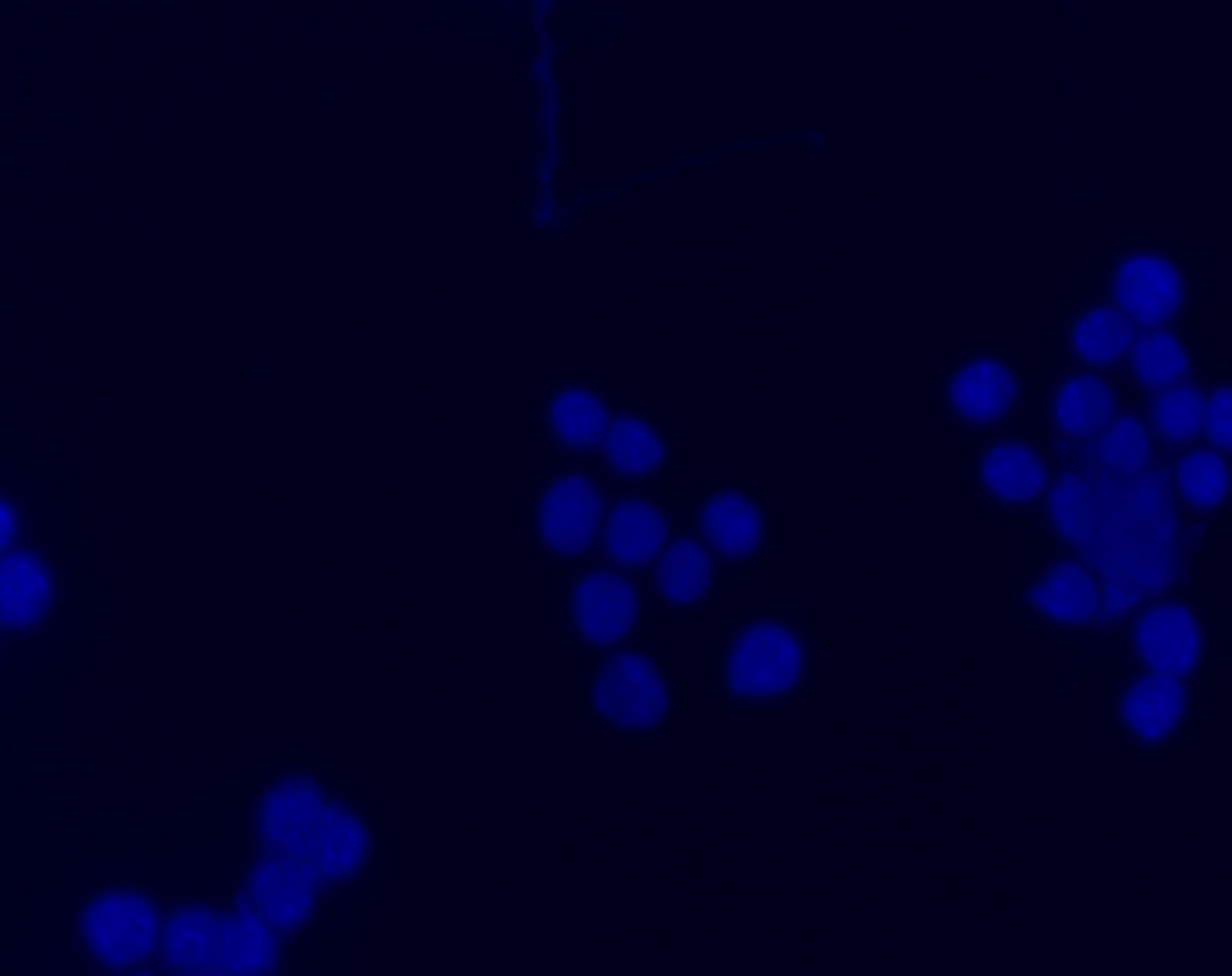

Supplement: Data S5 [file peerj-07-7725-s006.zip › Fig4_IF/04-2_DAPI.jpg]

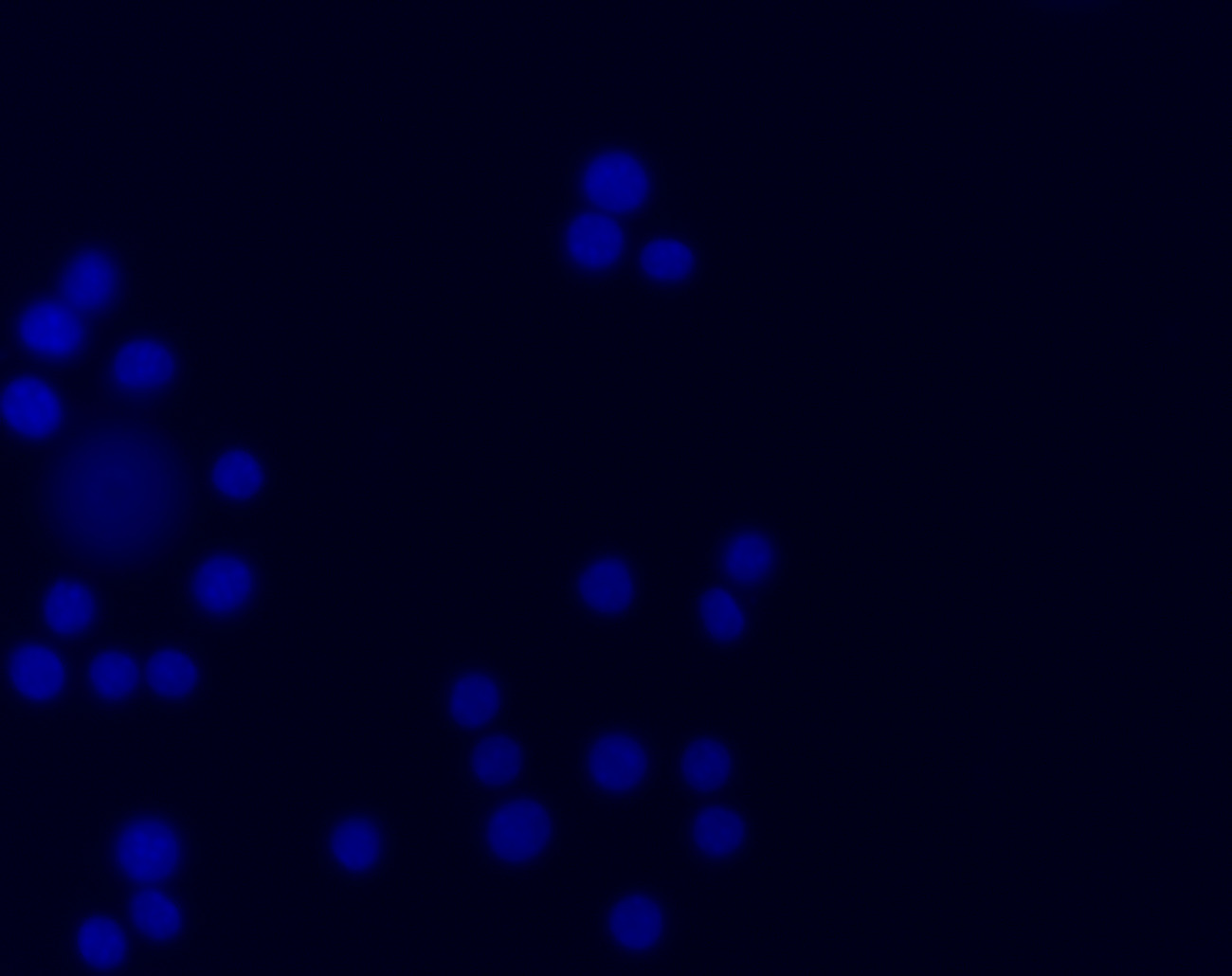

Supplement: Data S5 [file peerj-07-7725-s006.zip › Fig4_IF/01-2_DAPI.jpg]

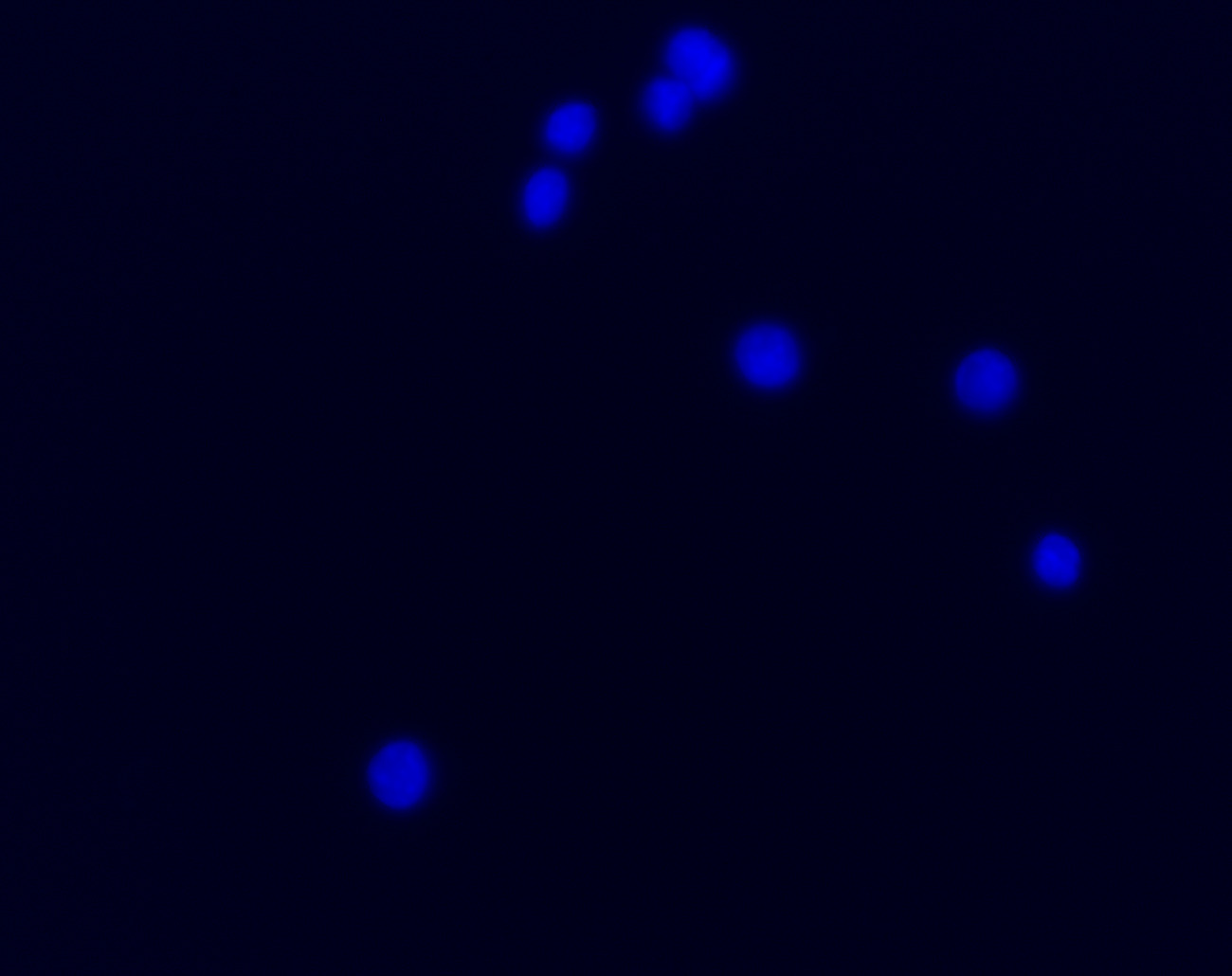

Supplement: Data S5 [file peerj-07-7725-s006.zip › Fig4_IF/02-1_DAPI.jpg]

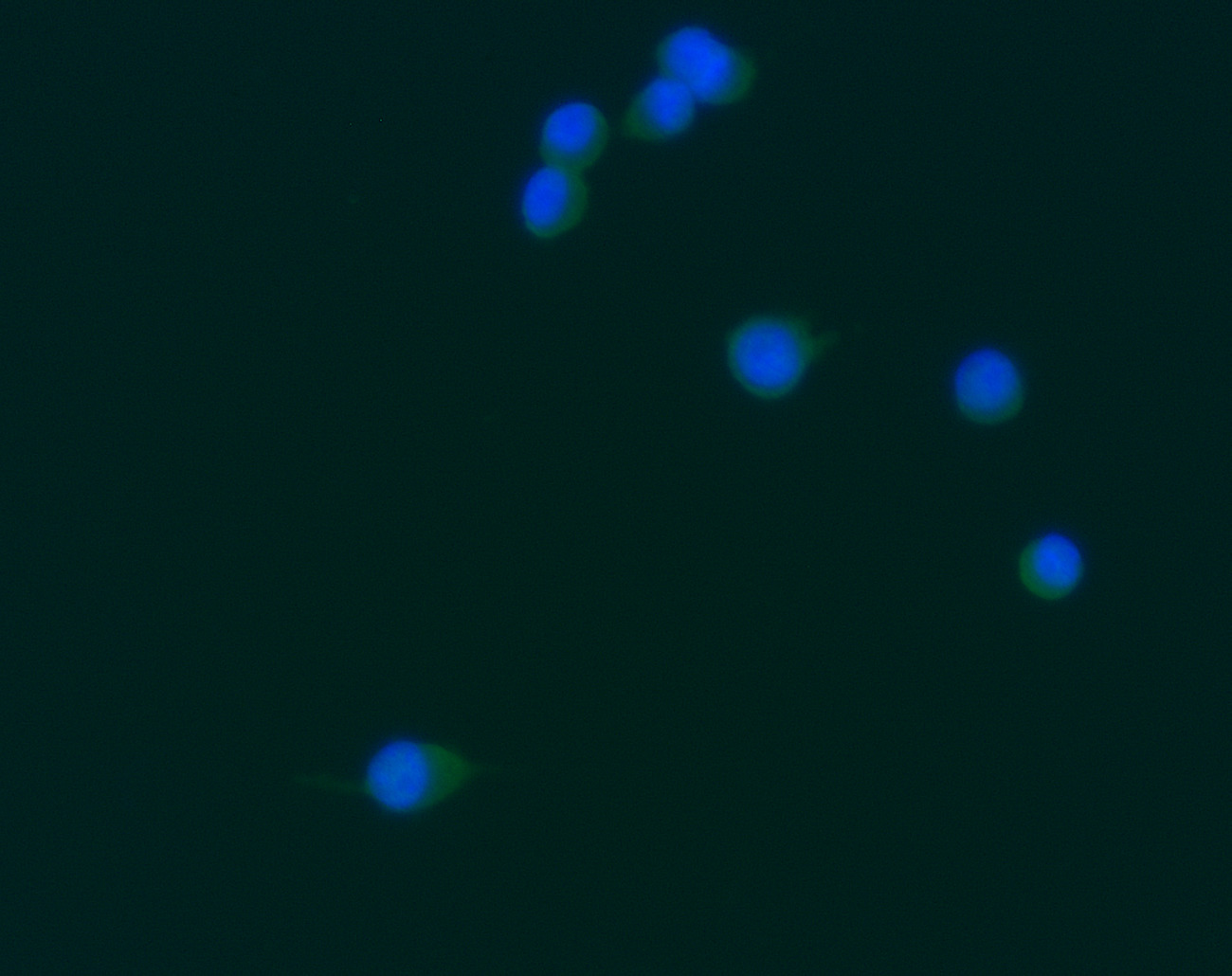

Supplement: Data S5 [file peerj-07-7725-s006.zip › Fig4_IF/02-1_(DAPI+FITC).jpg]

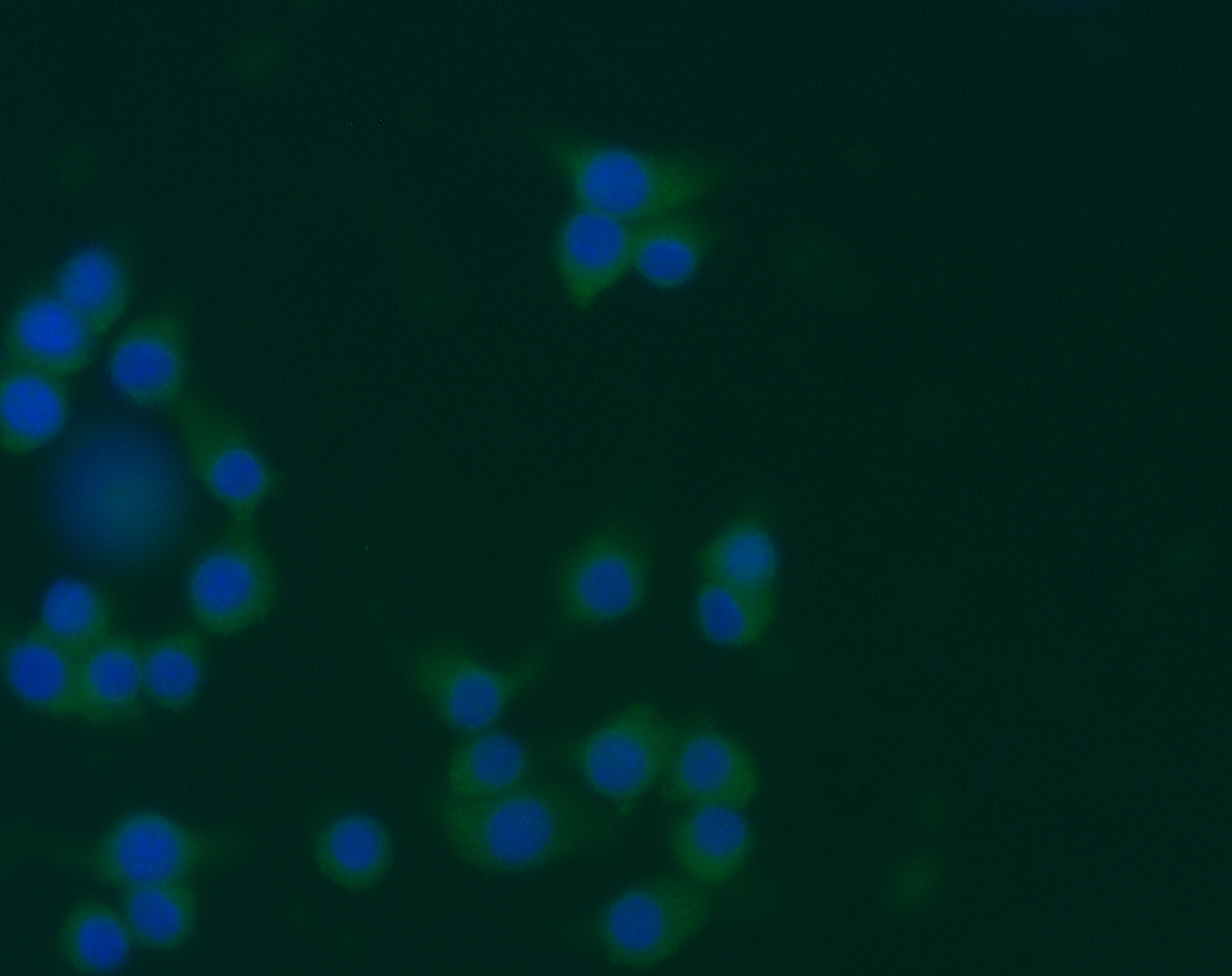

Supplement: Data S5 [file peerj-07-7725-s006.zip › Fig4_IF/01-2_(DAPI+FITC).jpg]

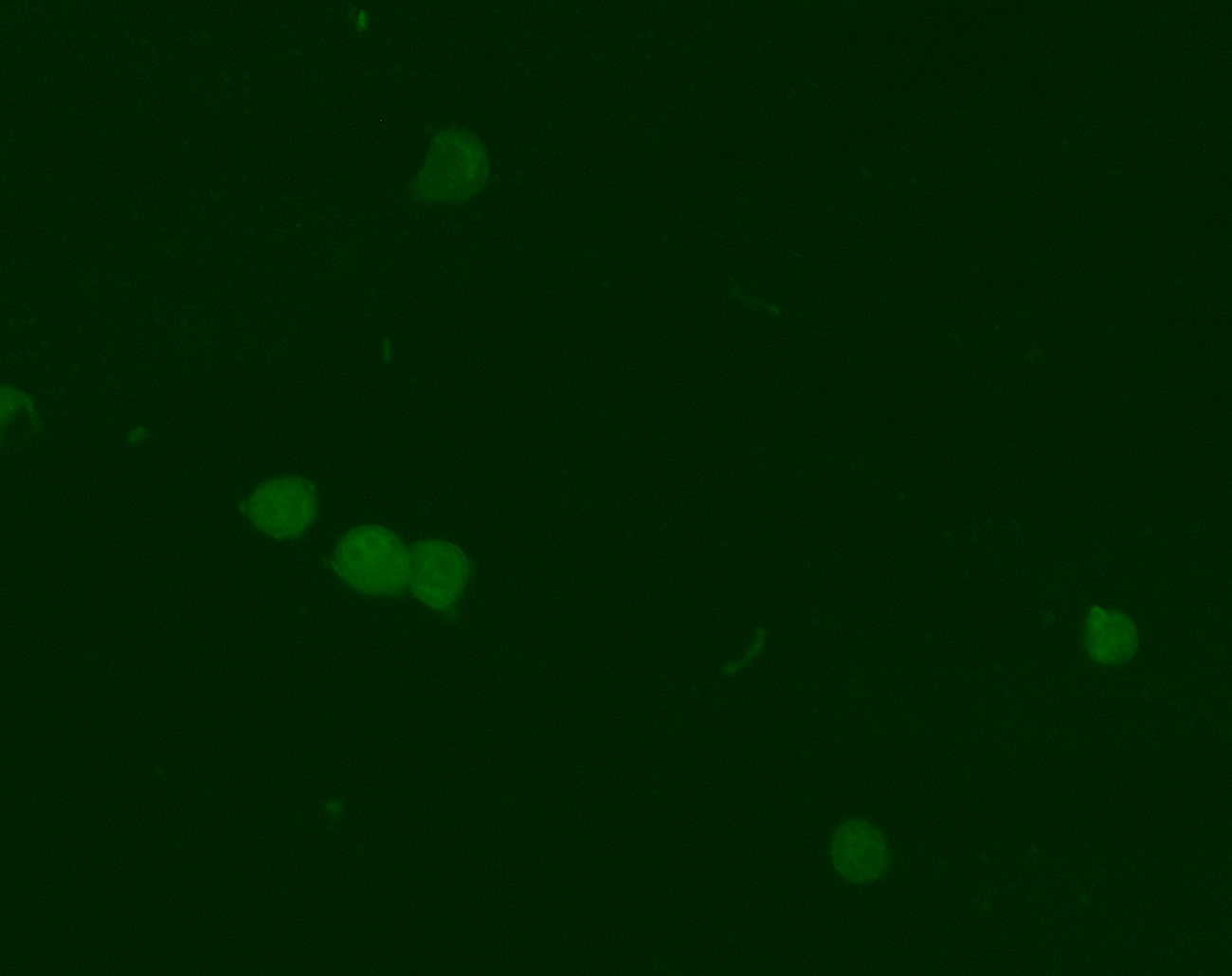

Supplement: Data S5 [file peerj-07-7725-s006.zip › Fig4_IF/03-1_FITC.jpg]

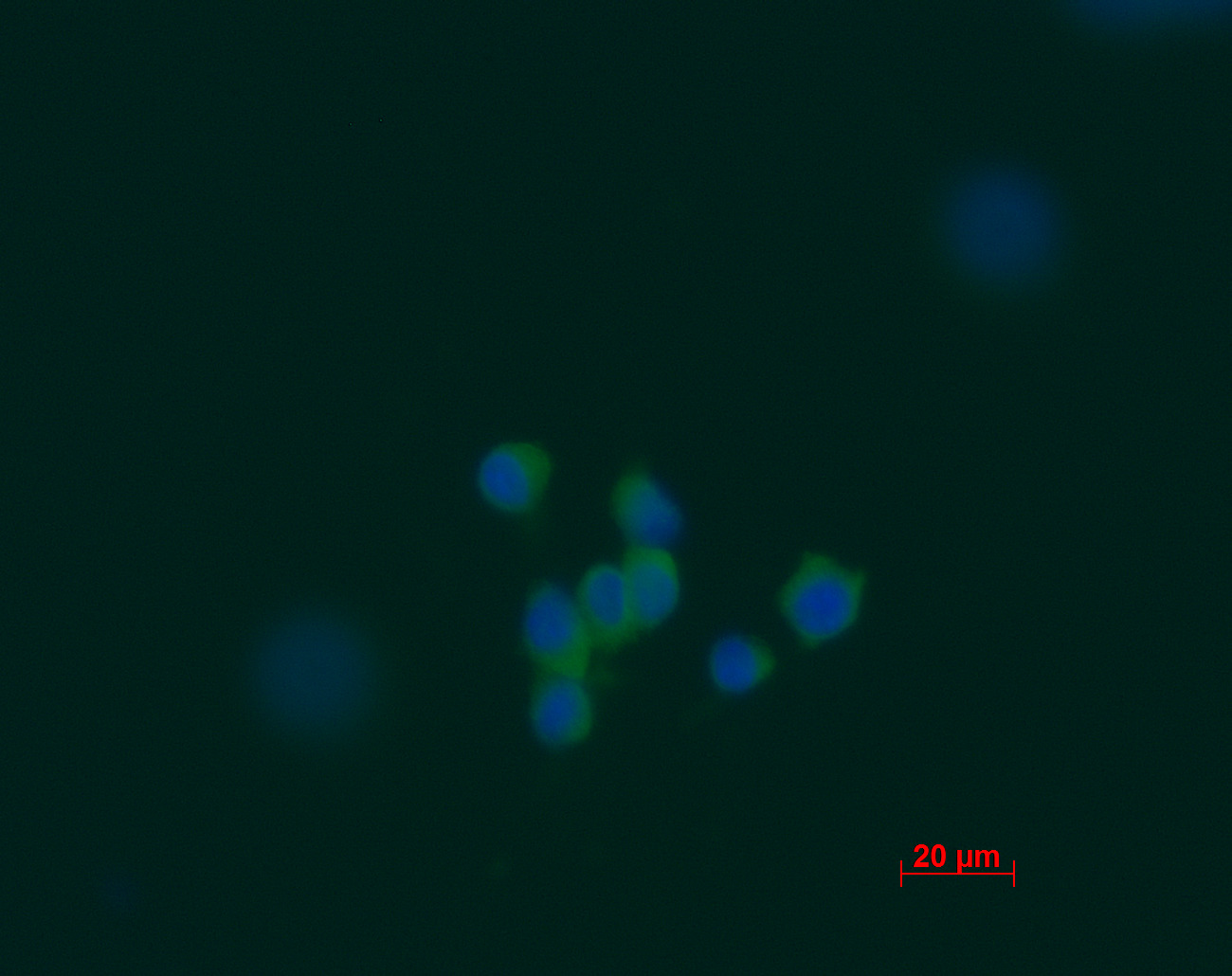

Supplement: Data S5 [file peerj-07-7725-s006.zip › Fig4_IF/01-1_(DAPI+FITC).jpg]

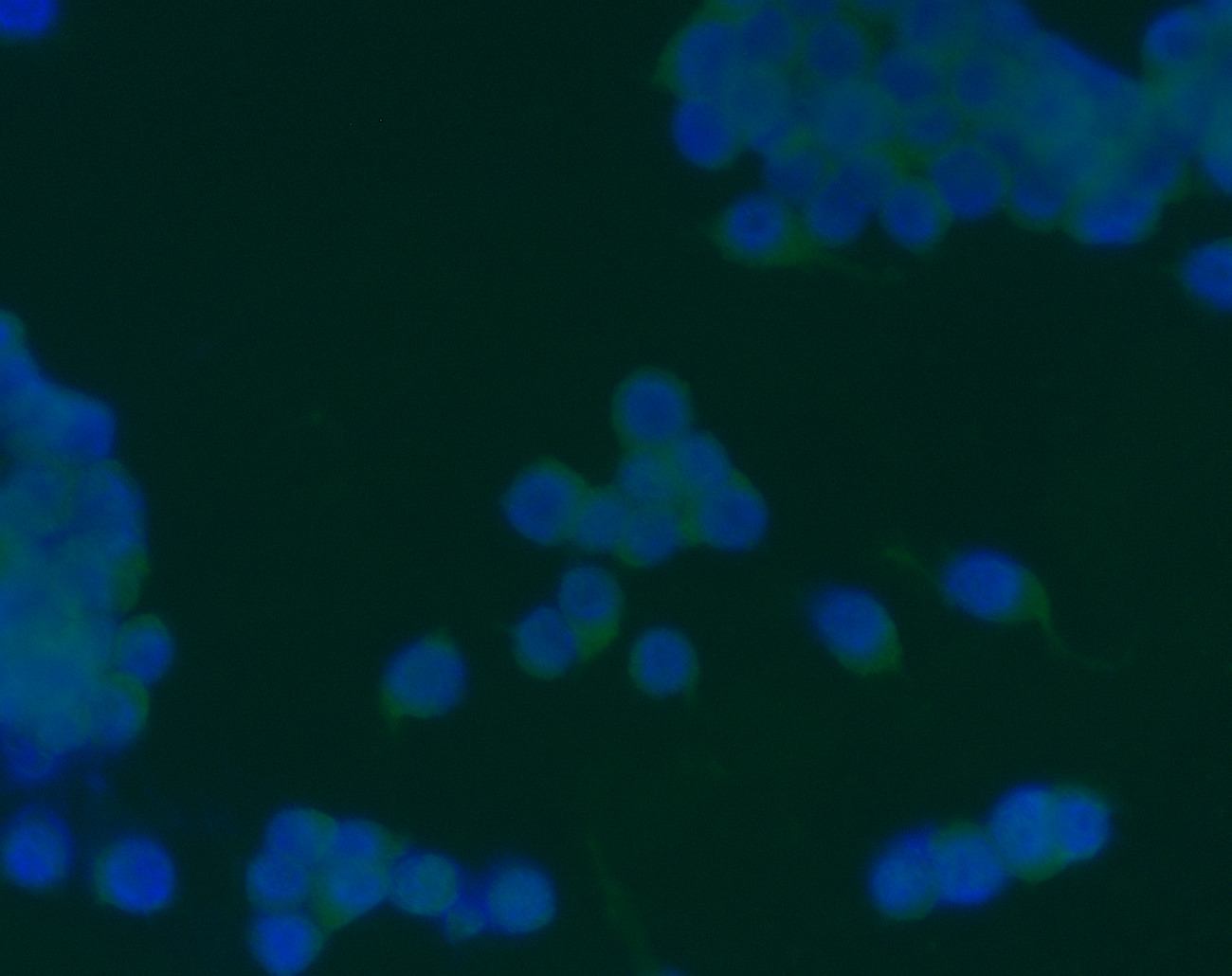

Supplement: Data S5 [file peerj-07-7725-s006.zip › Fig4_IF/05-1_(DAPI+FITC).jpg]

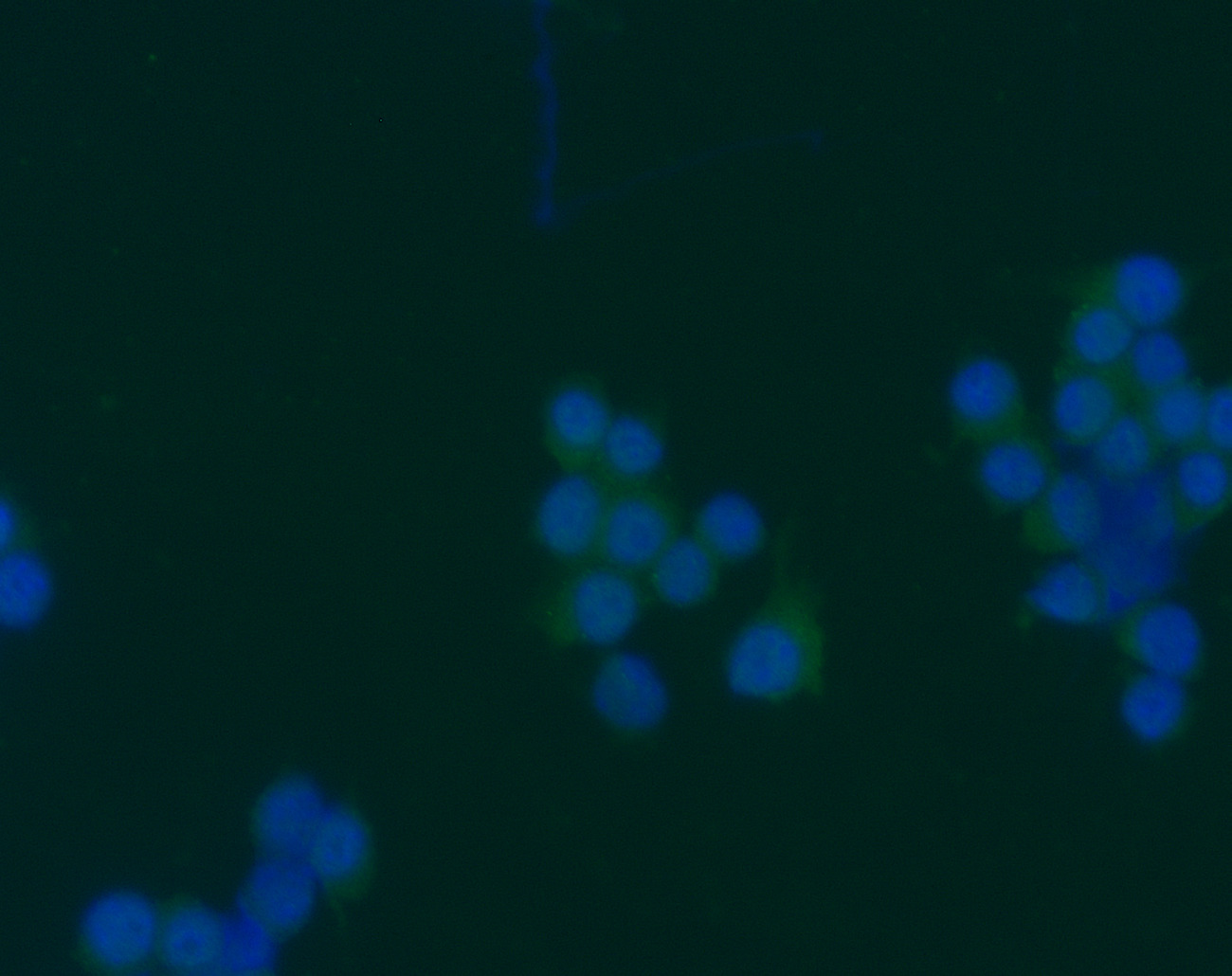

Supplement: Data S5 [file peerj-07-7725-s006.zip › Fig4_IF/04-2_(DAPI+FITC).jpg]

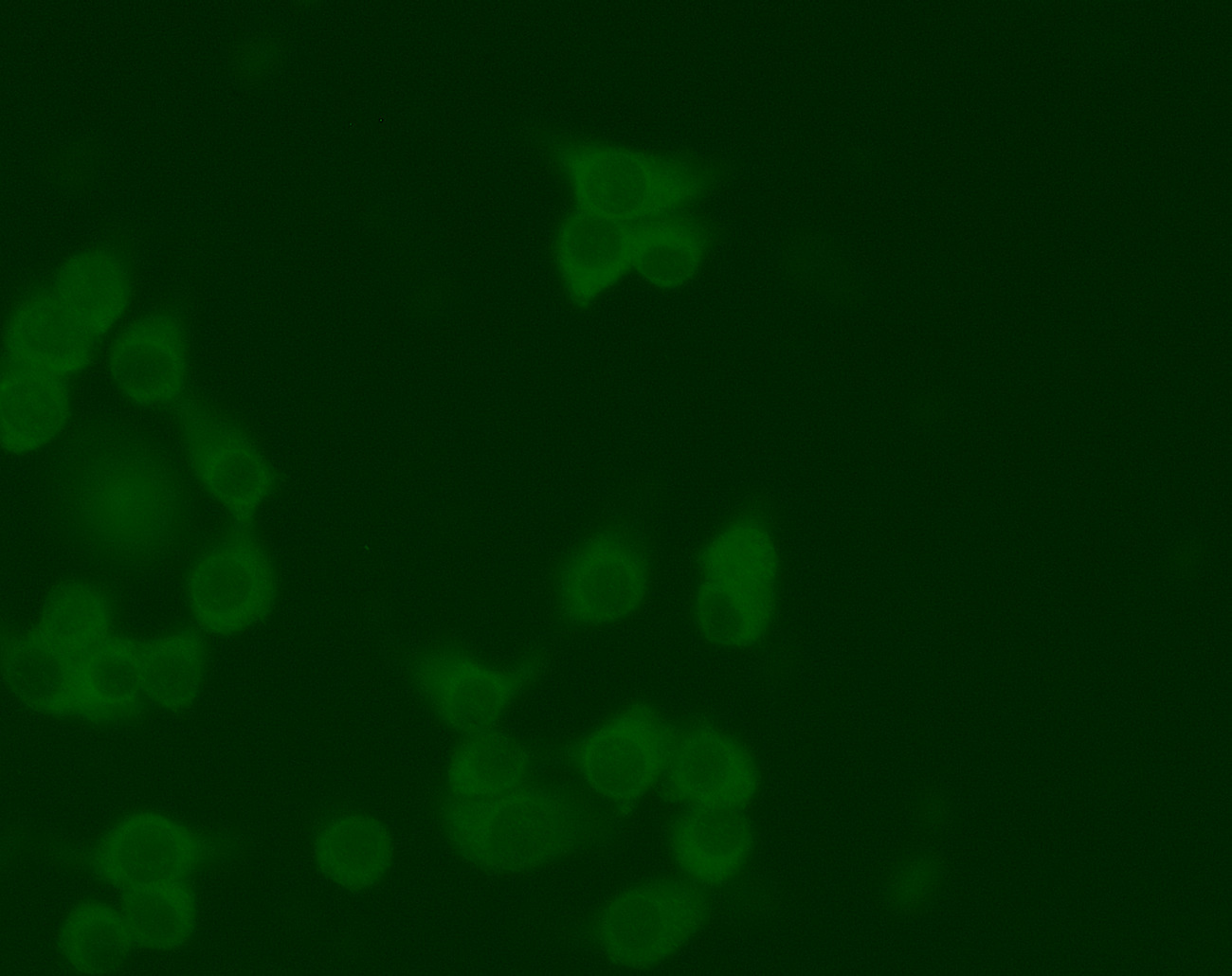

Supplement: Data S5 [file peerj-07-7725-s006.zip › Fig4_IF/01-2_FITC.jpg]

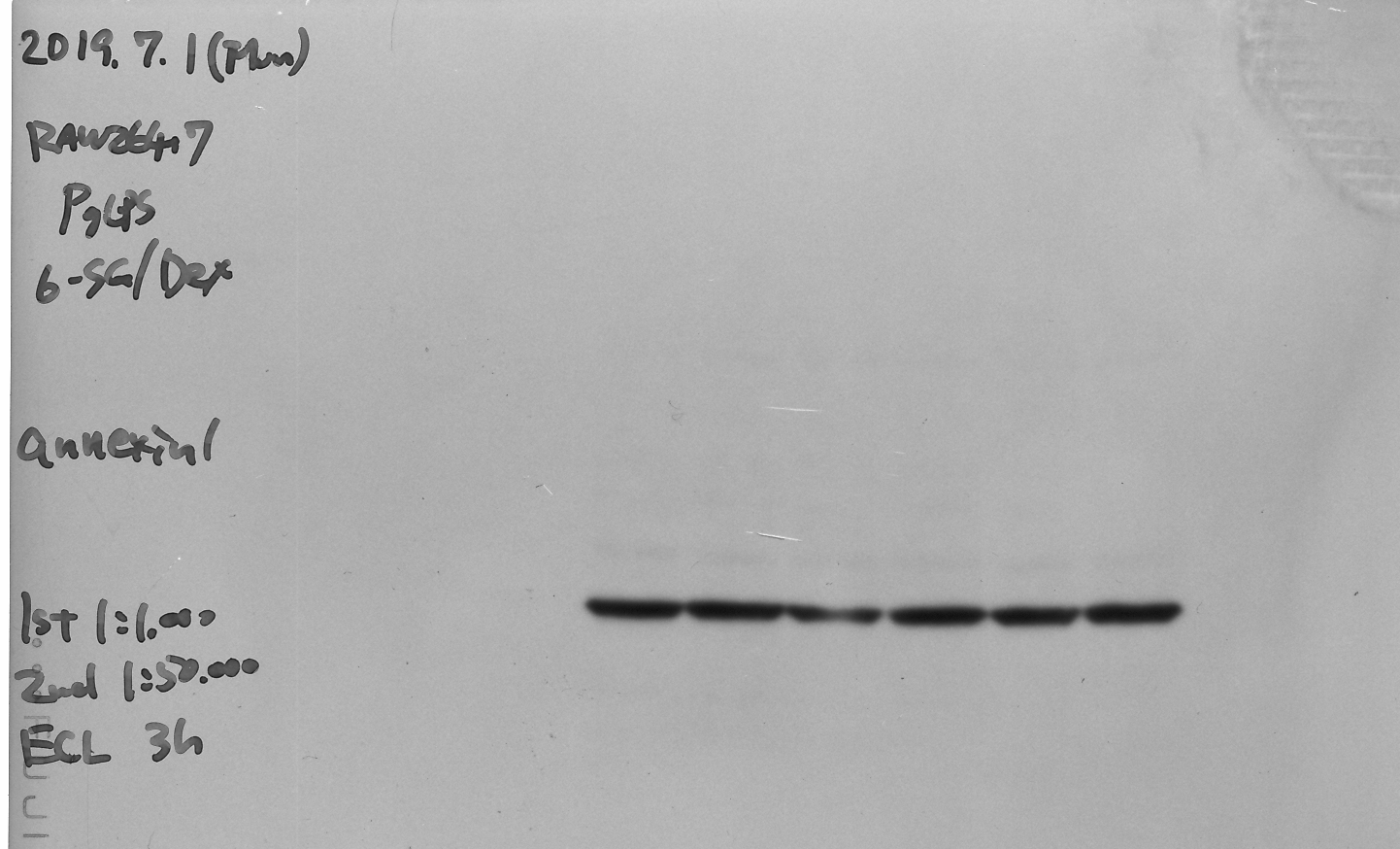

Supplement: Data S6 [file peerj-07-7725-s007.zip › Fig5/annexin1-20190701-1.png]

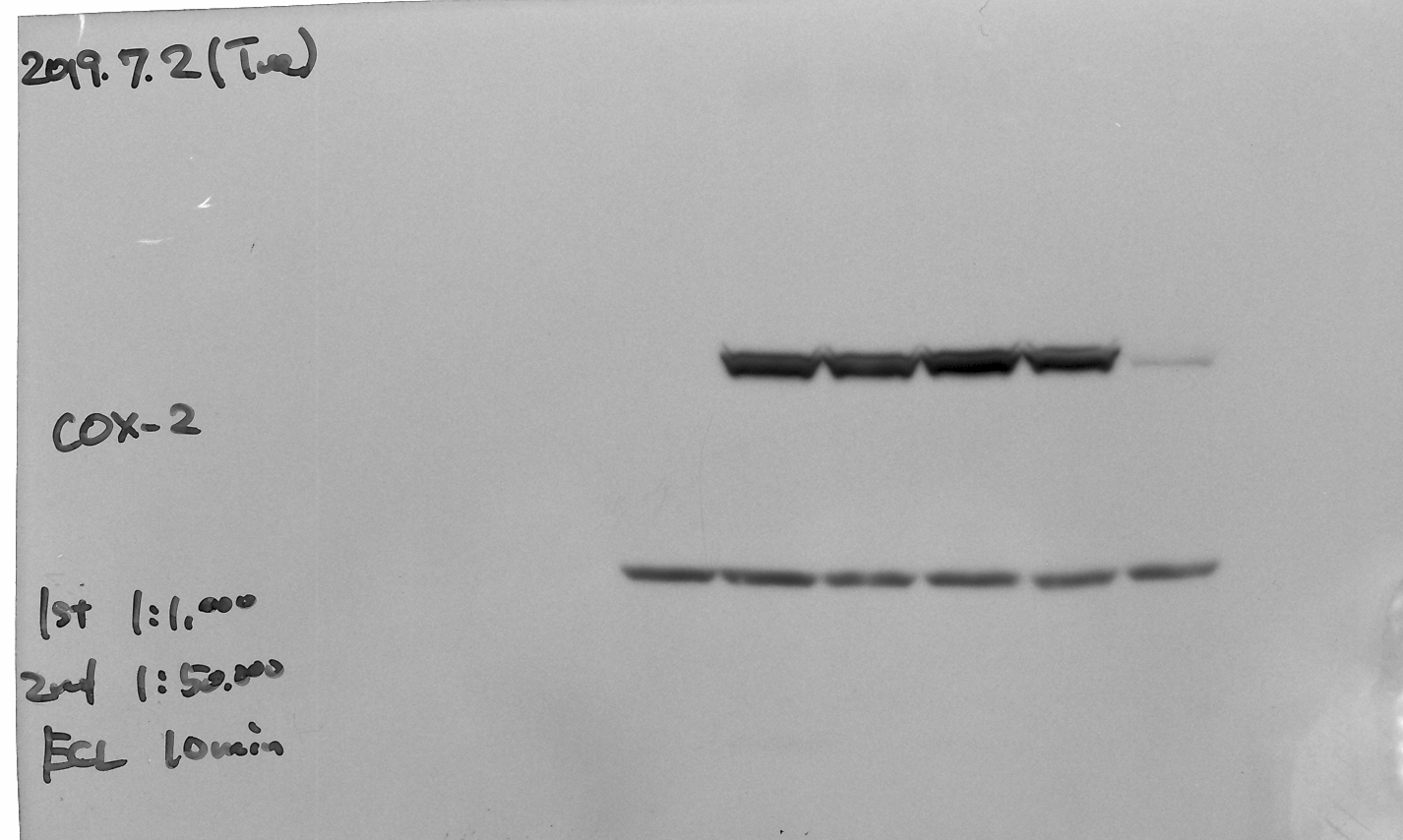

Supplement: Data S6 [file peerj-07-7725-s007.zip › Fig5/COX2-20190702-1.png]

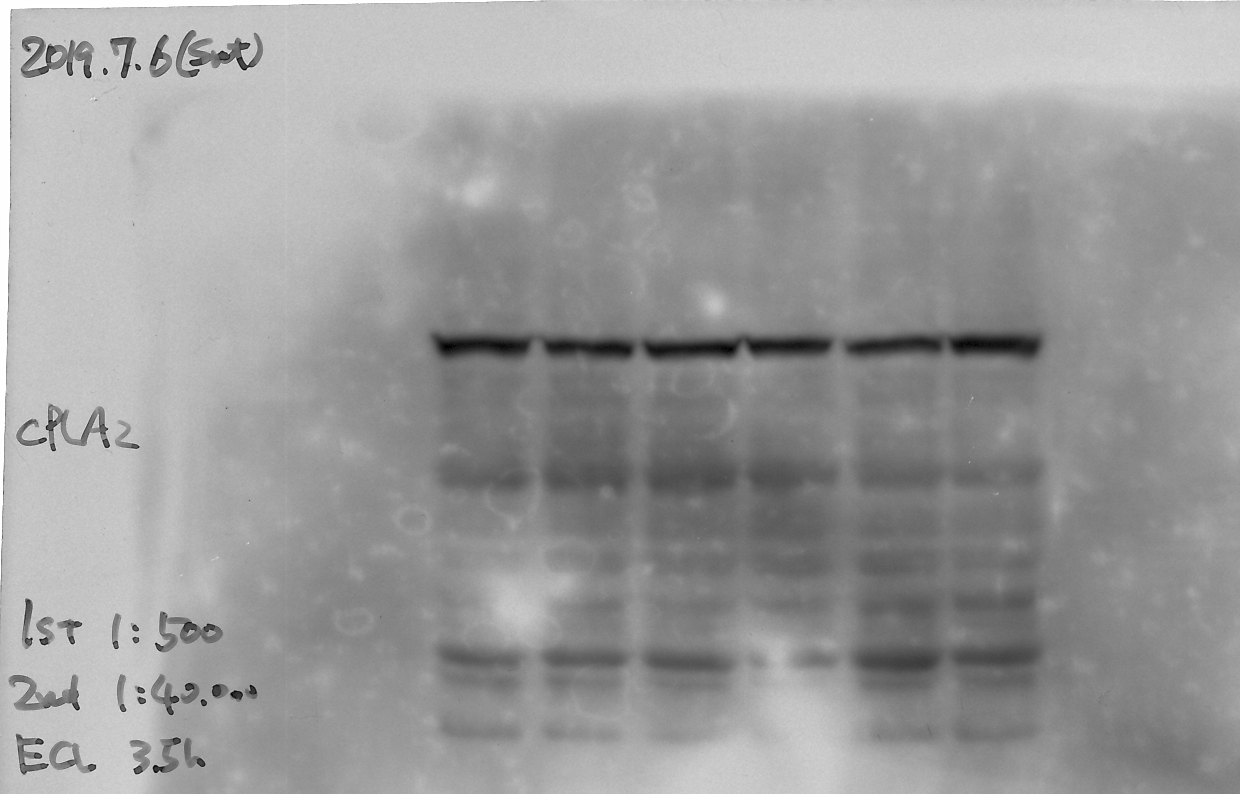

Supplement: Data S6 [file peerj-07-7725-s007.zip › Fig5/cPLA2-20190706-1.png]

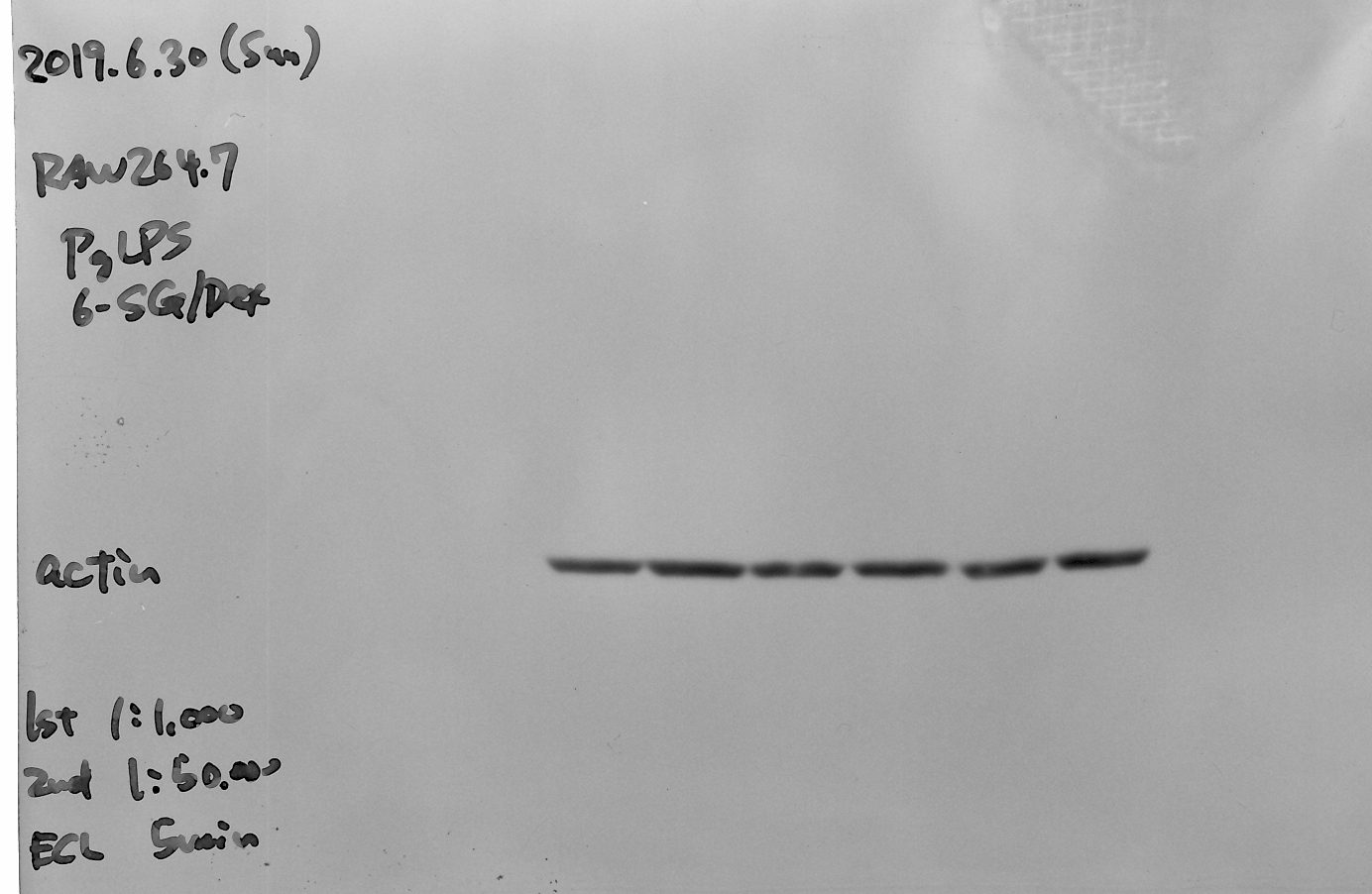

Supplement: Data S6 [file peerj-07-7725-s007.zip › Fig5/actin-20190630-1.png]

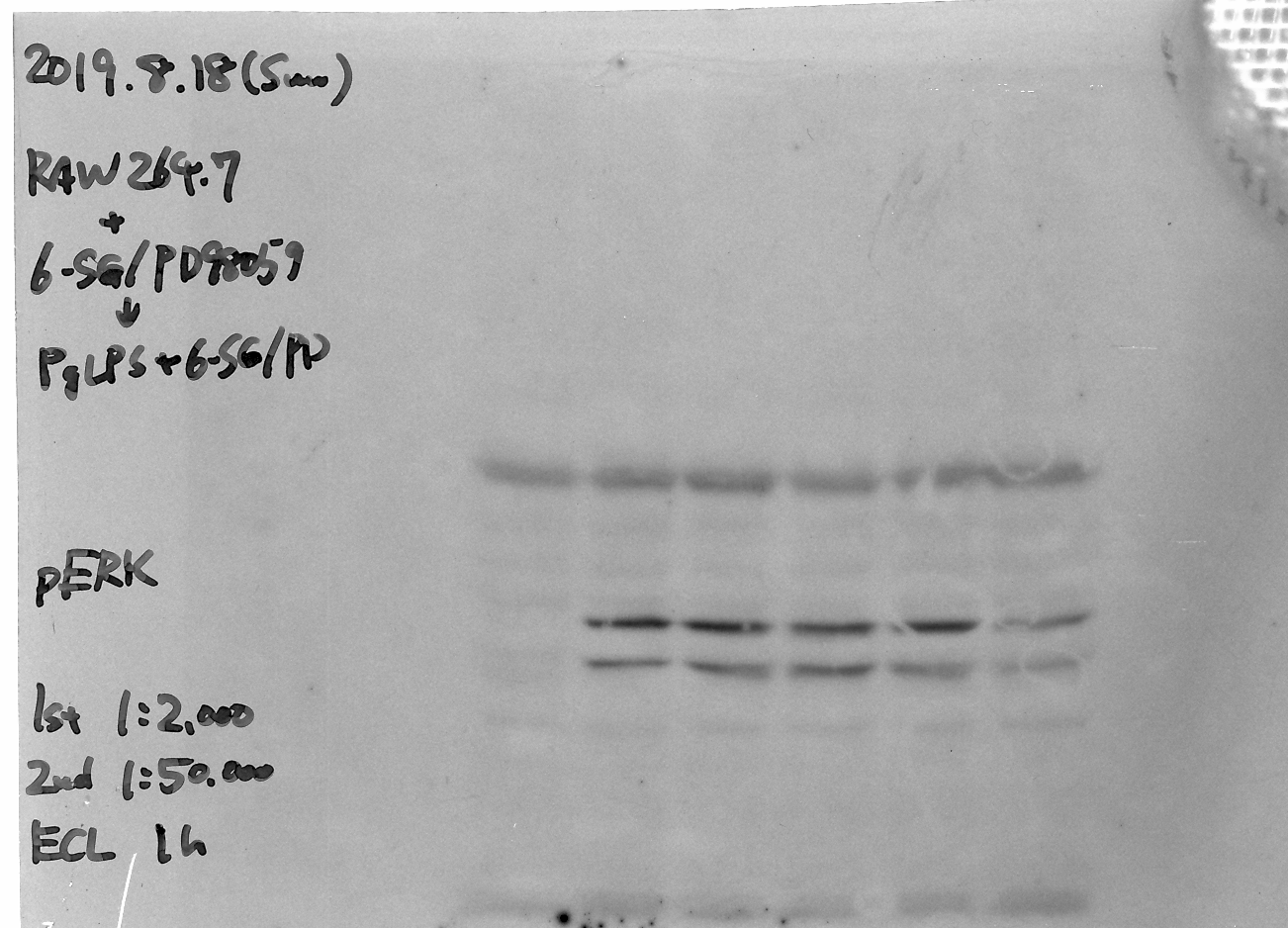

Supplement: Data S7 [file peerj-07-7725-s008.zip › Fig6/pERK-20190818-1.png]

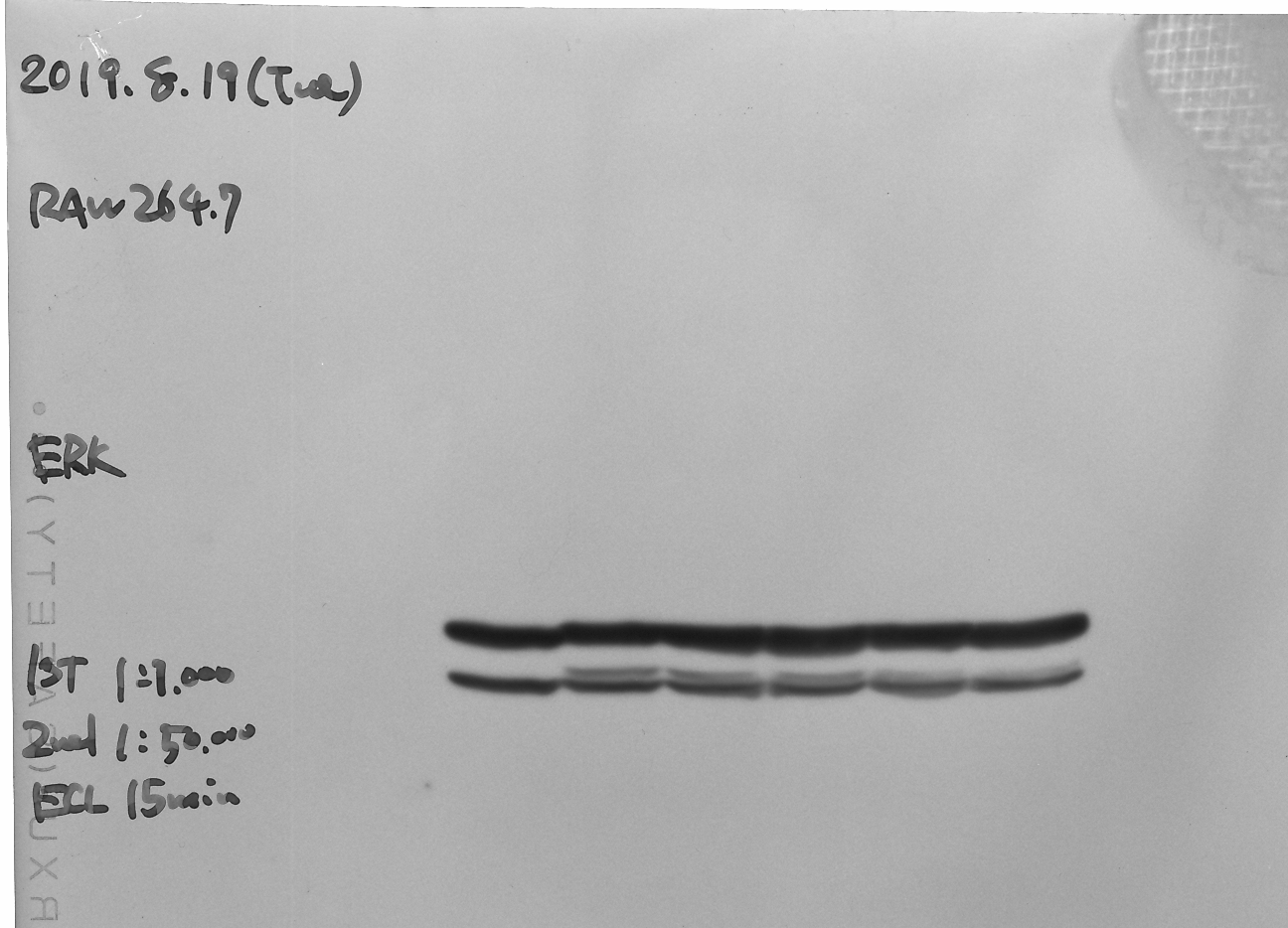

Supplement: Data S7 [file peerj-07-7725-s008.zip › Fig6/ERK-20190819-1.png]

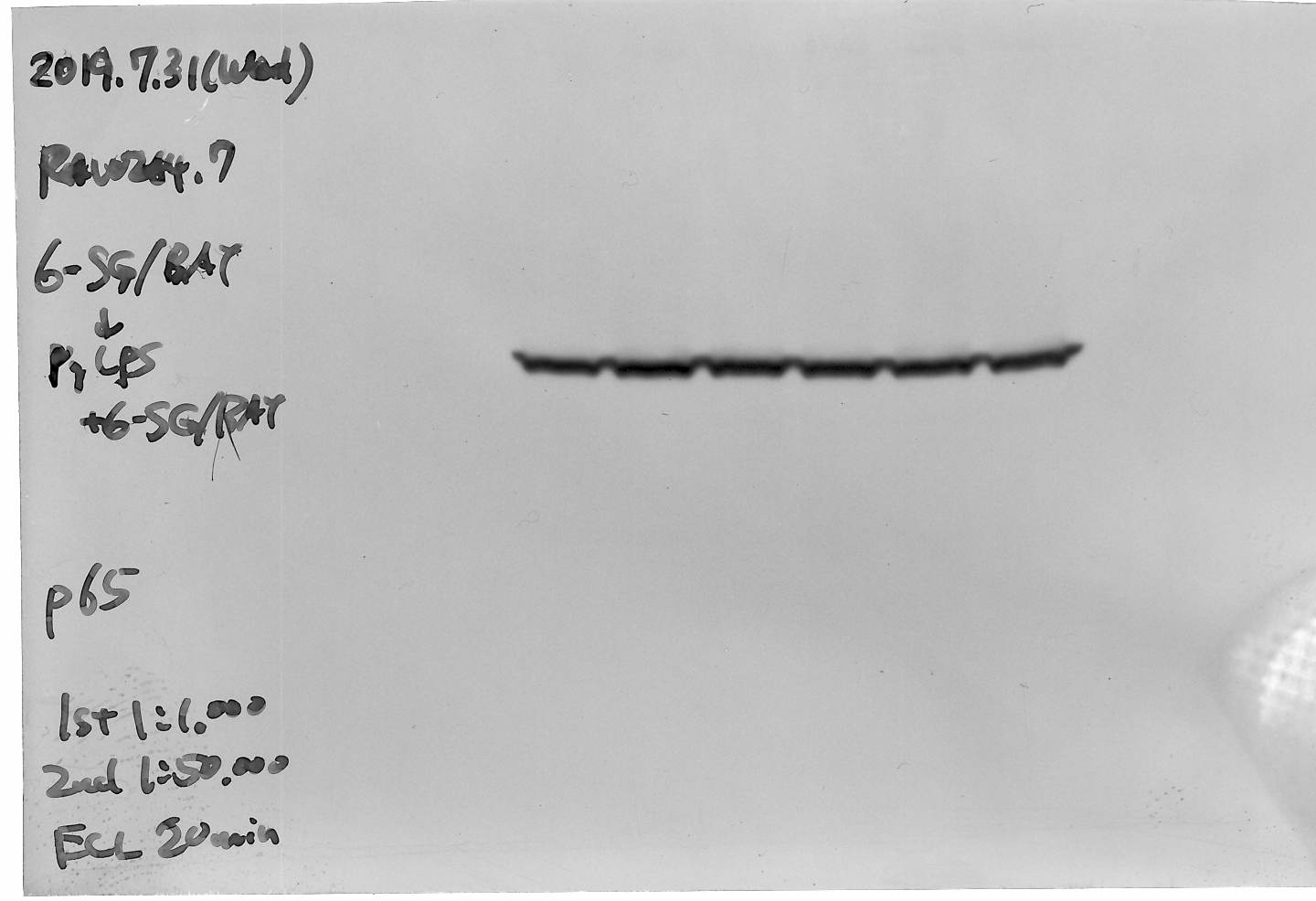

Supplement: Data S7 [file peerj-07-7725-s008.zip › Fig6/p65-20190730-1.png]

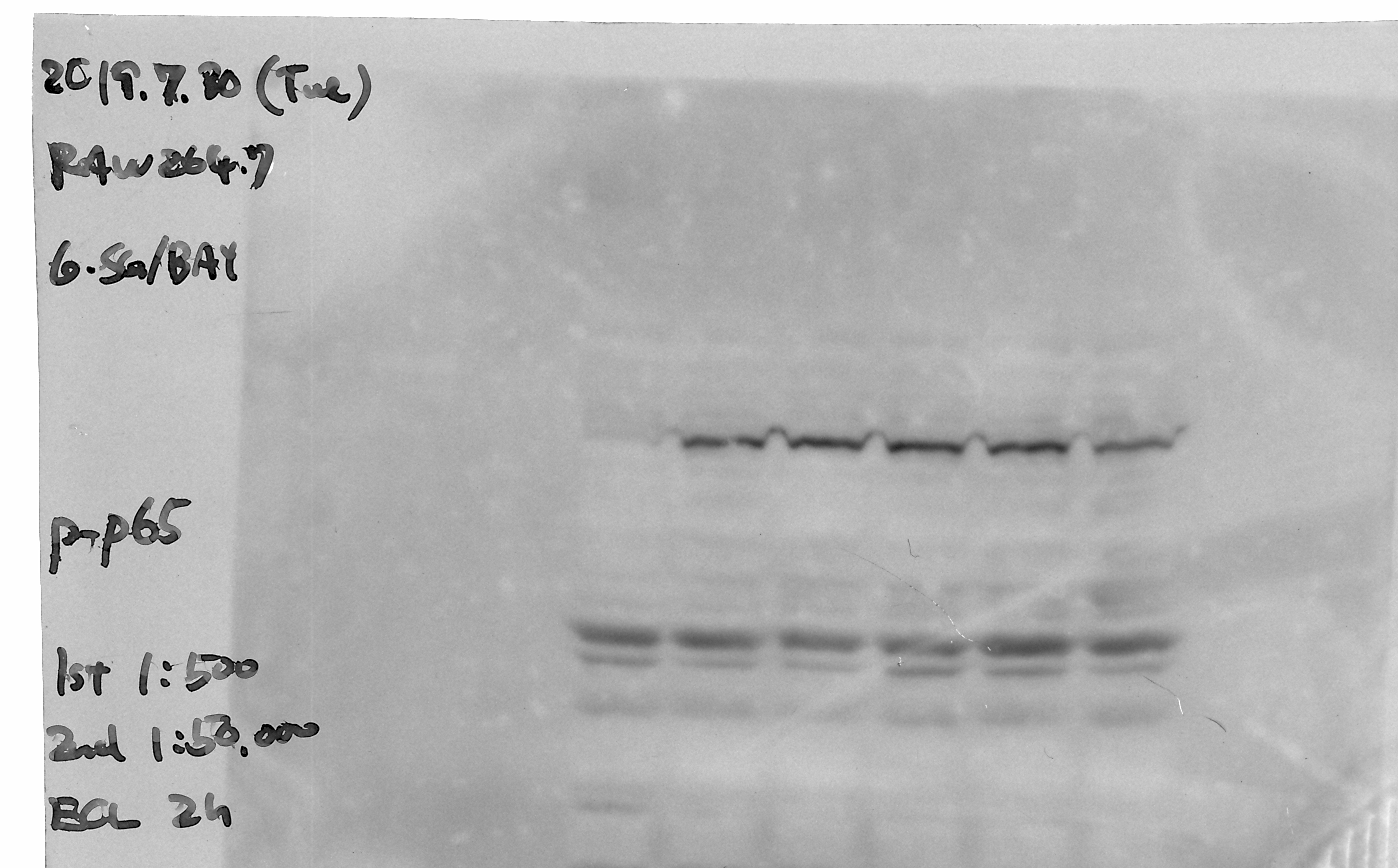

Supplement: Data S7 [file peerj-07-7725-s008.zip › Fig6/p-p65-20190730-1.png]

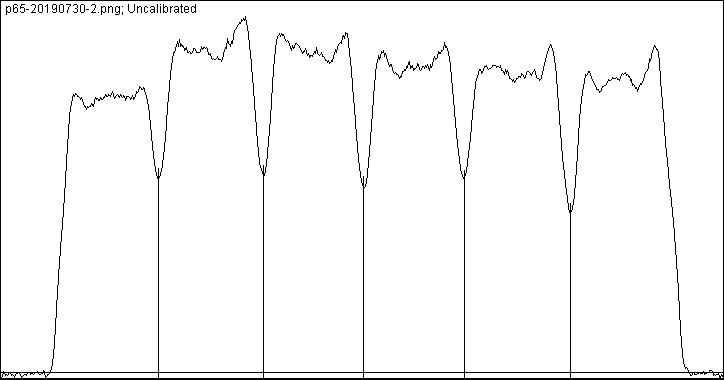

Supplement: Data S7 [file peerj-07-7725-s008.zip › analysis_ImageJ/Fig6B/p65.tif]

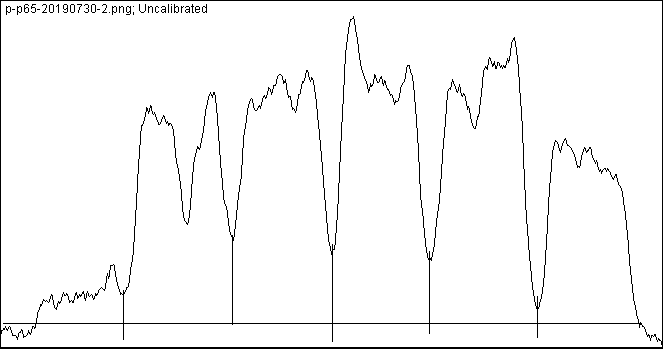

Supplement: Data S7 [file peerj-07-7725-s008.zip › analysis_ImageJ/Fig6B/p-p65.tif]

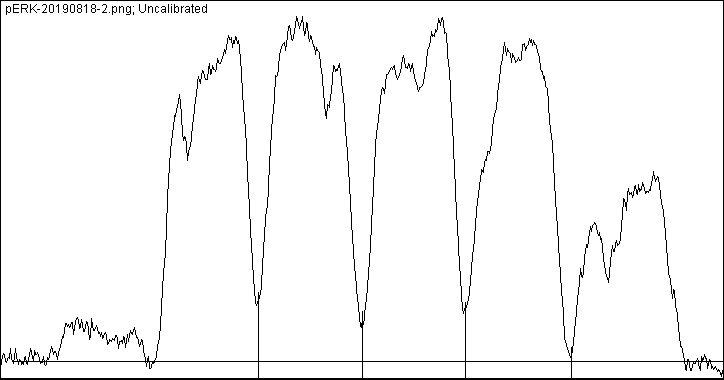

Supplement: Data S7 [file peerj-07-7725-s008.zip › analysis_ImageJ/Fig6C/pERK.tif]

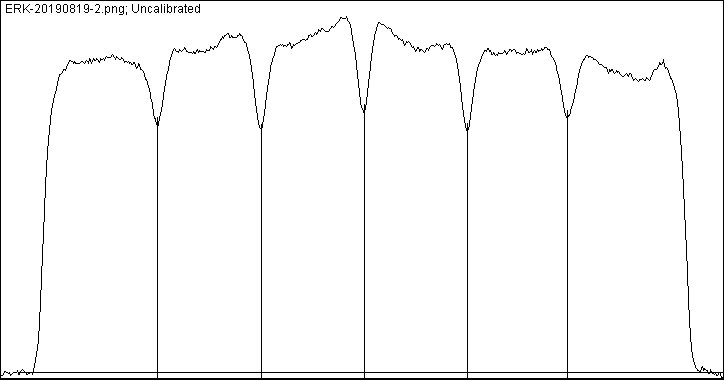

Supplement: Data S7 [file peerj-07-7725-s008.zip › analysis_ImageJ/Fig6C/ERK.tif]

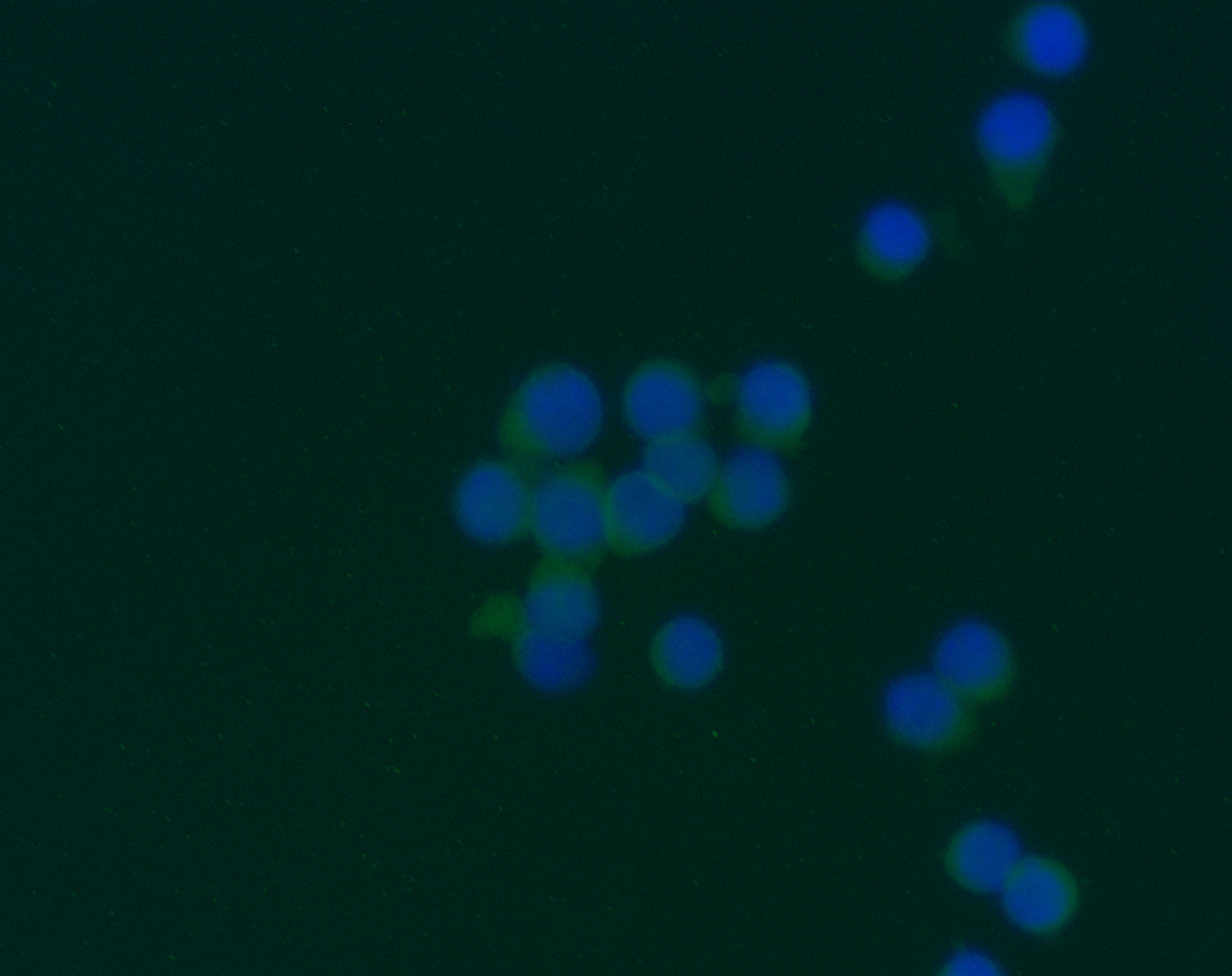

Supplement: Data S8 [file peerj-07-7725-s009.zip › Fig6_IF/11-1_(DAPI+FITC).jpg]

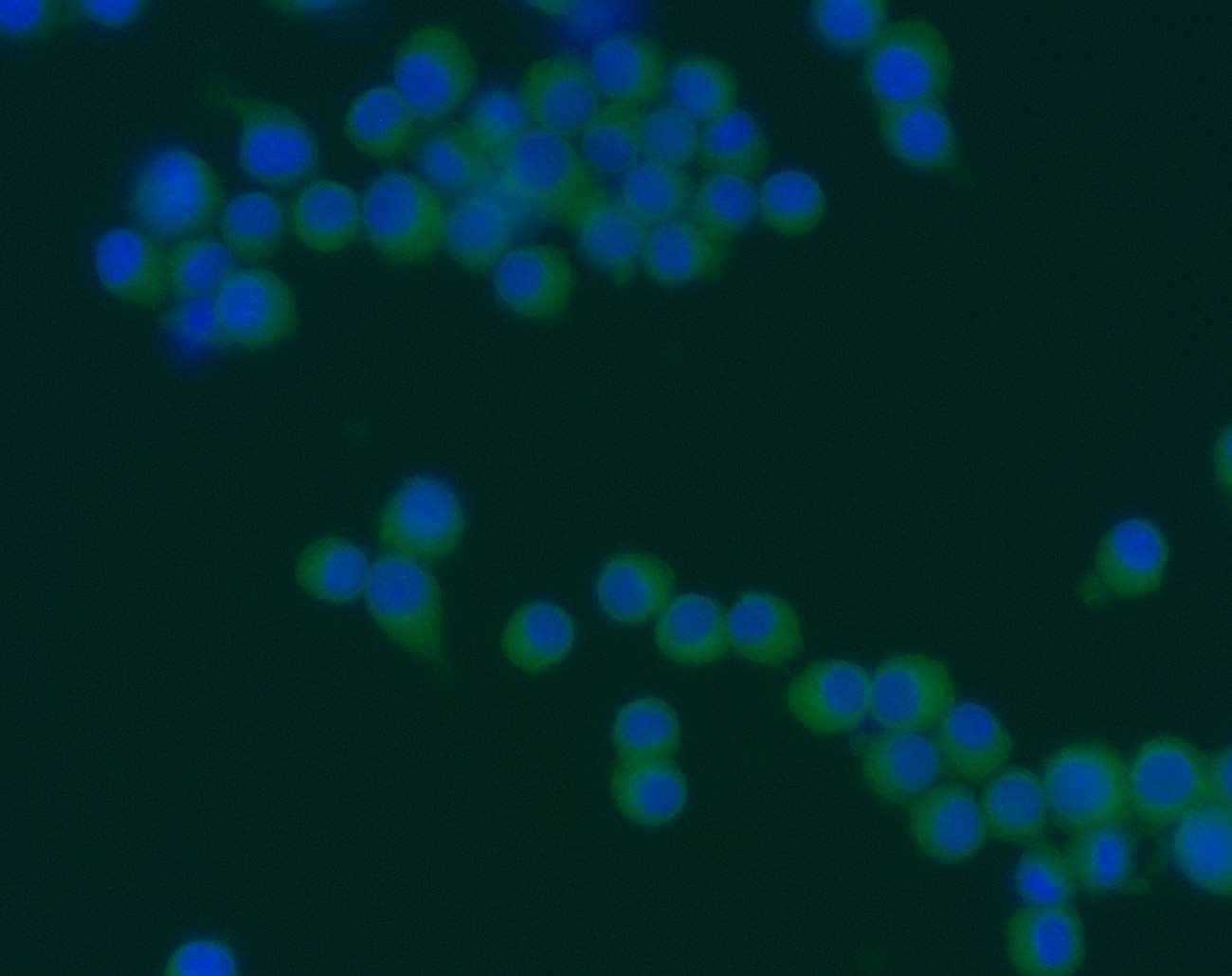

Supplement: Data S8 [file peerj-07-7725-s009.zip › Fig6_IF/06-1_(DAPI+FITC).jpg]

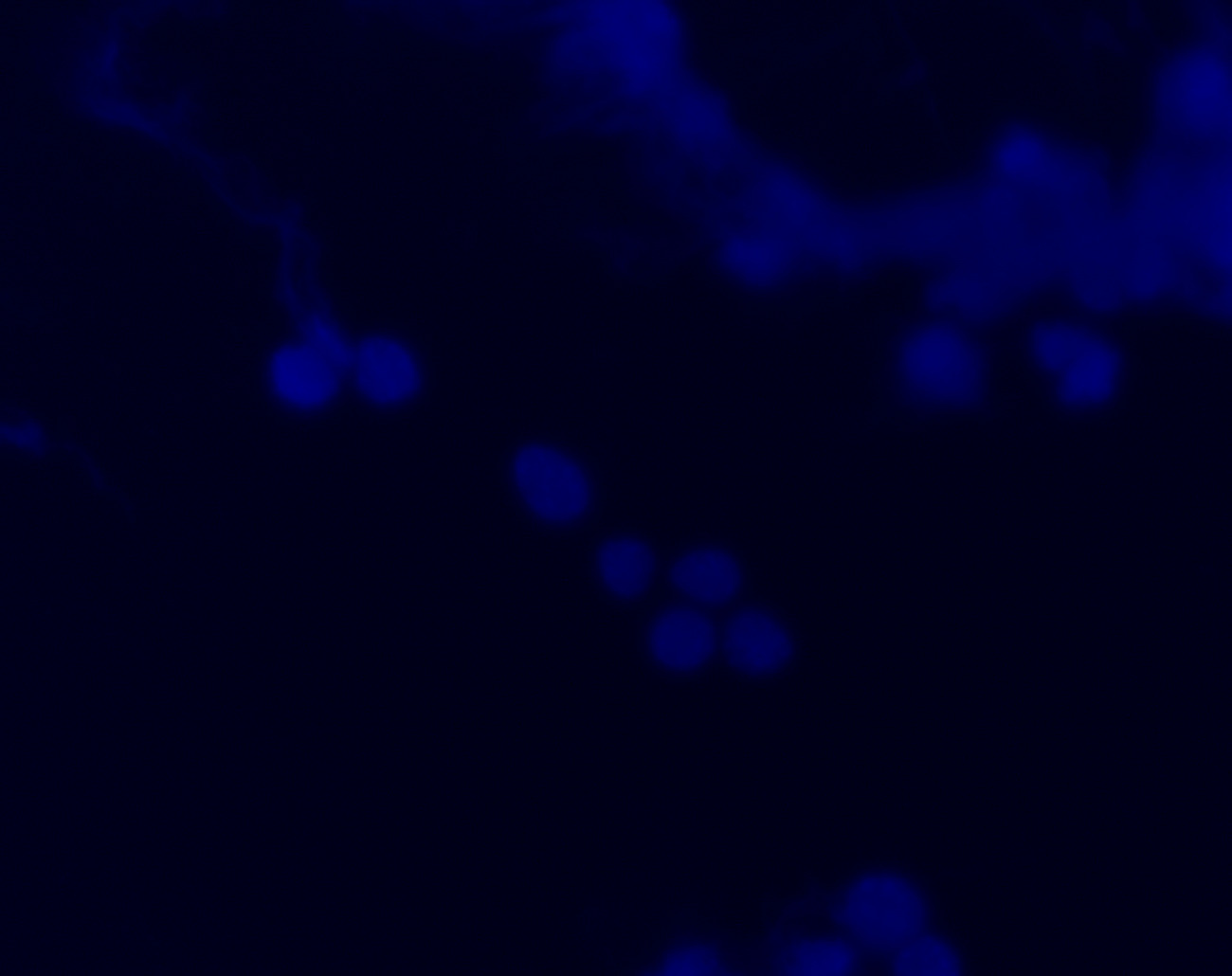

Supplement: Data S8 [file peerj-07-7725-s009.zip › Fig6_IF/07-2_DAPI.jpg]

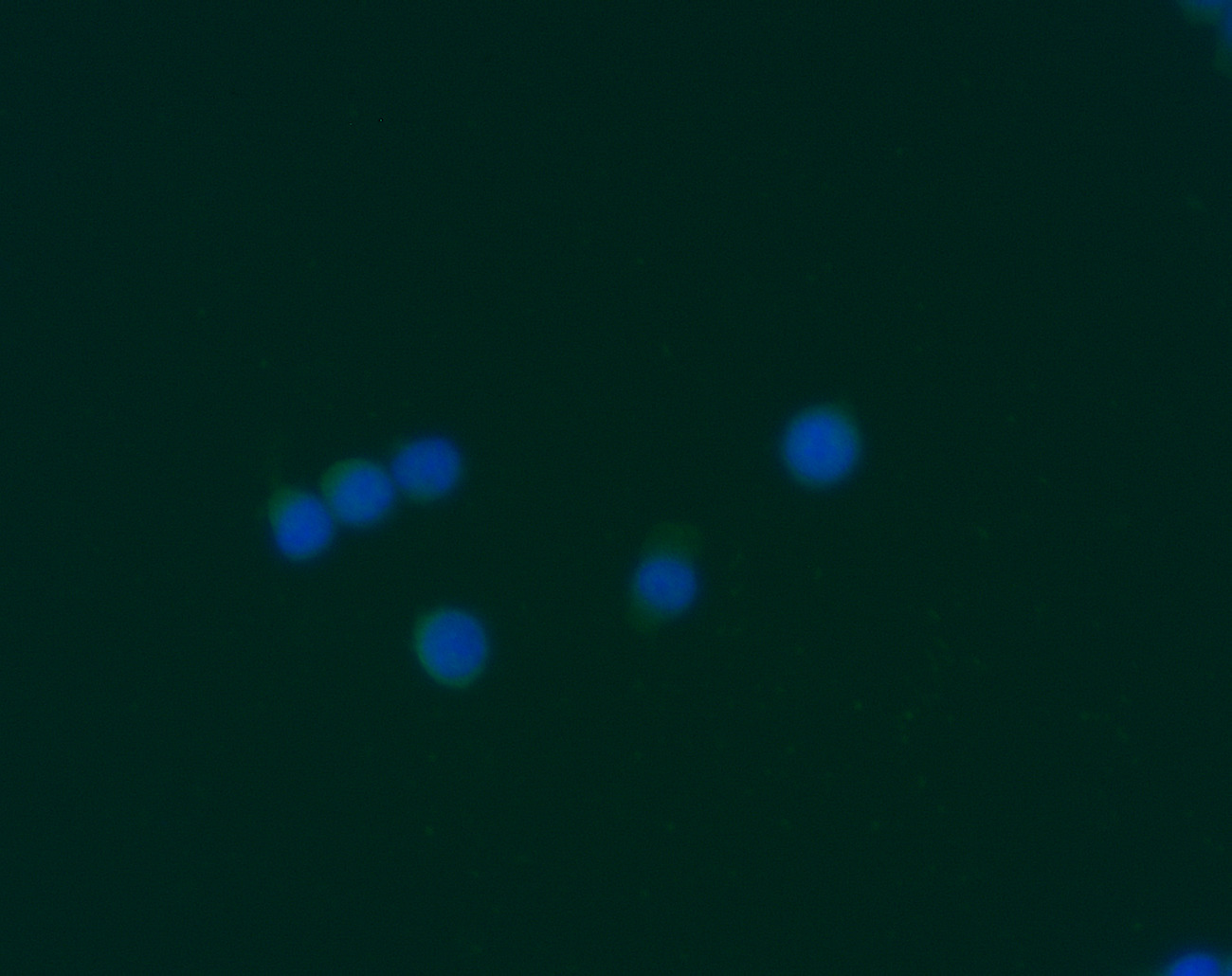

Supplement: Data S8 [file peerj-07-7725-s009.zip › Fig6_IF/09-2_(DAPI+FITC).jpg]

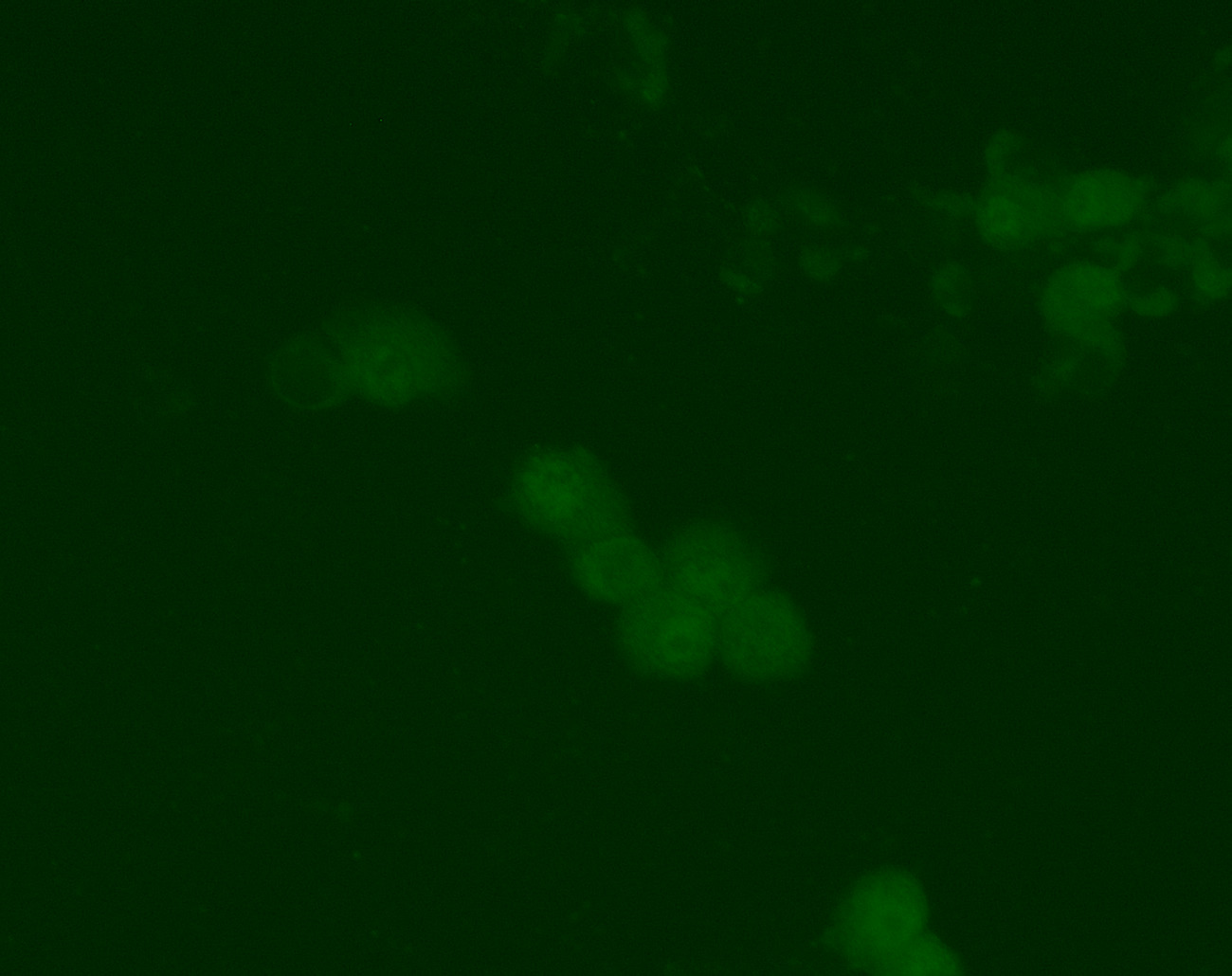

Supplement: Data S8 [file peerj-07-7725-s009.zip › Fig6_IF/07-2_FITC.jpg]

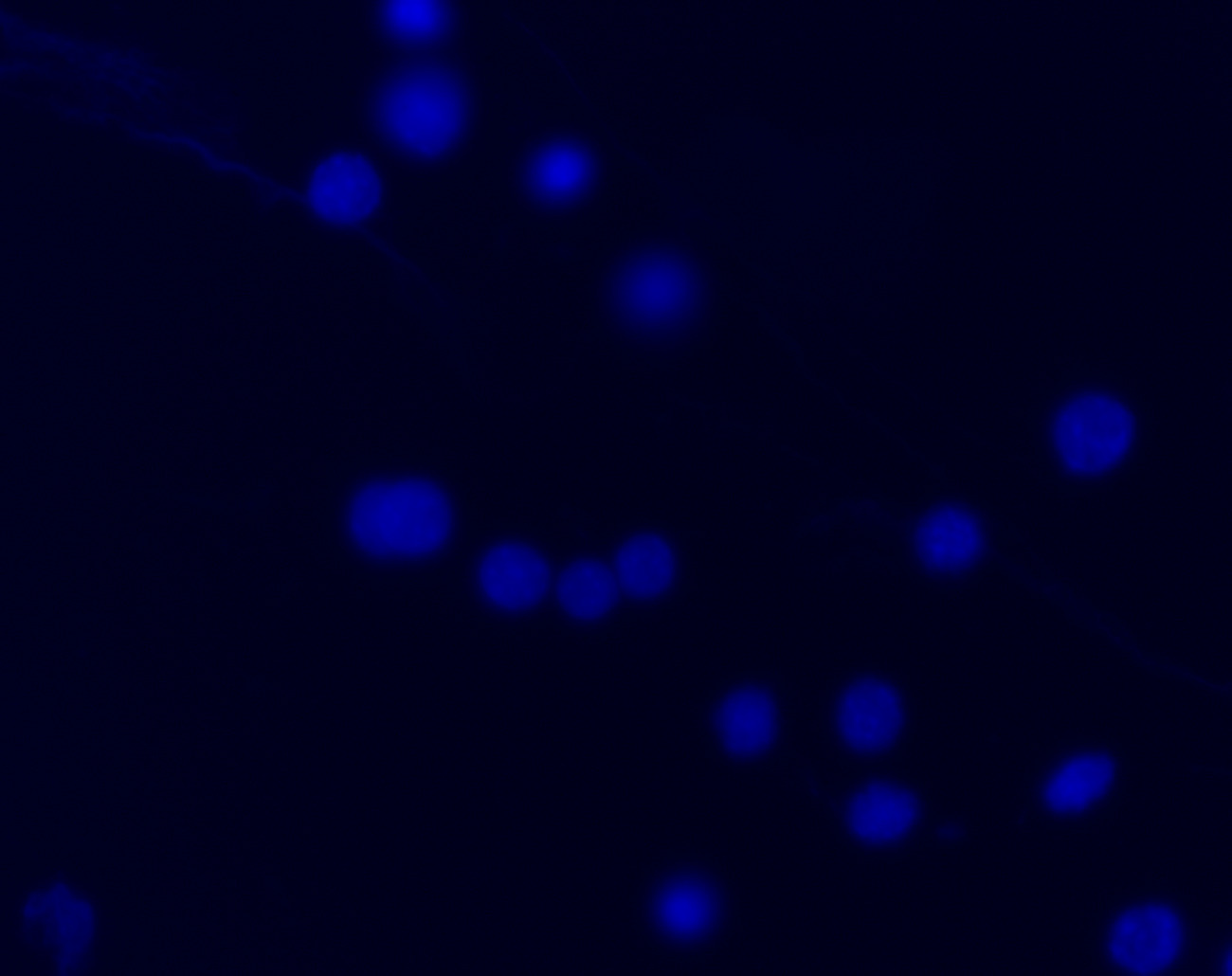

Supplement: Data S8 [file peerj-07-7725-s009.zip › Fig6_IF/10-2_DAPI.jpg]

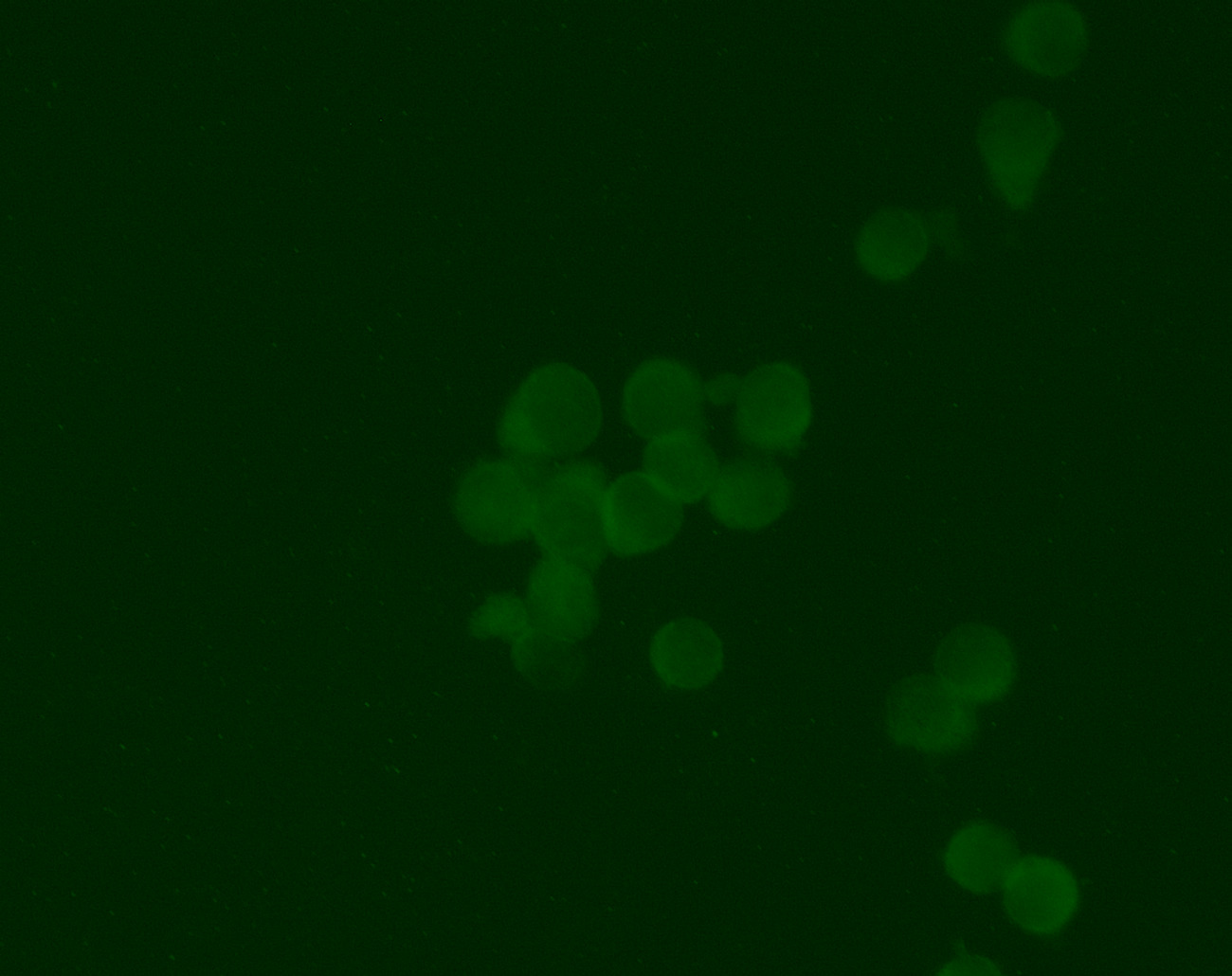

Supplement: Data S8 [file peerj-07-7725-s009.zip › Fig6_IF/11-1_FITC.jpg]

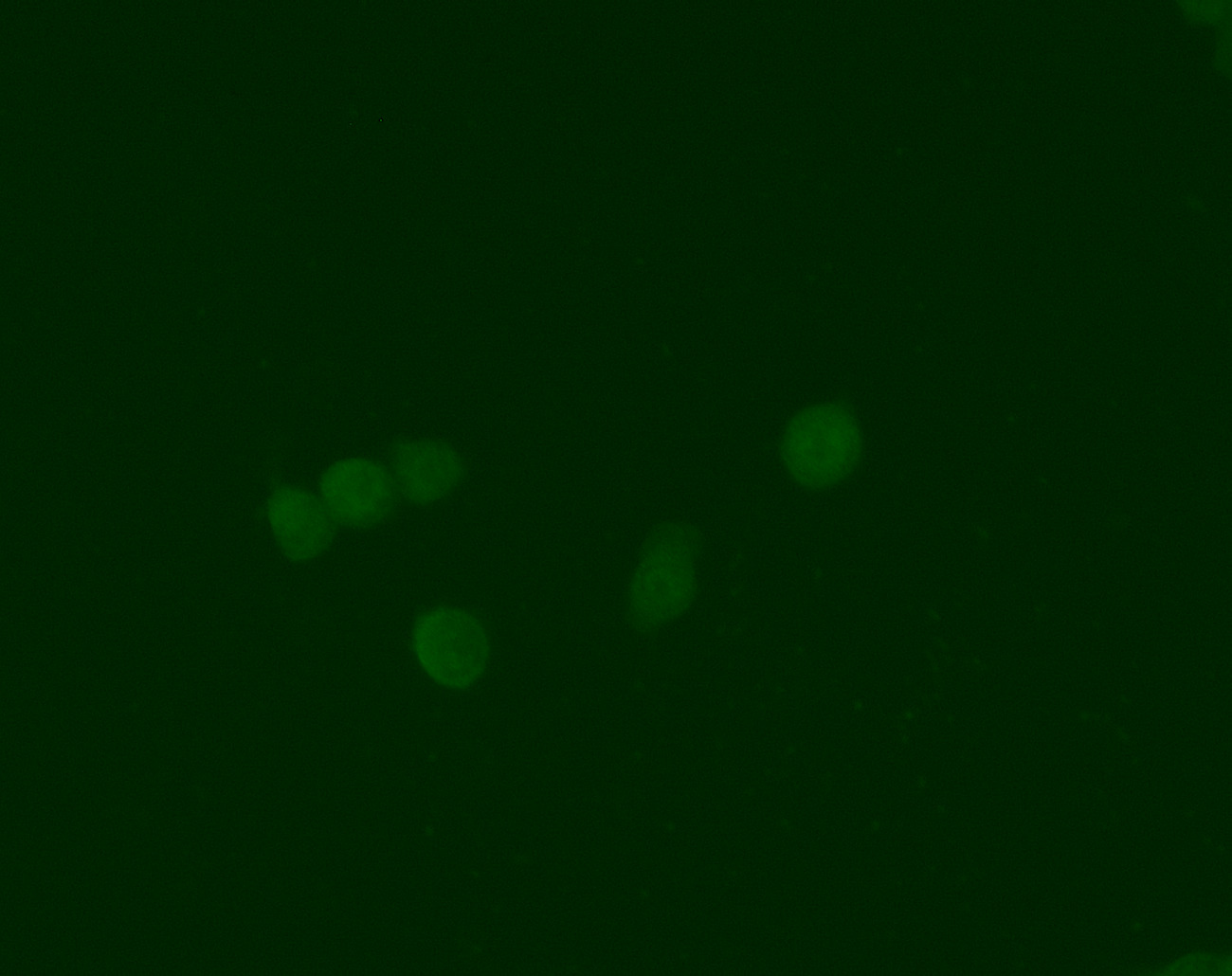

Supplement: Data S8 [file peerj-07-7725-s009.zip › Fig6_IF/09-2_FITC.jpg]

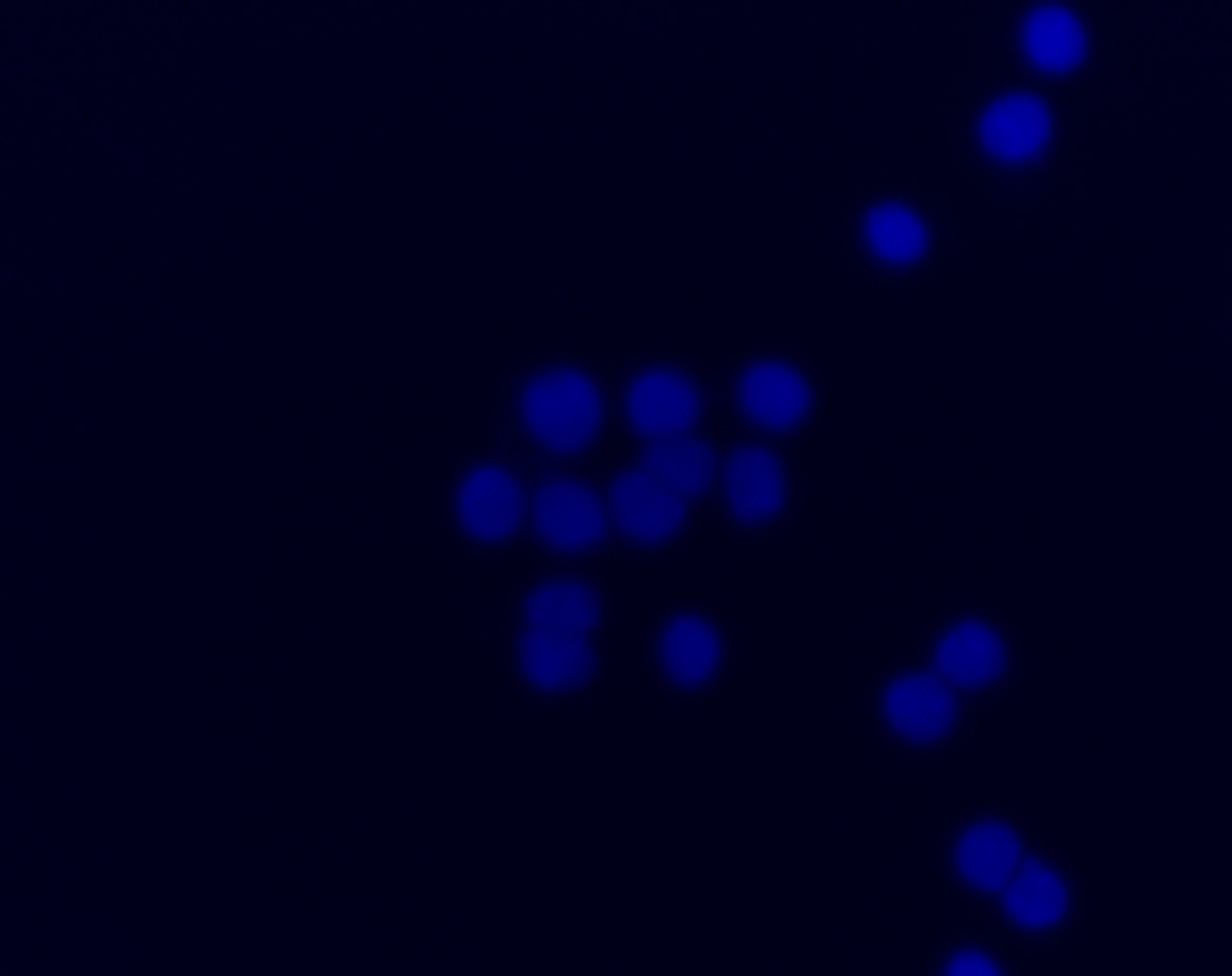

Supplement: Data S8 [file peerj-07-7725-s009.zip › Fig6_IF/11-1_DAPI.jpg]

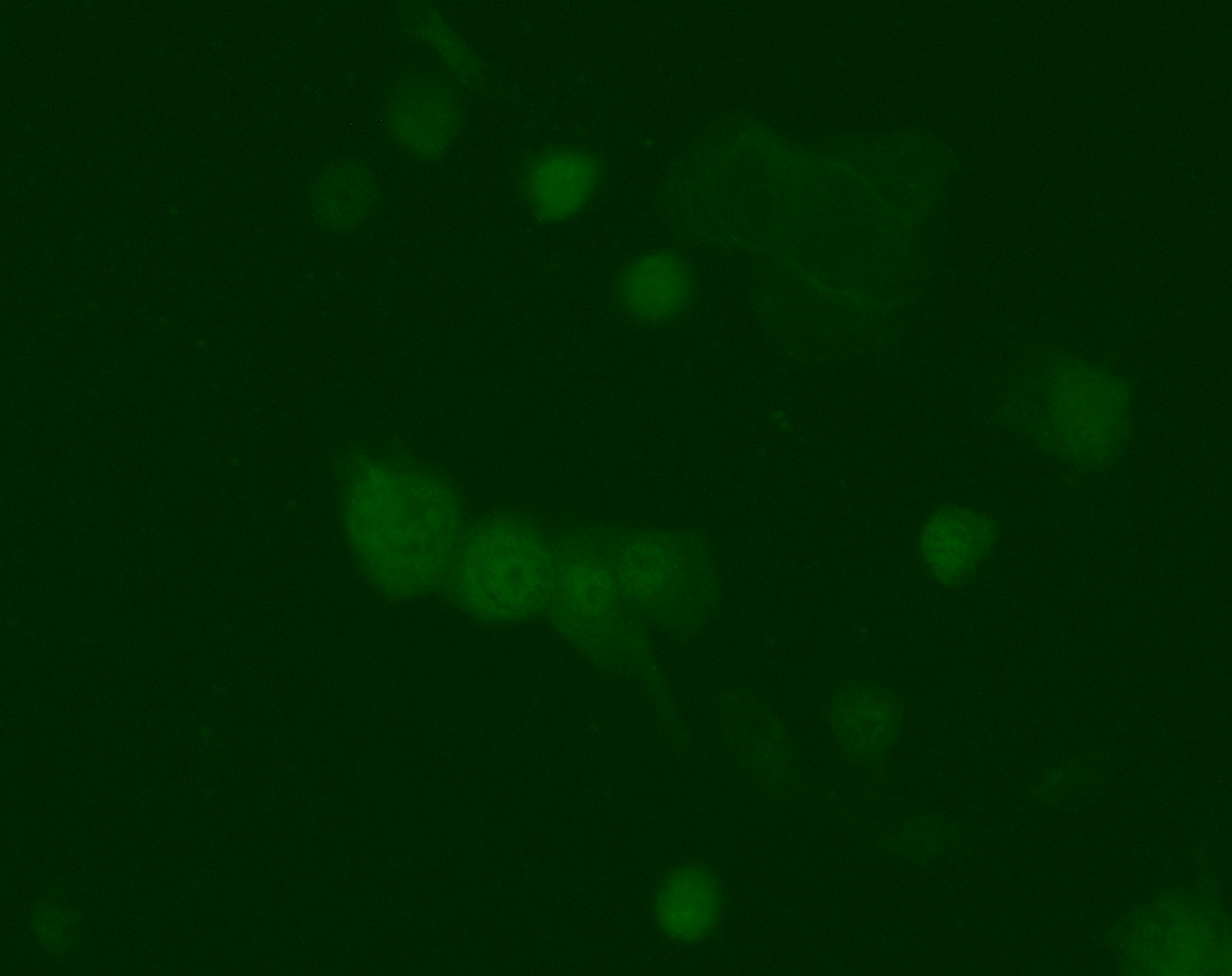

Supplement: Data S8 [file peerj-07-7725-s009.zip › Fig6_IF/10-2_FITC.jpg]

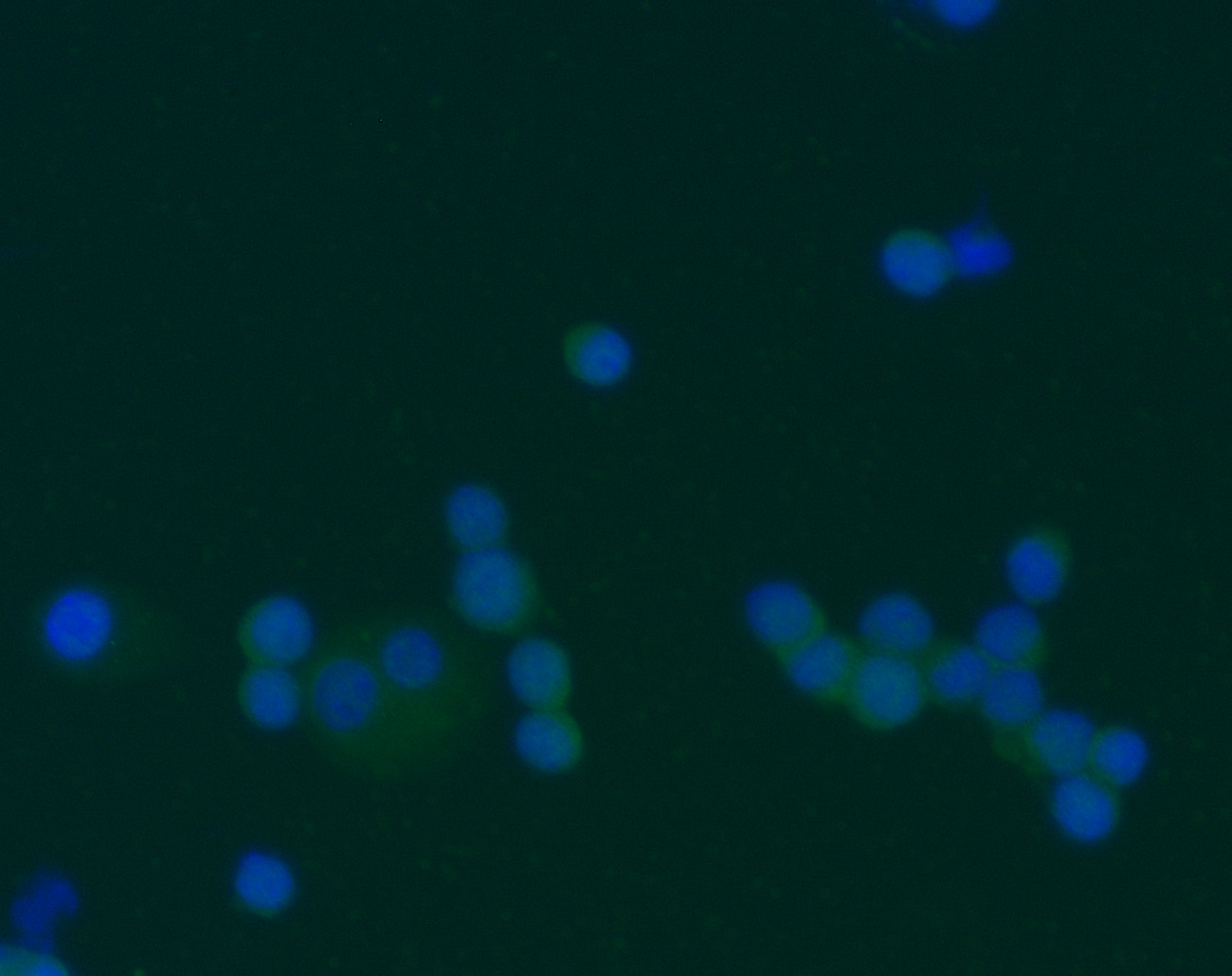

Supplement: Data S8 [file peerj-07-7725-s009.zip › Fig6_IF/08-2_(DAPI+FITC).jpg]

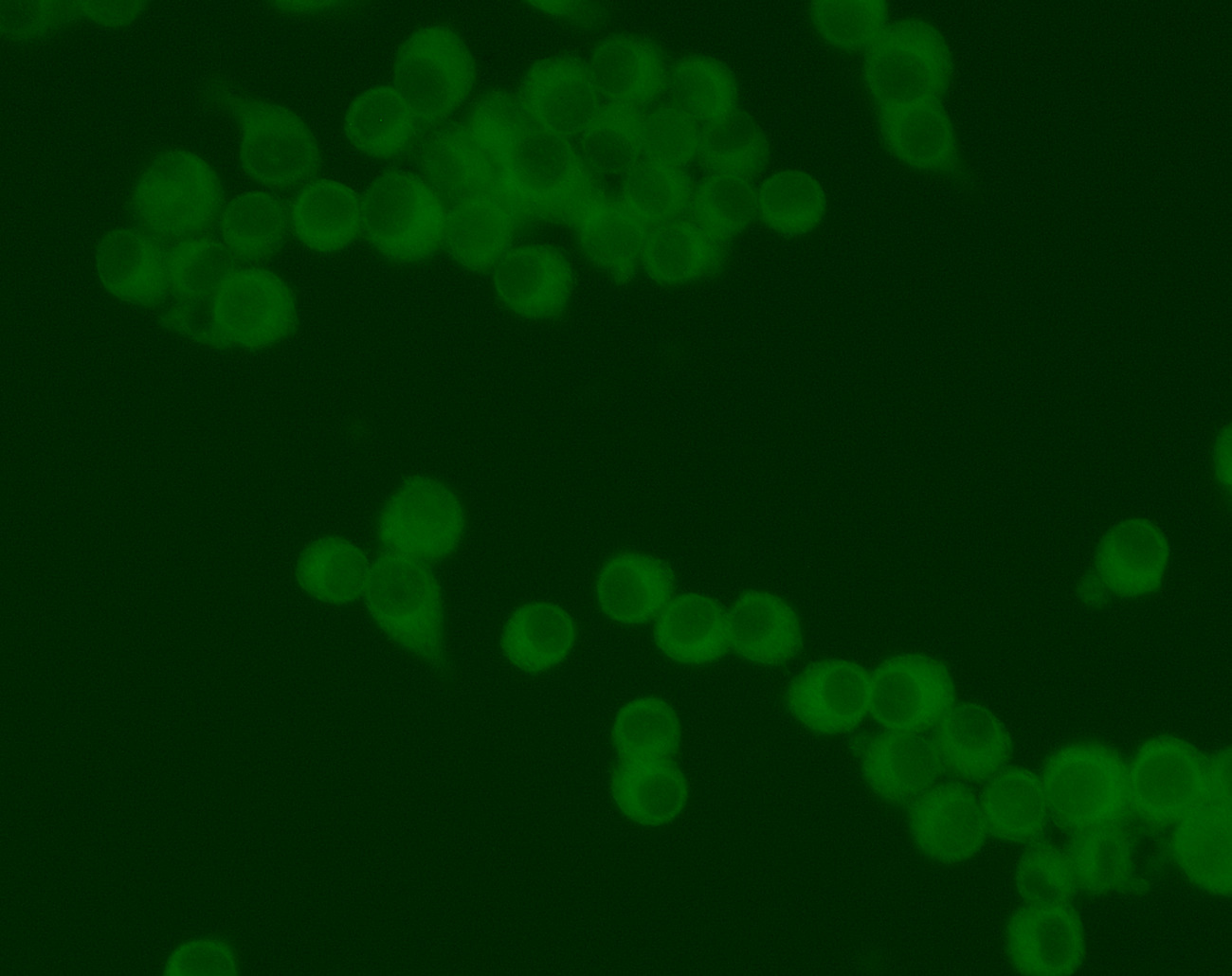

Supplement: Data S8 [file peerj-07-7725-s009.zip › Fig6_IF/06-1_FITC.jpg]

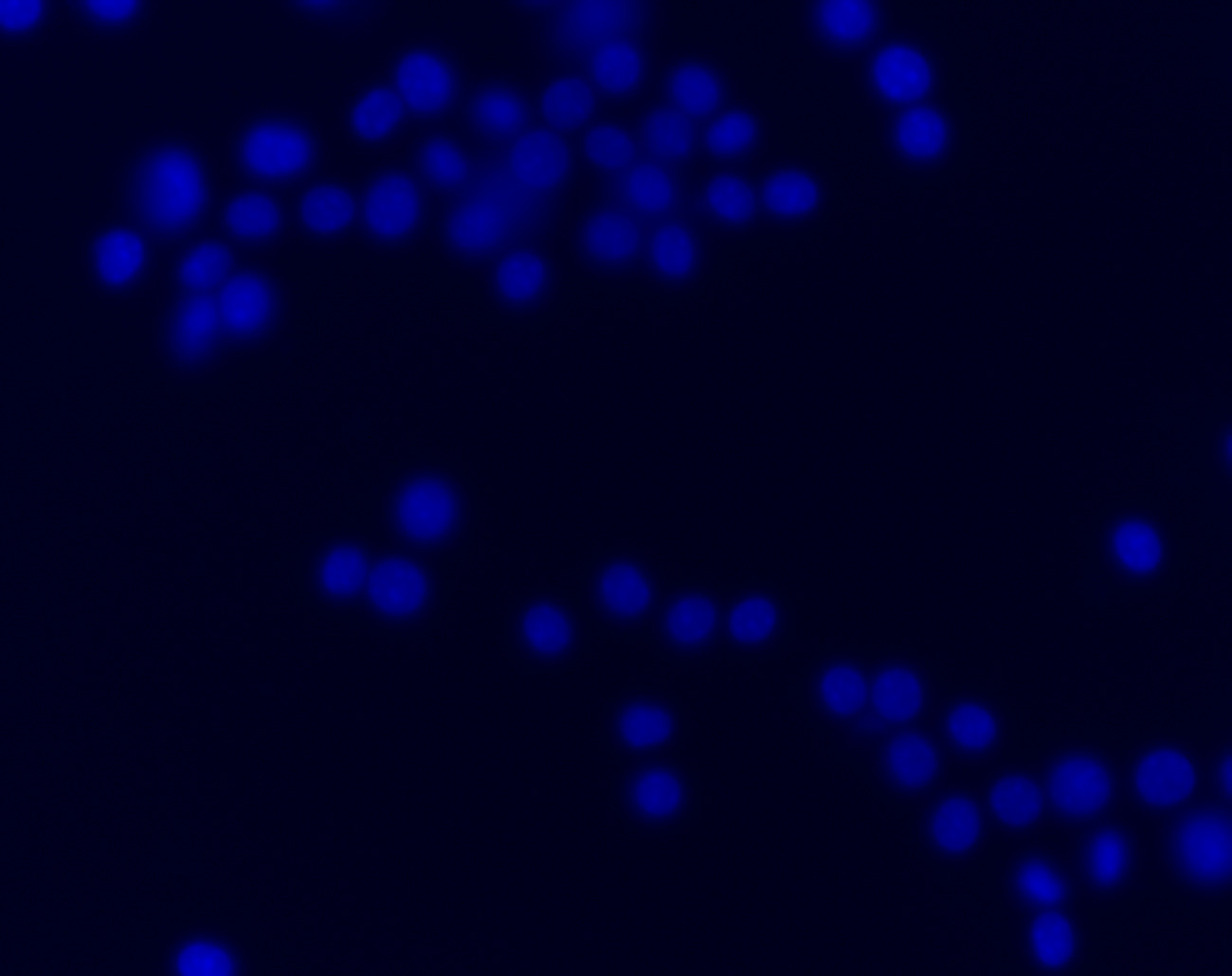

Supplement: Data S8 [file peerj-07-7725-s009.zip › Fig6_IF/06-1_DAPI.jpg]

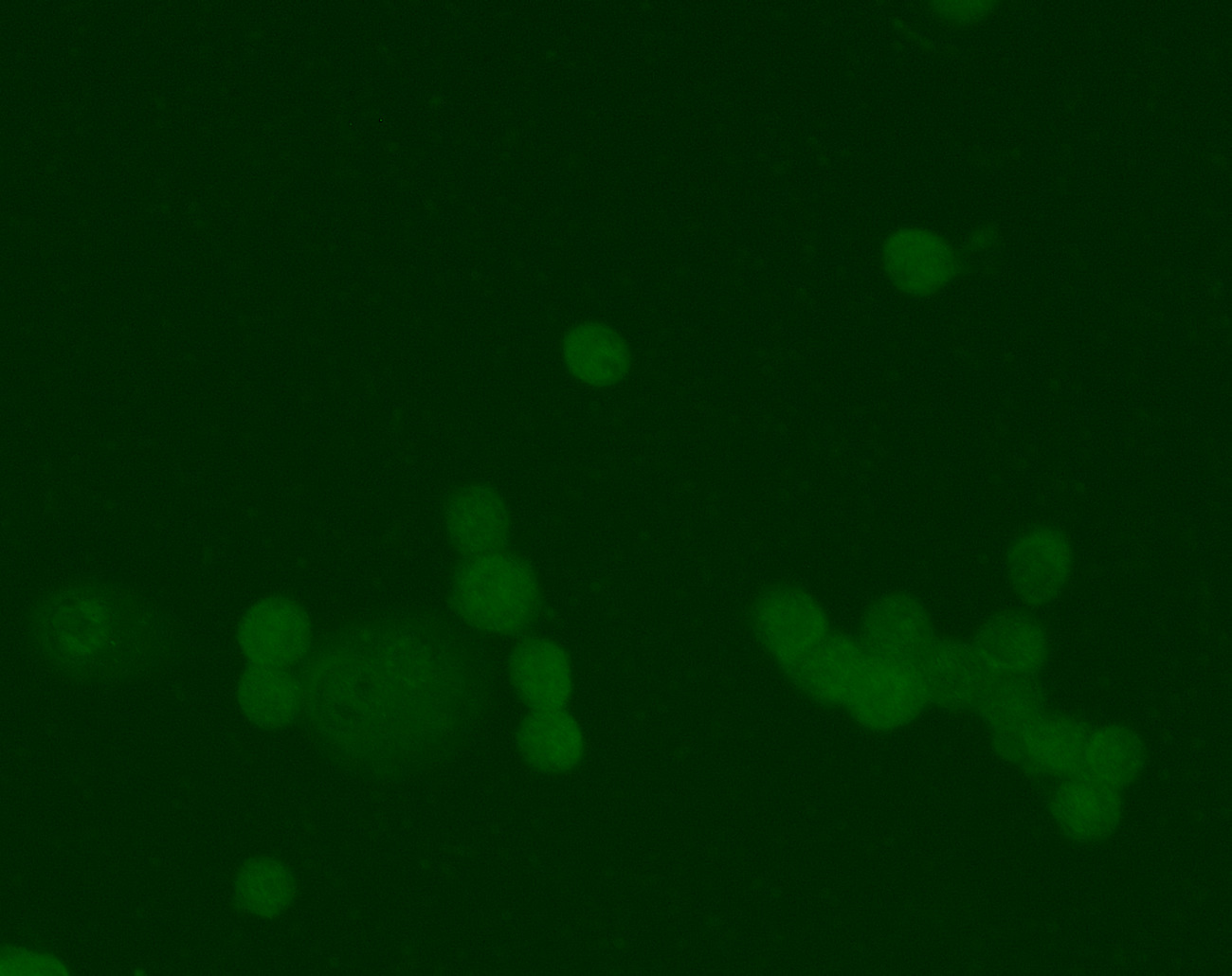

Supplement: Data S8 [file peerj-07-7725-s009.zip › Fig6_IF/08-2_FITC.jpg]

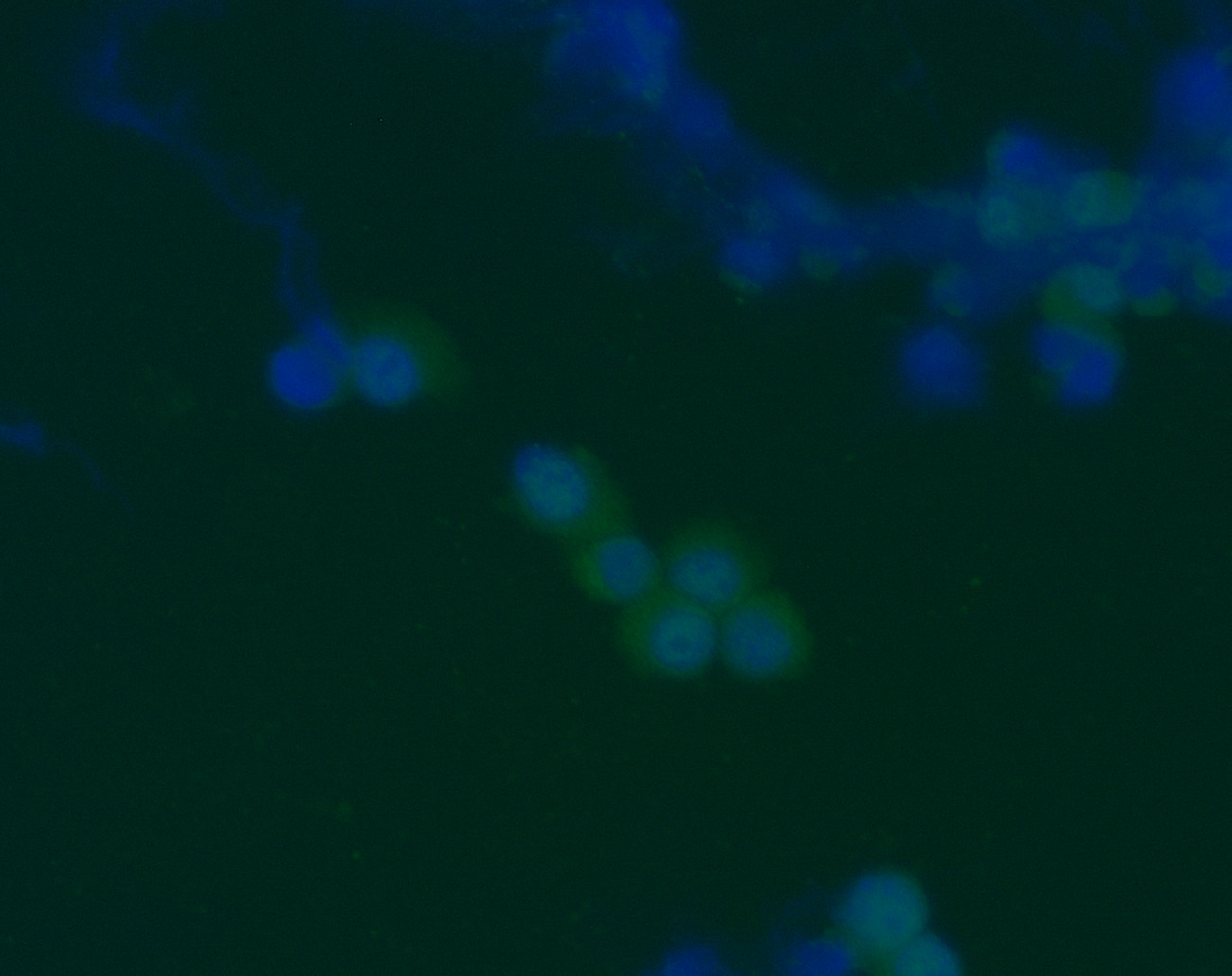

Supplement: Data S8 [file peerj-07-7725-s009.zip › Fig6_IF/07-2_(DAPI+FITC).jpg]

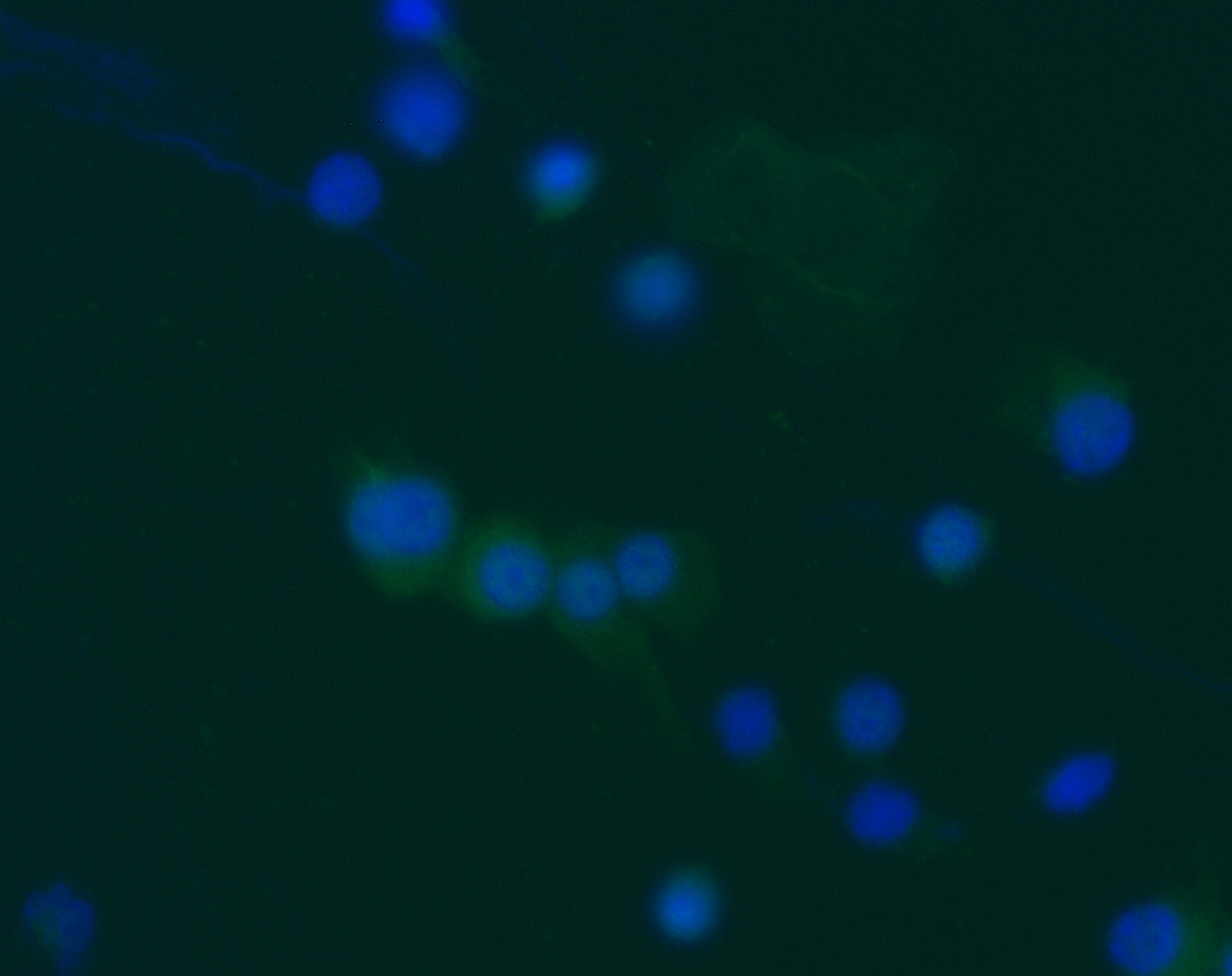

Supplement: Data S8 [file peerj-07-7725-s009.zip › Fig6_IF/10-2_(DAPI+FITC).jpg]

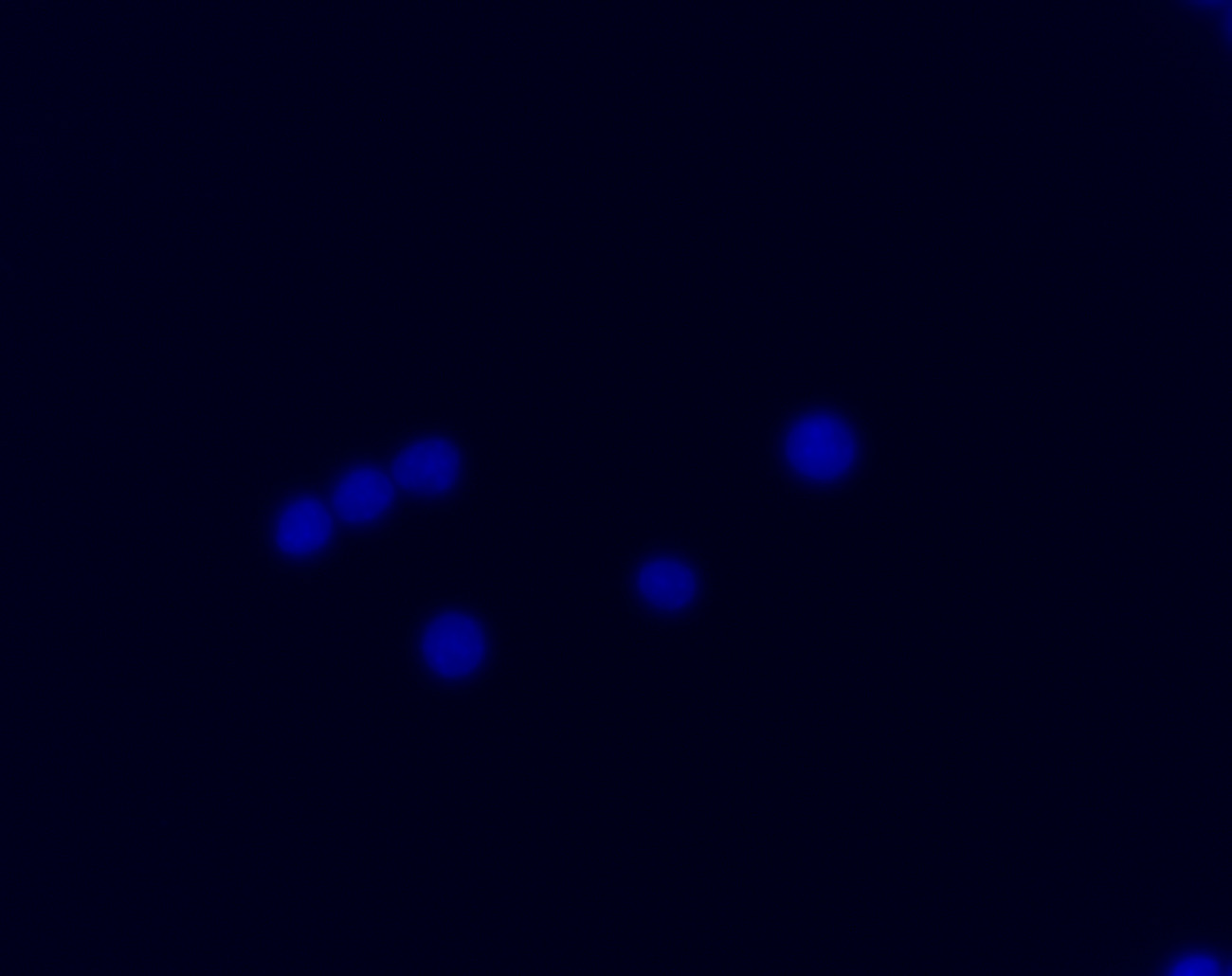

Supplement: Data S8 [file peerj-07-7725-s009.zip › Fig6_IF/09-2_DAPI.jpg]

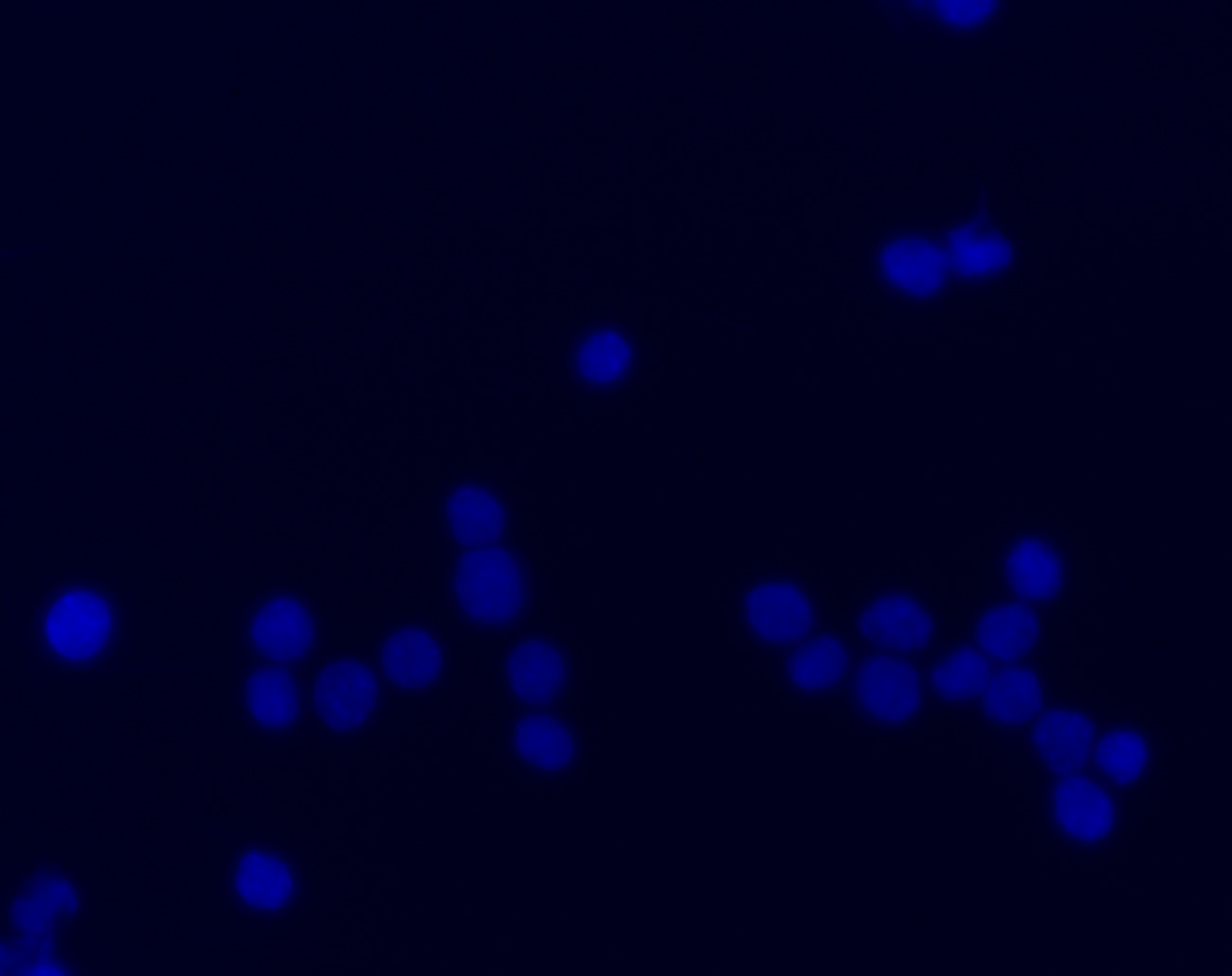

Supplement: Data S8 [file peerj-07-7725-s009.zip › Fig6_IF/08-2_DAPI.jpg]

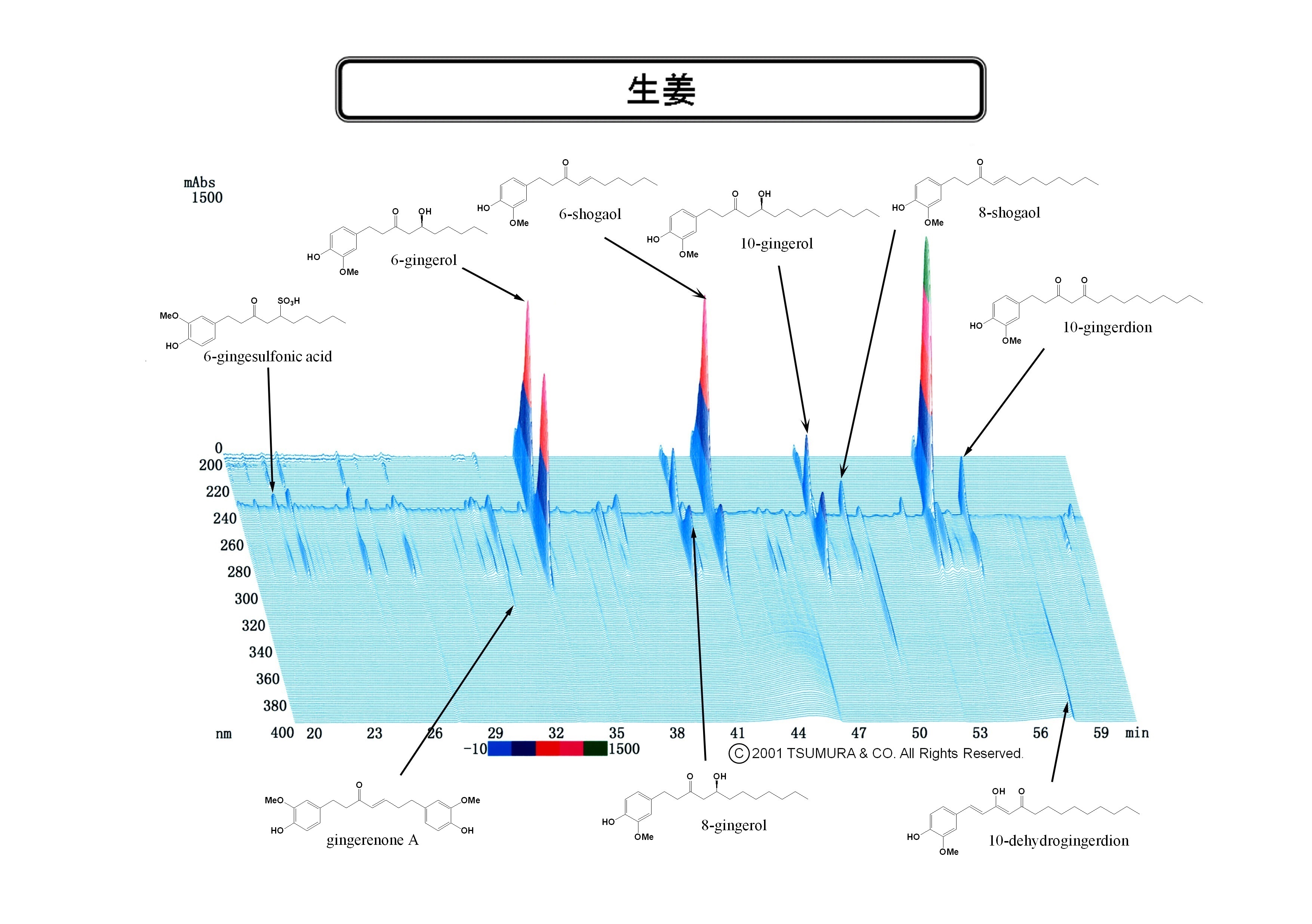

Supplement: Data S9 [file peerj-07-7725-s010.zip › SFig1/profile_shokyo.jpg]

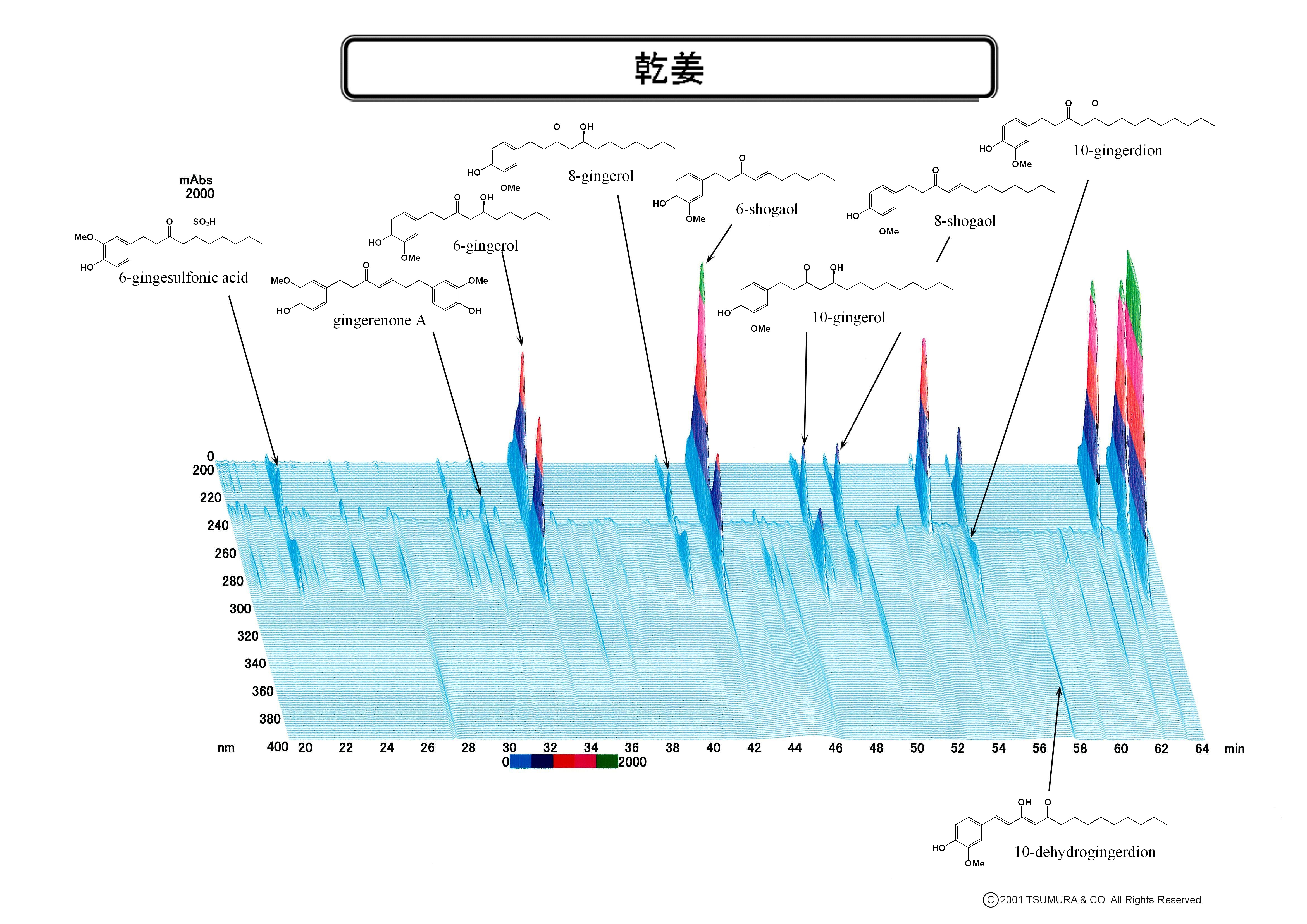

Supplement: Data S9 [file peerj-07-7725-s010.zip › SFig1/profile_kankyo.jpg]

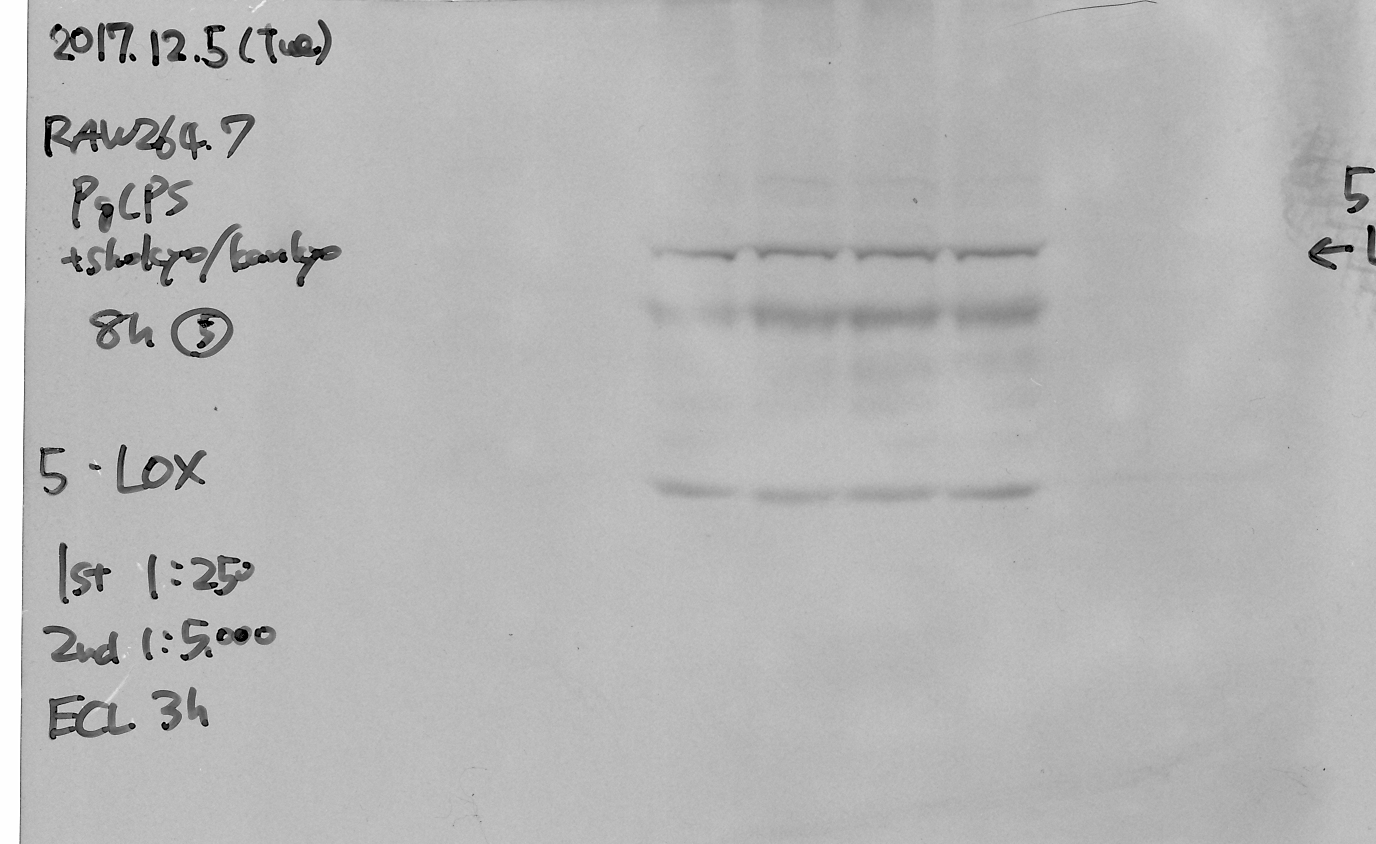

Supplement: Data S10 [file peerj-07-7725-s011.zip › SFig2/LOX5-20171205-1.png]

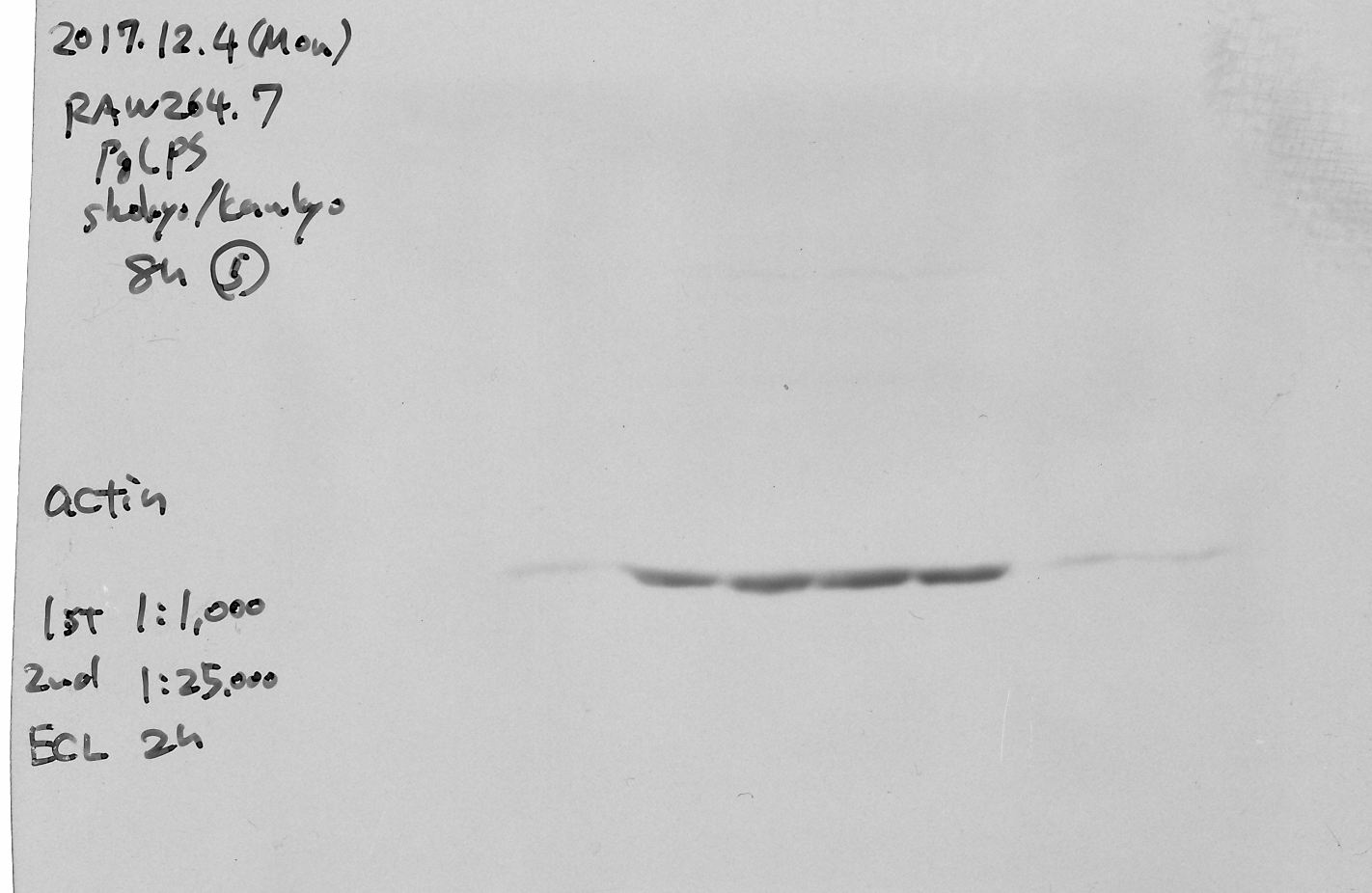

Supplement: Data S10 [file peerj-07-7725-s011.zip › SFig2/actin-20171204-1.png]

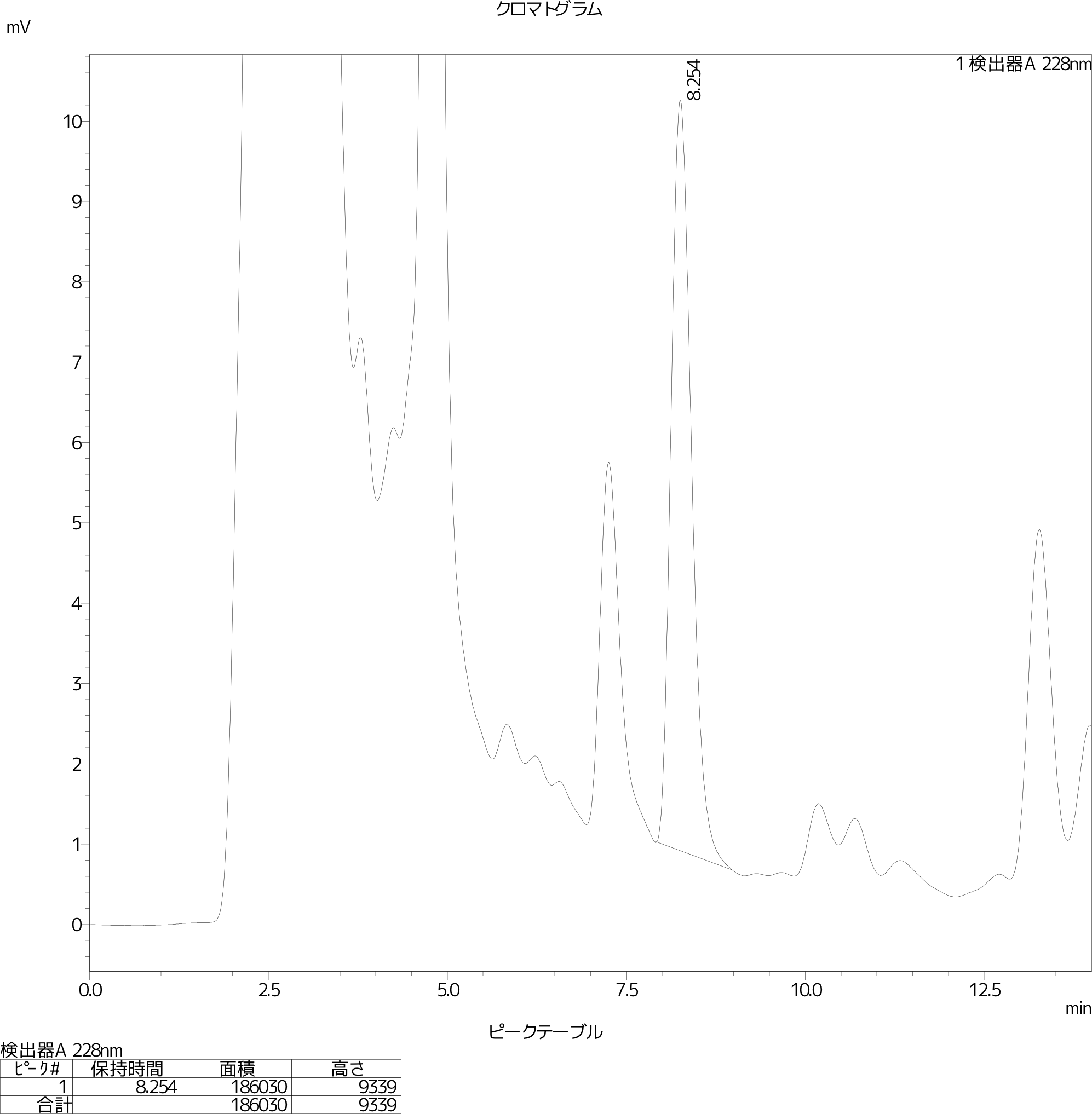

Supplement: Data S10 [file peerj-07-7725-s011.zip › SFig3/shokyo.png]

[6]-Shogaolの分析

検量線

| 濃度 (uM) | ピーク面積   |
|---------|---------|
| 0.1     | 5732    |
| 1       | 62025   |
| 10      | 629091  |
| 50      | 3184242 |

傾き 63729  
切片 -3193.7

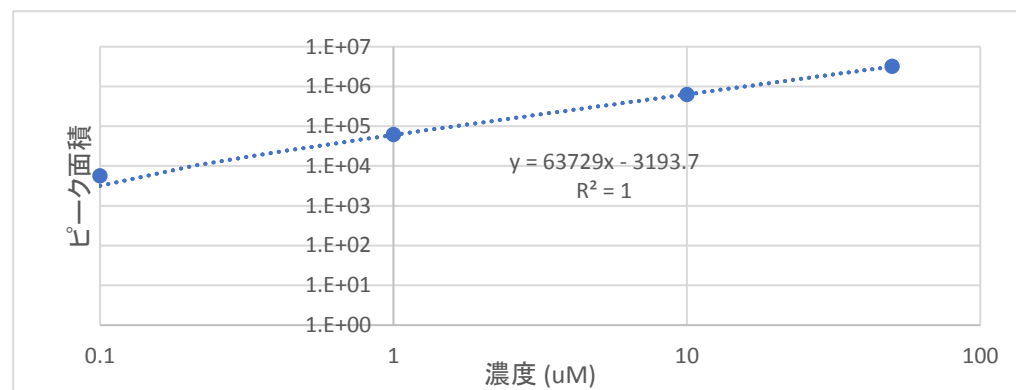

定量結果

|        | 受付番号 | 試料分取量 (mL) | 定容量 (mL) | ピーク面積  | [6]-Shogaol 濃度 (uM) |
|--------|------|------------|----------|--------|---------------------|
| ショウキョウ | 4412 | 1          | 1        | 186030 | 2.97                |
| カンキョウ  | 4413 | 1          | 1        | 307051 | 4.87                |

Supplement: Data S10 [file peerj-07-7725-s011.zip › SFig3/6-Shogaol_Ja.pdf]

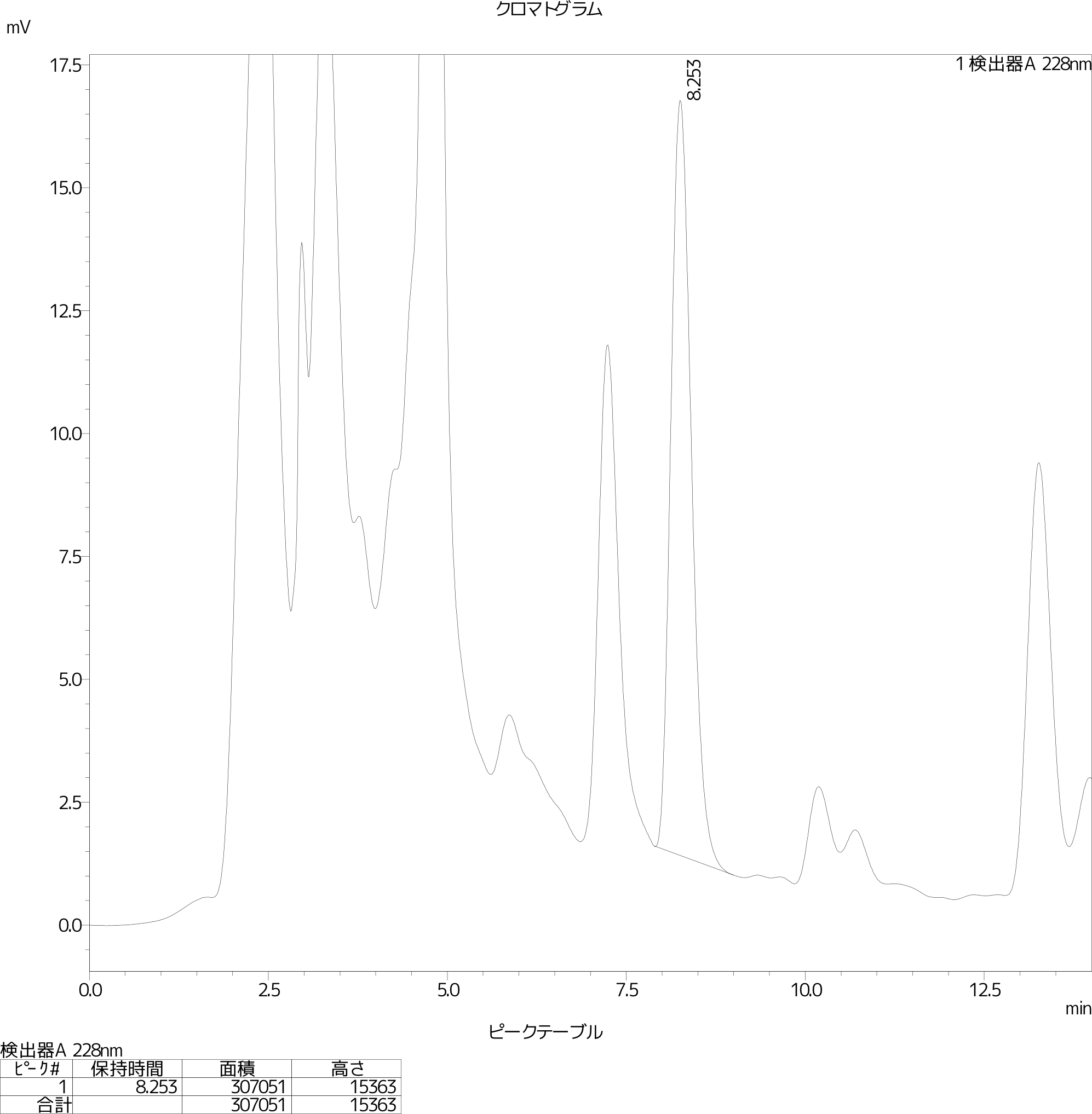

Supplement: Data S10 [file peerj-07-7725-s011.zip › SFig3/kankyo.png]
