# Supplementary material for: Effects of shokyo (Zingiberis Rhizoma) and kankyo (Zingiberis Processum Rhizoma) on prostaglandin E2 production in lipopolysaccharide-treated mouse macrophage RAW264.7 cells
Source: PeerJ. 2019 Sep 17;7:e7725. doi: 10.7717/peerj.7725 (PMC6753926; doi:10.7717/peerj.7725)
Supplement: Data S10 [file peerj-07-7725-s011.zip › SFig2/Analysis_herb_LOXactivity.pdf]

Statistical analysis

Toshiaki Ara

LOX activity

simultanous treatment

filename

030_herb_LTB4_5_raw.csv

multiple comparison by Dunnett method

```
     Simultaneous Tests for General Linear Hypotheses

Multiple Comparisons of Means: Dunnett Contrasts


Fit: lm(formula = adjusted ~ drug1, data = dat1)

Linear Hypotheses:
            Estimate Std. Error t value Pr(>|t|)
3 - 2 == 0 0.0003691  0.0002169   1.702    0.230
4 - 2 == 0 0.0003256  0.0002169   1.501    0.298
(Adjusted p values reported -- single-step method)
```

sequential treatment

filename

030_herb_LTB4_6_raw.csv

multiple comparison by Dunnett method

```
     Simultaneous Tests for General Linear Hypotheses

Multiple Comparisons of Means: Dunnett Contrasts


Fit: lm(formula = adjusted ~ drug1, data = dat1)
```

```
Linear Hypotheses:
             Estimate Std. Error t value Pr(>|t|)
3 - 2 == 0 -0.0005754  0.0002887  -1.993    0.157
4 - 2 == 0 -0.0001710  0.0002887  -0.592    0.786
(Adjusted p values reported -- single-step method)
```
