# Supplementary material for: Effects of shokyo (Zingiberis Rhizoma) and kankyo (Zingiberis Processum Rhizoma) on prostaglandin E2 production in lipopolysaccharide-treated mouse macrophage RAW264.7 cells
Source: PeerJ. 2019 Sep 17;7:e7725. doi: 10.7717/peerj.7725 (PMC6753926; doi:10.7717/peerj.7725)
Supplement: Data S10 [file peerj-07-7725-s011.zip › SFig2/030_herb_LTB4-5-AA.pdf]

- Exp. 30
- Condition
  - drug1: herb ()
  - experimental No. 5
  - treatment: 24h
- Measurement
  - LTB4
  - Date: 2017.11.24
- Cells
  - cells: RAW264.7, passages: NA
  - cell numbers: 5×10^4 cells/well = 25×10^4 cells/ml

	conc.	OD
1	15.6	1.191
2	31.2	1.010
3	62.5	0.861
4	125.0	0.618
5	250.0	0.446
6	500.0	0.355
7	1000.0	0.282
8	2000.0	0.248

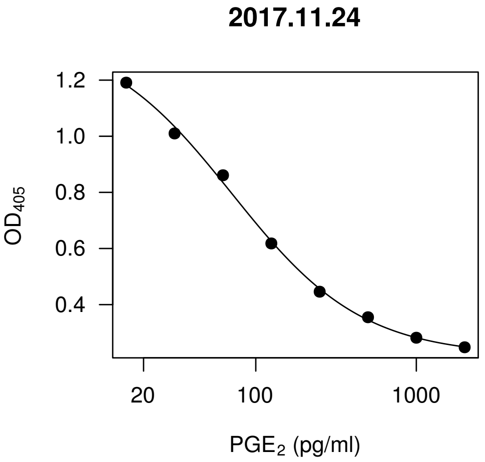

	drug1	mean	SD
1	1	0.001	0.000
2	2	0.001	0.000
3	3	0.002	0.000
4	4	0.002	0.000

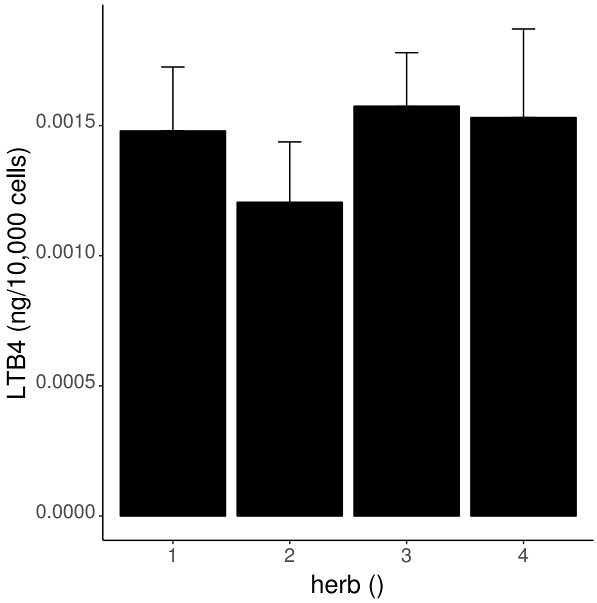

	drug1	viability	dilution	OD	conc. (pg/ml)	net (ng/ml)	(ng/10,000 cells)
1	2	100.28	1	1.101	23.61	0.024	0.001
2	2	100.16	1	1.024	32.47	0.032	0.001
3	2	100.52	1	1.007	34.63	0.035	0.001
4	3	101.48	1	0.989	37.01	0.037	0.001
5	3	99.56	1	0.933	45.10	0.045	0.002
6	3	101.12	1	0.991	36.74	0.037	0.001
7	4	96.80	1	1.019	33.09	0.033	0.001
8	4	100.76	1	1.021	32.84	0.033	0.001
9	4	98.12	1	0.920	47.15	0.047	0.002
10	1	100.88	1	1.001	35.41	0.035	0.001
11	1	98.72	1	1.031	31.60	0.032	0.001
12	1	100.40	1	0.940	44.02	0.044	0.002
