# Supplementary material for: Effects of shokyo (Zingiberis Rhizoma) and kankyo (Zingiberis Processum Rhizoma) on prostaglandin E2 production in lipopolysaccharide-treated mouse macrophage RAW264.7 cells
Source: PeerJ. 2019 Sep 17;7:e7725. doi: 10.7717/peerj.7725 (PMC6753926; doi:10.7717/peerj.7725)
Supplement: Data S10 [file peerj-07-7725-s011.zip › SFig2/030_herb_LTB4-6-AA.pdf]

- Exp. 30
- Condition
  - drug1: herb ()
  - experimental No. 6
  - treatment: 24h
- Measurement
  - LTB4
  - Date: 2017.11.24
- Cells
  - cells: RAW264.7, passages: NA
  - cell numbers: 5×10^4 cells/well = 25×10^4 cells/ml

	conc.	OD
1	15.6	1.191
2	31.2	1.010
3	62.5	0.861
4	125.0	0.618
5	250.0	0.446
6	500.0	0.355
7	1000.0	0.282
8	2000.0	0.248

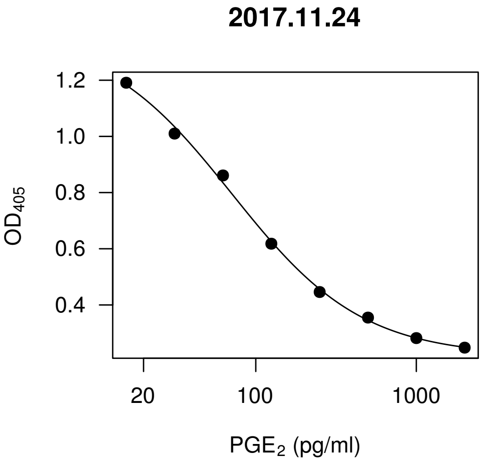

	drug1	mean	SD
1	1	0.002	0.000
2	2	0.002	0.001
3	3	0.001	0.000
4	4	0.002	0.000

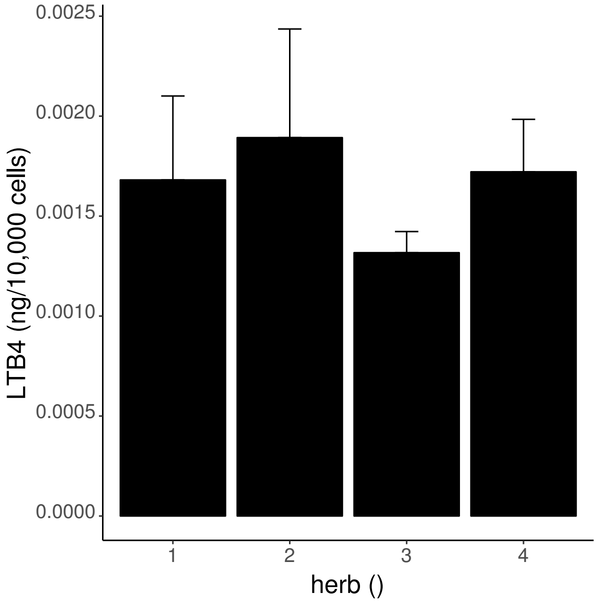

	drug1	viability	dilution	OD	conc. (pg/ml)	net (ng/ml)	(ng/10,000 cells)
1	2	96.34	1	0.847	60.08	0.060	0.002
2	2	96.46	1	0.953	42.08	0.042	0.002
3	2	98.09	1	1.002	35.28	0.035	0.001
4	3	98.34	1	1.038	30.75	0.031	0.001
5	3	97.34	1	1.004	35.02	0.035	0.001
6	3	97.84	1	1.037	30.87	0.031	0.001
7	4	98.46	1	1.001	35.41	0.035	0.001
8	4	98.71	1	0.913	48.28	0.048	0.002
9	4	99.83	1	0.939	44.18	0.044	0.002
10	1	100.33	1	0.883	53.37	0.053	0.002
11	1	97.84	1	0.970	39.63	0.040	0.002
12	1	101.83	1	1.020	32.97	0.033	0.001
